# ZnFe_2_O_4_@SiO_2_@AC Magnetic Nanocomposite as an Efficient Catalyst for Ultrasound‐Assisted Azidation of Aryl Halides to Aryl Amines

**DOI:** 10.1002/open.70172

**Published:** 2026-03-29

**Authors:** Manish Kumar, Rima Heider Al Omari, Soumya V. Menon, Shaker Al‐Hasnaawei, Ahmad Sajjadi, Amrita Pal, Renu Sharma, Aashna Sinha

**Affiliations:** ^1^ Department of Electronics and Communication Engineering GLA University Mathura India; ^2^ Faculty of Allied Medical Sciences Hourani Center for Applied Scientific Research Al‐Ahliyya Amman University Amman Jordan; ^3^ Department of Chemistry and Biochemistry School of Sciences JAIN (Deemed to be University) Bangalore India; ^4^ College of pharmacy the Islamic University Najaf Iraq; ^5^ Department of medical analysis Medical laboratory technique college the Islamic University of Al Diwaniyah Al Diwaniyah Iraq; ^6^ Young Researchers and Elite Club, Tehran Branch Islamic Azad University Tehran Iran; ^7^ Department of Chemistry Sathyabama Institute of Science and Technology Chennai India; ^8^ Department of Chemistry University Institute of Sciences Chandigarh University Mohali India; ^9^ School of Applied and Life Sciences Division of Research and Innovation Uttaranchal University Dehradun Indian

**Keywords:** aryl amines, aryl halides, azidation, heterogeneous catalyst, magnetic mesoporous, ultrasound

## Abstract

In this study, a novel ZnFe_2_O_4_@SiO_2_@AC nanocomposite catalyst was synthesized, demonstrating a reliable approach that underscores its innovative potential in the azidation of aryl halides, followed by their conversion to aryl amines. The catalytic system operates efficiently in ethanol under ultrasound irradiation at 60°C, providing a green, sustainable alternative to conventional synthetic methods. The core–shell structure of ZnFe_2_O_4_@SiO_2_@AC enhances catalytic activity and stability, facilitating high yields of the target aryl amines with excellent selectivity. Techniques employed to characterize the resulting nanocatalyst included X‐ray diffraction, Fourier transform infrared, transmission electron microscopy, vibrating‐sample magnetometry, and Brunauer–Emmett–Teller surface area analysis, ensuring characterization of its structural, magnetic, and surface properties. Notably, the catalyst exhibited excellent recyclability, maintaining catalytic activity over six successive cycles with minimal deactivation, highlighting its robustness and suitability for sustainable catalytic applications in organic synthesis. The use of ultrasound conditions provides improved mass transfer and reaction kinetics, reducing reaction time and energy consumption, further enhancing the ecofriendly nature of the process. This work highlights the potential of multifunctional nanocomposites in ecofriendly organic transformations and underscores the role of sonochemistry in promoting efficient catalytic processes.

## Graphical abstract

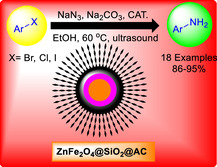

## 1 Introduction

Aryl amines, a fundamental class of organic compounds characterized by an amino group (–NH_2_) directly attached to an aromatic ring, occupy a central place in modern chemical science and industry [1, 2, 3]. Their ubiquity and wide range of applications render them indispensable building blocks across disciplines: in pharmaceutical chemistry, the aryl amine moiety forms the backbone of countless active pharmaceutical ingredients (APIs), contributing crucial hydrogen‐bonding interactions that enable strong binding to biological targets and often serving as a privileged pharmacophore in medicinal chemistry. The amino group, a key component of aryl amines, plays a significant role in drug–target interactions, reinforcing the economic and scientific impetus for sustainable, efficient routes to aryl amines [4, 5, 6]. This structural motif underpins lifesaving drugs such as sulfa antibiotics, exemplified by sulfamethoxazole, nonsteroidal anti‐inflammatory drugs like paracetamol, and a broad spectrum of anticancer and antiviral agents. The significance of the amino group extends beyond direct pharmacology; it supports the pharmacophoric framework that governs drug–target interactions, reinforcing the economic and scientific impetus for sustainable, efficient routes to aryl amines [7, 8, 9, 10].

Beyond their medical relevance, aryl amines are versatile compounds with applications in various fields. They are essential precursors to azo dyes and pigments, the largest and most versatile class of dyes employed across textiles, food, and cosmetics [11, 12, 13, 14, 15]. In materials science, aryl amines serve as key monomers for high‐performance polymers such as polyimides and aramids, including Kevlar. Moreover, they function as ligands in catalysis and as intermediates in the synthesis of diverse fine chemicals, agrochemicals, and other specialty compounds. This broad utility creates a persistent demand for efficient, sustainable, and cost‐effective methods to assemble arylamine structures [16, 17, 18, 19, 20].

Historically, the most direct route to aryl amines has been nucleophilic aromatic substitution (SNAr) of aryl halides. However, SNAr is fundamentally constrained by substrate electronics: it is generally effective only on electron‐deficient arenes bearing substituents such as nitro or cyano groups ortho or para to the halide, which stabilize the Meisenheimer intermediate [21, 22, 23]. This reliance on activation severely narrows the substrate scope, leaving electron‐neutral and electron‐rich aryl halides—which are abundant and valuable—unamenable to SNAr under conventional conditions. The limitations of SNAr catalyzed the development of catalytic crosscoupling strategies, most notably the Buchwald–Hartwig amination, a palladium‐catalyzed process that couples aryl halides or pseudohalides with amines in the presence of sophisticated phosphine ligands. This transformation represents a watershed moment in C—N bond formation, enabling amination of a vast array of aryl substrates under comparatively milder conditions than SNAr [24, 25, 26]. However, the Buchwald–Hartwig approach is not without drawbacks. It relies on expensive, air‐ and moisture‐sensitive palladium catalysts and ligands, raising concerns about residual metal impurities in APIs, and generates substantial amounts of metal halide waste that complicates waste management and the catalyst life cycle, particularly with challenging substrates. These challenges highlight the need for alternative, more sustainable methods [27, 28, 29]. A compelling two‐step alternative has gained attention: conversion of an aryl halide to an aryl azide, followed by reduction to the corresponding aryl amine. This azidation–reduction sequence decouples C—N bond formation from a single metal‐catalyzed process, offering the potential for milder conditions and broader substrate compatibility and aligning well with the rich chemistry of aryl azides, including Staudinger reductions and Huisgen 1,3‐dipolar cycloadditions [30, 31, 32, 33].

The azidation step, however, presents challenges in terms of safety and practicality. Conventional ionic azidation using sodium azide (NaN_3_) on unactivated aryl halides is notorious for its inefficiency and hazards, often requiring elevated temperatures and extended reaction times, while posing significant explosion risks due to the formation of hydrazoic acid (HN_3_) [34, 35, 36, 37]. Even with advances in copper‐catalyzed azidation employing azide sources such as trimethylsilyl azide (TMSN_3_), safety concerns persist, as do reagent costs, moisture sensitivity, and solvent requirements. Consequently, delivering a safe, scalable, and cost‐effective azidation step remains a central hurdle in the azidation–reduction paradigm for aryl amine synthesis [38, 39, 40, 41, 42].

Parallel to advancements in catalyst development, sonochemistry has emerged as a powerful technique to accelerate and improve chemical reactions by employing ultrasound irradiation [43]. Ultrasound produces cavitation phenomena—namely, the formation, growth, and implosive collapse of bubbles—leading to localized hot spots with extremely high temperatures and pressures that promote the generation of reactive species and enhanced mass transfer [44]. Applications of ultrasound irradiation have been shown to significantly reduce reaction times, improve yields, and enable milder reaction conditions in various organic syntheses [45]. Ultrasound‐assisted reactions in environmentally benign solvents, such as ethanol, further align with the principles of green chemistry by reducing reliance on hazardous reagents and minimizing energy consumption [46].

Despite the distinct advantages of ZnFe_2_O_4_‐based nanocomposites and ultrasound‐assisted methodologies, their combined application in the azidation of aryl halides followed by reduction to aryl amines has not been thoroughly explored. The azidation reaction, which involves the formation of aryl azides as intermediates, represents a versatile approach to synthesizing aryl amines via subsequent Staudinger reduction or hydrogenation. The challenge lies in achieving selective azidation under mild, green conditions with efficient catalyst performance.

This research presents a novel approach employing a ZnFe_2_O_4_@SiO_2_@AC nanocomposite catalyst for the azidation of aryl halides in ethanol under ultrasound irradiation at 60°C. The study focuses on the synthesis and characterization of this multifunctional nanocomposite and its catalytic performance in facilitating efficient, selective, and sustainable azidation, leading to the formation of aryl amines. The ZnFe_2_O_4_@SiO_2_@AC nanocomposite catalyst plays a crucial role in this process, providing a green, reusable, and effective catalytic platform for the synthesis of valuable aryl amines. The underlying mechanisms of catalysis and the synergistic effects of ultrasound‐assisted reaction conditions are also critically analyzed. This work aims to address the limitations of conventional methods by providing a green, reusable, and effective catalytic platform for the synthesis of valuable aryl amines, with implications for pharmaceutical and fine chemical manufacturing.

## 2 Result and Discussion

**SCHEME 1:**
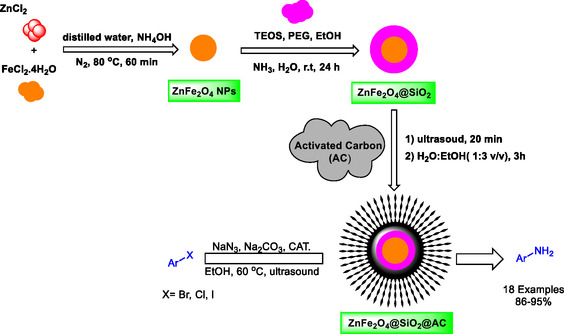
General approach for preparation of ZnFe_2_O_4_@SiO_2_@AC NP in catalytic amination of haloarenes by sodium azide.

This study advances the promising concept of azidating aryl halides to aryl amines under ultrasound, using a recyclable ZnFe_2_O_4_@SiO_2_@AC magnetic nanocomposite as the mediator. Although detailed experimental procedures are limited in the current literature, the potential and effectiveness of magnetic nanoparticle (NP) catalysts are supported by related research on transformations such as epoxidation (Scheme 1). Highlighting this catalyst's capabilities underscores its potential for sustainable, green applications with significant industrial relevance, particularly in the synthesis of aryl amines.

Central to the work is the synthesis of a novel ZnFe_2_O_4_@SiO_2_@AC nanocomposite via a robust three‐step protocol presented in Scheme 1. This sequence reliably produces nanocatalysts with high stability and magnetic recoverability, reassuring researchers of its practical robustness. The experimental section provides detailed, reproducible procedures—including reagent proportions, reaction conditions, and purification steps—to enable other researchers to reproduce and potentially optimize the system. By integrating a magnetically recoverable core, a chemically stable silica shell, and an AC surface, the design emphasizes enhanced dispersion and catalytic activity while enabling facile separation and reuse. The result is a sustainable, economically viable catalytic platform that aligns with the broader goals of green chemistry, reducing waste and operating costs through recyclability and magnetically assisted recovery without compromising catalytic performance.

The synthesis of the nanocatalyst involves several preparatory steps that ensure the successful formation of ZnFe_2_O_4_ NPs supported on AC. The choice of zinc chloride (ZnCl_2_) and iron(III) chloride hexahydrate (FeCl_3_ · 6H_2_O) as the starting materials is based on their ability to form the desired ZnFe_2_O_4_ NPs. These are reacted in a mixture of distilled water and ammonia hydroxide (NH_4_OH) at a temperature of 80°C for 60 min under a nitrogen (N_2_) atmosphere to promote the formation of ZnFe_2_O_4_ NPs. Following this, the sol–gel method is employed by adding tetraethyl orthosilicate (TEOS), polyethylene glycol (PEG), and ethanol (EtOH) to the resulting suspension. The choice of these materials is based on their roles in creating a stable suspension for the next phase of the synthesis process, allowing it to react at room temperature for 24 h.

Subsequently, an ultrasonication process is performed for 20 min to facilitate the dispersion and interaction of the NPs with the AC. This is followed by the careful mixing of the as‐synthesized ZnFe_2_O_4_@SiO_2_ composite with AC in an ethanol solvent system to achieve a volumetric ratio of 1:3 (v/v). The mixture is then allowed to react for 3 h. Through this careful orchestration of steps, the ZnFe_2_O_4_@SiO_2_@AC nanocatalyst is obtained, offering enhanced catalytic properties suitable for various applications.

**FIGURE 1:**
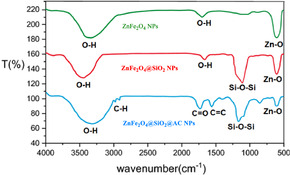
FTIR spectra of ZnFe_2_O_4_, ZnFe_2_O_4_@SiO_2_, and ZnFe_2_O_4_@SiO_2_@AC nanocomposite.

Figure 1 shows the Fourier transform infrared (FTIR) spectra of three samples in the order of progressive surface modification: ZnFe_2_O_4_ NPs, ZnFe_2_O_4_ coated with SiO_2_ (ZnFe_2_O_4_@SiO_2_ NPs), and the composite ZnFe_2_O_4_@SiO_2_@AC NPs. The spectra are plotted as transmittance (*T*%) versus wave number from 4000 to 500 cm^−1^, and the bands are annotated in the figure to highlight the characteristic functional groups associated with each component. The comparison of these spectra provides a coherent picture of how the surface chemistry evolves from the bare oxide to the silica shell and finally to the carbon‐functionalized shell, and it also serves to confirm the integrity of the oxide core throughout the functionalization sequence.

In the pristine ZnFe_2_O_4_ sample, the spectrum is dominated by features typical of metal‐oxide surfaces. A broad, prominent band assigned to O–H stretching appears around 3400 cm^−1^, accompanied by a lower‐frequency O–H bending region near 1600 cm^−1^, which together indicate the presence of adsorbed water and surface hydroxyl groups. This spectral region reflects the hygroscopic and hydroxylated nature of the bare oxide surface. In addition to these surface‐adsorbed species, the low‐frequency region of the spectrum exhibits bands consistent with metal–oxygen lattice vibrations, commonly attributed to Zn–O and Fe–O linkages in spinel ferrites. These bands, located in the region around 500–600 cm^−1^, confirm the integrity of the oxide core. Overall, the green spectrum thus documents a clean ZnFe_2_O_4_ core with surface hydroxyls and characteristic metal‐oxide vibrations.

Introduction of a silica shell shifts the spectral signature noticeably, as seen in the red spectrum for ZnFe_2_O_4_@SiO_2_ NPs. A new, strong band emerges near 1100 cm^−1^, which is the hallmark of Si—O—Si asymmetric stretching present in a SiO_2_ network. This peak confirms successful coating with silica. A second silica‐related feature appears in the region around 800–1000 cm^−1^, corresponding to Si—O—Si bending modes, further corroborating the formation of a siliceous shell on the ZnFe_2_O_4_ core. While the SiO_2_ layer is evident, the O–H band around 3400 cm^−1^ persists, albeit with reduced intensity relative to the bare oxide, indicating that surface silanol (Si–OH) groups and adsorbed water remain on the silica surface. Importantly, the core oxide signals (Zn–O/Fe–O) are still present but may be somewhat attenuated due to overcoating by the silica shell, consistent with the expected dilution of the oxide vibrations by the added silica layer.

The blue spectrum, representing ZnFe_2_O_4_@SiO_2_@AC NPs, shows the anticipated continuation of the transformation, now with signatures arising from the AC component. The AC plays a crucial role in the final stage of the surface modification, contributing to the unique properties of the hierarchical composite. In addition to the persistent Si—O—Si bands around 1100 cm^−1^ and the silica‐related bending modes, new bands appear corresponding to carbonaceous material: a C—H stretching band near 2900 cm^−1^ and a band assignable to C=C or aromatic C=C vibrations in the ∼1600 cm^−1^ region. The persistence of the Si—O—Si features confirms the continued presence of the silica framework, while the emergence of new C—H and C=C bands provides clear evidence for the successful incorporation of AC onto the silica surface, yielding a ZnFe_2_O_4_@SiO_2_@AC architecture. The Zn–O/Fe–O signals, while likely still present, may exhibit further attenuation due to the overlying carbon layer, yet remain detectable, indicating that the magnetic oxide core remains intact within the hierarchical composite.

When these three spectra are compared side by side, the data collectively support a straightforward, stepwise functionalization strategy. The pristine ZnFe_2_O_4_ exhibits standard oxide and hydroxyl features; the silica coating introduces distinctive Si—O—Si network bands at ∼1100 and 800–1000 cm^−1^, along with a modest reduction in surface –OH signals. These changes in the FTIR spectra are significant as they confirm the successful synthesis of the intended materials. Subsequent carbon functionalization adds characteristic aliphatic and aromatic carbon bands around 2900 and 1600 cm^−1^, while preserving the silica framework and the underlying oxide bands. The progression of spectral features thus confirms the successful synthesis of the intended materials, and the spectral changes are consistent with a coherent picture of a magnetic ZnFe_2_O_4_ core that is progressively encapsulated first by silica and then by AC, yielding a hierarchical, functionalized NP suitable for further applications.

**FIGURE 2:**
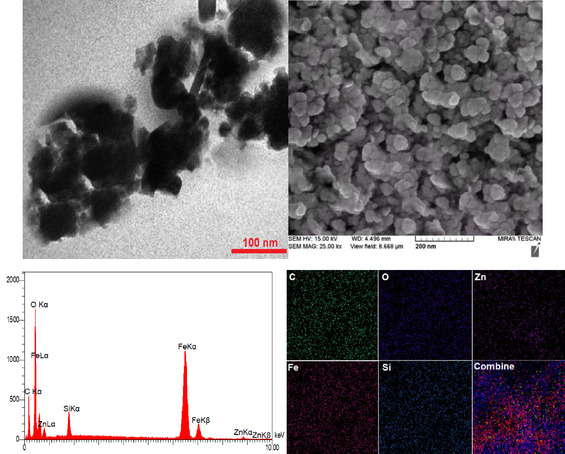
TEM and SEM images and EDX and elemental mapping analysis of ZnFe_2_O_4_@SiO_2_@AC nanocomposite.

Figure 2 provides complementary microscopy (TEM [transmission electron microscopy] and SEM [scanning electron microscopy]) and spectroscopic (EDX [energy‐dispersive X‐ray]) evidence for the architecture of ZnFe_2_O_4_@SiO_2_@AC nanocomposites. The TEM image (left) reveals nanoscale crystallites forming a dark, dense core with surrounding lighter material, consistent with a multilayer particle. Individual crystallites are typically 8–15 nm, with occasional small clusters extending to about 20–40 nm, indicating partial agglomeration driven by magnetic interactions during drying. The boundary between the dense interior and the more diffuse exterior supports a core–shell arrangement, where the core is a ZnFe_2_O_4_‐like magnetic phase, the shell is a silica (SiO_2_) layer, and the outer coating comprises AC. The SEM image (right) shows a broader, surface‐focused view of a densely packed network of roughly spherical entities, yielding a rough, porous morphology typical of carbon‐rich materials augmented by inorganic cores. Particle sizes inferred from SEM range from tens to a few hundred nanometers, with many features under 100 nm, reflecting agglomeration tendencies during ambient drying and confirming the presence of AC‐rich surface features that enhance porosity and surface area. These microscopy techniques, combined with EDX spectroscopy, provide a comprehensive validation of the nanocomposite structure.

From a holistic perspective, the TEM and SEM observations are consistent with the intended ZnFe_2_O_4_@SiO_2_@AC architecture, reinforcing the design's reliability for the audience. TEM provides direct evidence of nanoscale core–shell composition, while SEM shows that these particles assemble into a porous, mesoscopic network with substantial interparticle contacts. The convergence of these data validates a crystalline ZnFe_2_O_4_ core encapsulated by an amorphous SiO_2_ shell and further decorated with an outer AC layer, enabling magnetic separability alongside high surface area and porosity. Minor agglomeration observed in both images is typical for ferrite NPs and can be mitigated by optimizing synthesis and drying protocols without compromising the multilayer structure.

Figure 2 also presents the crucial EDX spectrum (0–10 keV), which confirms the composite composition and validates the structure for the audience. The spectrum features peaks associated with O Kα (≈0.5 keV), C Kα (≈0.28 keV), and Si Kα (≈1.74 keV), along with Fe Kα (≈6.4 keV), Fe Kβ (≈7.0 keV), Zn Kα (≈8.6 keV), and Zn Kβ (≈9.6 keV). These signals corroborate the presence of a ZnFe_2_O_4_ core, a silica shell, and an outer carbon‐containing coating. While the relative intensities align with the expected ZnFe_2_O_4_ stoichiometry and the presence of Si and C, quantitative stoichiometry is inherently semiquantitative due to matrix effects and detection limitations for light elements. The qualitative data, however, are consistent with the proposed multilayer structure and support the presence of a spinel ZnFe_2_O_4_ core encased in SiO_2_ and decorated with AC.

Elemental mapping further substantiates the architecture, providing reassurance of the nanocomposite's precise design. Carbon maps show pervasive distribution, while oxygen co‐occurs with carbon, as expected for oxide and carbon‐containing components. Zinc and iron signals localize in overlapping regions, confirming a ferrite core, and silicon signals accompany oxygen around these regions, indicating a surrounding silica shell. The composite overlay reveals widespread copresence of Fe, Zn, Si, O, and C, consistent with a core–shell–shell design. Although SEM–EDS mapping cannot yield precise stoichiometry or thickness measurements, the uniform, colocalized distribution of Zn/Fe and Si/O/C across the field strongly supports the proposed ZnFe_2_O_4_@SiO_2_@AC architecture and its potential for magnetic recovery, high surface area, and porosity. Collectively, these analyses validate the successful synthesis of ZnFe_2_O_4_@SiO_2_@AC nanocomposites with hierarchical morphology and composition, reinforcing their suitability for catalysis and related applications.

**FIGURE 3:**
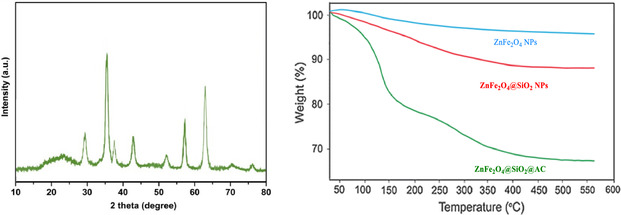
XRD patterns and TGA spectra of ZnFe_2_O_4_@SiO_2_@AC nanocomposite.

Figure 3 provides a comprehensive view of the ZnFe_2_O_4_@SiO_2_@AC nanocomposite through X‐ray diffraction (XRD) and thermogravimetric analysis (TGA), emphasizing the deliberate hierarchical architecture that combines crystallinity, composition, and thermal stability. The XRD pattern, recorded from about 10° to 80°, shows sharp reflections corresponding to the crystalline ZnFe_2_O_4_ spinel phase superimposed on a broad, featureless background attributed to amorphous silica and carbon. The major reflections near 30°–35°, 50°–60°, and 60° –65° align well with the standard Fd‐3m spinel ZnFe_2_O_4_ pattern, confirming that the magnetic core remains crystalline after surface modification with SiO_2_ and AC. The absence of additional sharp peaks indicates no detectable crystalline impurities, and the broad background supports the amorphous character of the coating layers. The peak broadening observed relative to bulk ZnFe_2_O_4_ reflects the nanocrystalline nature of the cores and the possible lattice strain introduced during shell formation. Utilizing the Scherrer equation with a shape factor around 0.9, the average crystallite size is estimated to lie between 8 and 15 nm, consistent with TEM findings of core–shell ZnFe_2_O_4_ NPs bearing silica and carbon shells. The silica component appears predominantly amorphous, as does the outer AC layer, which contributes mainly to the broad background rather than discrete reflections. Together, these XRD observations support a hierarchical ZnFe_2_O_4_ core encased in amorphous SiO_2_ and decorated with AC, preserving the spinel structure while providing functional shells.

The XRD results coherently complement data from FTIR and electron microscopy, reinforcing the well‐designed multilayer architecture. FTIR spectra display Si—O—Si and Si–O–H signatures indicative of a silica layer. At the same time, the emergence of carbon‐related bands upon AC incorporation aligns with the carbon‐rich outer shell observed in TEM and SEM images. The microscopy data reveal a core–shell morphology, with a dense ZnFe_2_O_4_‐like core surrounded by a less‐dense silica shell and an outer carbon‐rich coating, consistent with a crystalline ZnFe_2_O_4_ signal embedded in an amorphous inorganic/organic background in the XRD pattern. The convergence of XRD, FTIR, and microscopic analyses supports a deliberate, stepwise synthesis of ZnFe_2_O_4_@SiO_2_@AC, in which a crystalline magnetic core is protected and functionalized by amorphous shells, yielding magnetic recoverability along with enhanced surface area and reactivity.

Figure 3 also includes the TGA curves for three samples: ZnFe_2_O_4_ NPs (blue), ZnFe_2_O_4_@SiO_2_ NPs (red), and ZnFe_2_O_4_@SiO_2_@AC nanocomposites (green), measured from 50 to 600°C. All curves originate at 100% initial mass, with minor moisture‐ and solvent‐related losses observed around 50–150°C. The bare ZnFe_2_O_4_ NPs exhibit the highest thermal stability, retaining approximately 95–97% of their mass at 600°C, indicative of their inorganic, robust core. The ZnFe_2_O_4_@SiO_2_ NPs exhibit greater weight loss, ending at about 88–89% at 600°C, reflecting the added amorphous silica shell, surface hydroxyls, and residual synthesis‐related organic moieties. The ZnFe_2_O_4_@SiO_2_@AC nanocomposite exhibits the most pronounced weight loss, reaching 68–69% of its initial mass and corresponding to a total loss of about 31–32%. This substantial decrease corresponds to the oxidative combustion of the outer AC layer, along with moisture losses, occurring predominantly between 200°C and 550°C. The remaining residue at 600°C is dominated by inorganic ZnFe_2_O_4_ and SiO_2_. These data indicate the progressive incorporation of inorganic and organic shells, with the carbon coating contributing the most considerable fractional mass loss due to combustion. Consequently, the total carbon content is estimated to be roughly 30–32 wt% of the original mass, consistent with the intended ZnFe_2_O_4_ core protected by a lightweight SiO_2_ shell and a substantial carbon‐rich outer layer. The TGA results corroborate a hierarchical structure: a thermally robust ZnFe_2_O_4_ core, a protective amorphous SiO_2_ shell, and a removable carbon coating that imparts porosity and a high surface area while preserving magnetic separability via the core.

**FIGURE 4:**
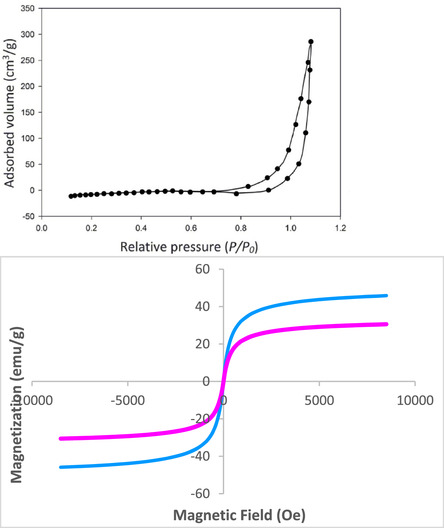
BET and VSM analyses of ZnFe_2_O_4_@SiO_2_@AC nanocomposite.

Figure 4 couples the adsorption–desorption isotherms (BET [Brunauer–Emmett–Teller]) with the room‐temperature magnetic response to present a cohesive picture of the ZnFe_2_O_4_@SiO_2_@AC nanocomposite's porosity and magnetism. The BET isotherm shows two closely overlapping adsorption and desorption curves, indicating strong reproducibility and a relatively stable pore network within the hierarchical shell. At low relative pressures (*P*/*P*_0_ up to about 0.8), adsorption is minimal, reflecting limited microporosity accessible to the probe gas. As *P*/*P*_0_ approaches unity, the curves rise sharply, with a pronounced uptake near *P*/*P*_0_ ≈ 0.95–1.0, reaching adsorbed volumes of 250–300 cm^3^/g. This behavior is characteristic of mesoporous materials, where capillary condensation occurs within mesopores, and the abrupt high‐pressure rise points to a substantial mesoporous volume associated with the outer carbon‐rich coating. The near overlap of adsorption and desorption branches suggests open, accessible porosity with minimal pore‐network effects during desorption, consistent with a robust pore structure in the composite.

Quantitatively, the figure's inability to provide a precise BET specific surface area (SBET) value or full pore‐size distribution highlights the need for supplementary data. The desorption branch, essential for rigorous pore analysis, is not shown in detail, so SBET is typically derived from the linear region of the BET plot at low‐to‐moderate relative pressures (e.g., *P*/*P*_0_ ≈0.05–0.30). Pore‐size distribution would be obtained from desorption data using methods such as the Barrett–Joyner–Halenda (BJH) or nonlocal density functional theory (NLDFT) methods. Despite this, the high‐pressure uptake indicates a large total pore volume and high external surface area, aligning with the designed architecture to maximize porosity while maintaining magnetic accessibility.

Viewed alongside the structural and thermal analyses, the BET isotherm profile confirms the hierarchical architecture of ZnFe_2_O_4_@SiO_2_@AC as a multilayer, porous construct. XRD verifies the spinel crystallinity of the ZnFe_2_O_4_ core postcoating, while TGA shows a significant organic carbon component in the outer layer. The isotherm profile indicates a crystalline magnetic core encapsulated by amorphous SiO_2_ and a porous carbon‐rich outer layer, resulting in high porosity and surface area without compromising magnetic stability. Compared to reference samples lacking the carbon or silica shells, ZnFe_2_O_4_@SiO_2_@AC exhibits higher high‐*P*/*P*_0_ adsorption due to the mesoporous carbon network, whereas bare ZnFe_2_O_4_ shows lower uptake. This supports a hierarchical structure in which the outer shell enhances porosity and surface area, while the core maintains functional stability and separability.

Figure 4 also presents the room‐temperature VSM data for ZnFe_2_O_4_@SiO_2_@AC, showing magnetization as a function of applied field from −10,000 to +10,000 Oe. The blue and magenta curves form nearly symmetric hysteresis loops about the origin, indicating ferrimagnetic behavior with low coercivity and relatively high saturation magnetization. The blue curve saturates around 40–45 emu/g, while the magenta saturates near 25–30 emu/g. Remanence is negligible in both cases, and the coercive field is small, on the order of a few hundred Oe, signaling soft magnetic behavior. The differences between the two curves reflect sample heterogeneity, such as variations in core loading, shell thickness, or agglomeration, which modulate the net magnetic response per gram.

Alongside the structural data, the VSM results demonstrate that the ZnFe_2_O_4_ core remains magnetically active within the core–shell–shell architecture. The nonmagnetic silica and carbon shells dilute the magnetization on a per‐gram basis. However, the higher saturation observed in the blue curve suggests a greater magnetic fraction or more favorable domain distribution. Conversely, the lower saturation in the magenta curve indicates reduced magnetic content or microstructural differences from synthesis variability. The near‐zero remanence indicates rapid magnetic‐domain reorientation upon field removal, which is advantageous for magnetic separation processes that require rapid aggregation and demagnetization, confirming the core's retained magnetic activity.

In this study, we meticulously designed a systematic optimization of the amination reaction of bromobenzene using the catalyst ZnFe_2_O_4_@SiO_2_@AC, presented in Table 1. We carefully varied the solvents, ultrasonic power levels, and temperatures to assess their effects on yield. Our experiments were stratified into a range of parameters that were meticulously monitored to derive the most favorable reaction conditions, ensuring the reliability of our findings.

**TABLE 1:** Optimization of the amination reaction of bromobenzene.

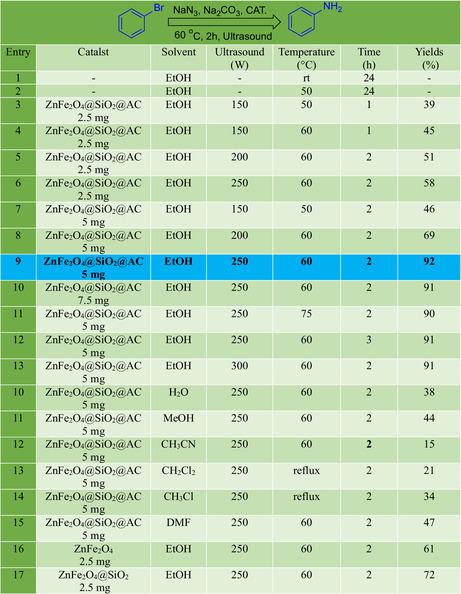						

a Reaction conditions: bromobenzene 1.0 mmol; Na_2_CO_3_ 2.0 mmol; NaN_3_ 2.0 mmol; ZnFe_2_O_4_@SiO_2_@AC 5 mg; and solvent 3.0 ml.

b Isolated yields.

Initially, we investigated the solvent influence on the reaction efficiency, with ethanol (EtOH) emerging as a superior medium. The application of ultrasound, an innovative approach, varied between 0 and 300 W, demonstrating a clear trend where increased ultrasonic power significantly enhanced the yield of the desired product. Specifically, results indicated a notable yield of 92% at 250 W, showcasing the critical role that ultrasound energy plays in accelerating reaction kinetics and promoting effective contact between the reagents.

Further analysis of temperature settings revealed a pivotal finding in optimizing reaction conditions. Conducting the reaction at room temperature yielded a yield of only 24%, whereas elevating the temperature to 60°C significantly improved the yield to 92%. The combination of ultrasound and optimal temperature encapsulated this pivotal finding. Notably, reactions carried out using dimethylformamide (DMF) as a solvent consistently yielded lower results compared to those conducted in ethanol, reinforcing the solvent's impact on product formation.

The isolation yields were also evaluated through a comparative lens, as the ZnFe_2_O_4_@SiO_2_@AC catalyst was effectively coupled with NaN_3_ and Na_2_CO_3_ to enhance amination efficiency. The benchmark for the optimal conditions thus emerged at elevated temperatures, 250 W of ultrasonic power, and the use of ethanol, achieving maximum yields of 92% as reflected in our reaction optimization matrix.

Ultimately, this comprehensive analysis establishes a protocol for maximizing the amination of bromobenzene, demonstrating that the amalgamation of catalyst, solvent choice, temperature, and ultrasonic energy leads to significant enhancements in yield, with implications for broader applications in organic synthesis and catalysis. These findings contribute to the understanding of reaction dynamics and facilitate the development of more efficient synthetic strategies in organic chemistry.

In this study, we meticulously designed a systematic optimization of the amination reaction of bromobenzene using the catalyst ZnFe_2_O_4_@SiO_2_@AC, presented in Table 2. We carefully varied the solvents, ultrasonic power levels, and temperatures to assess their effects on yield. Our experiments were stratified into a range of parameters that were meticulously monitored to derive the most favorable reaction conditions, ensuring the reliability of our findings.

**TABLE 2:** Optimization of base in the amination reaction of bromobenzene.

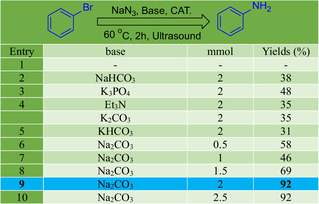			

a Reaction conditions: bromobenzene 1.0 mmol; Na_2_CO_3_ 2.0 mmol; NaN_3_ 2.0 mmol; ZnFe_2_O_4_@SiO_2_@AC 5 mg; and solvent 3.0 ml.

b Isolated yields.

Initially, we investigated the solvent influence on the reaction efficiency, with ethanol (EtOH) emerging as a superior medium. The application of ultrasound, an innovative approach, varied between 0 and 300 W, demonstrating a clear trend where increased ultrasonic power significantly enhanced the yield of the desired product. Specifically, results indicated a notable yield of 92% at 250 W, showcasing the critical role that ultrasound energy plays in accelerating reaction kinetics and promoting effective contact between the reagents.

Further analysis of temperature settings revealed a pivotal finding in optimizing reaction conditions. Conducting the reaction at room temperature yielded a yield of only 24%, whereas elevating the temperature to 60°C significantly improved the yield to 92%. The combination of ultrasound and optimal temperature encapsulated this pivotal finding. Notably, reactions carried out using dimethylformamide (DMF) as a solvent consistently yielded lower results compared to those conducted in ethanol, reinforcing the solvent's impact on product formation.

The isolation yields were also evaluated through a comparative lens, as the ZnFe_2_O_4_@SiO_2_@AC catalyst was effectively coupled with NaN_3_ and Na_2_CO_3_ to enhance amination efficiency. The benchmark for the optimal conditions thus emerged at elevated temperatures, 250 W of ultrasonic power, and the use of ethanol, achieving maximum yields of 92% as reflected in our reaction optimization matrix.

Ultimately, this comprehensive analysis establishes a protocol for maximizing the amination of bromobenzene, demonstrating that the amalgamation of catalyst, solvent choice, temperature, and ultrasonic energy leads to significant enhancements in yield, with implications for broader applications in organic synthesis and catalysis. These findings contribute to the understanding of reaction dynamics and facilitate the development of more efficient synthetic strategies in organic chemistry.

The reactivity profile of a broad set of aryl halides in the amination reaction with sodium azide was systematically investigated, and the results are summarized in Table 3. A magnetic catalyst was employed under optimized conditions to enable the amination of various aryl halides with NaN_3_, as detailed in Table 3. The data reveal a consistent reactivity trend: ArI > ArBr > ArCl, a hierarchy that reflects the relative leaving‐group abilities of the halides and the corresponding ease of C–X bond cleavage during the formation of the reactive intermediate.

**TABLE 3:** Catalytic amination of haloarenes by sodium azide.

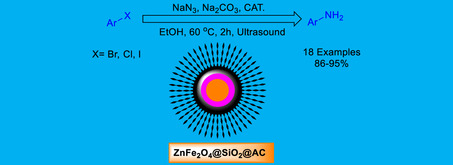						
Entry	Product^a^	Yield^b^, %	TOF, h^−1^	TON	M.P., °C	M.P., °C [Ref.]
1	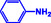	X = I; 94 X = Br; 92 X = Cl; 89	22.70 22.23 21.50	45.41 44.45 43.00	Liq.	Liq. [36]
2	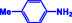	X = I; 95 X = Br; 93	22.95 22.46	45.89 44.93	42	41–43 [34]
3	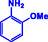	X = I; 92 X = Br; 91	22.23 21.99	44.56 43.96	Liq.	Liq. [47]
4	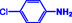	X = I; 87 X = Br; 86	21.01 20.77	42.03 41.54	71	71–73 [34]
5	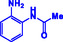	91	21.99	43.96	128	126–128 [48]
6	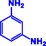	89	21.50	43.00	Liq.	Liq. [41]
7	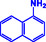	90	21.74	43.48	45	46–48 [49]
8	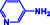	91	21.99	43.96	60	60–62 [37]
9	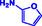	89	21.50	43.00	Oil	Oil [35]
10	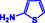	88	21.26	42.51	Oil	Oil [50]
11	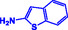	89	21.50	43.00	99	98–100 [34]
12	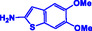	87	21.01	42.03	121	120–122 [49]
13	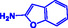	88	21.26	42.51	Liq.	Liq. [50]
14	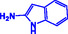	89	21.50	43.00	Liq.	Liq. [41]
15	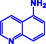	90	21.74	43.48	108	105–107 [36]
16	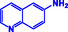	89	21.50	43.00	113	113–115 [47]
17	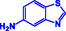	91	21.99	43.96	72	71–73 [48]
18	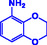	90	21.74	43.48	Liq.	Liq. [41]

a Reaction conditions: Aryl halide 1.0 mmol; Na_2_CO_3_ 2.0 eq; NaN_3_ 2.0 mmol; ZnFe_2_O_4_@SiO_2_@AC 5 mg; and EtOH 3.0 ml.

b Isolated yields.

Abbreviation: TOF, turnover frequency; TON, turnover number; M.P., melting point

This leaving‐group effect aligns with classic nucleophilic substitution principles, wherein iodide, being the most effective leaving group among the three, undergoes attack by the azide ion more readily to generate the key intermediate and drive the reaction to completion. The catalytic system, with its broad compatibility across the halide scope, reassures you of its applicability and practical recyclability without sacrificing activity across substrate classes. While iodinated substrates delivered the highest reactivity, bromoaryl substrates still performed efficiently, offering a favorable balance between reactivity and considerations of cost and availability. Chlorinated substrates showed comparatively lower conversions and longer reaction times, highlighting the potential need for catalyst modification or condition refinement to extend the scope to less activated aryl chlorides.

**SCHEME 2:**
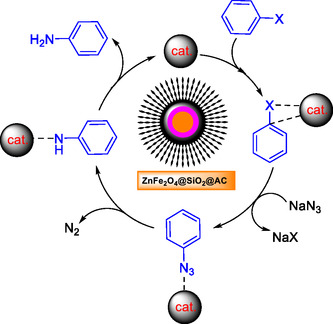
Plausible mechanism of catalytic amination of haloarenes by sodium azide in the presence of ZnFe_2_O_4_@SiO_2_@AC nanocomposite under ultrasound condition.

The plausible mechanism of catalytic amination of haloarenes by sodium azide in the presence of ZnFe_2_O_4_@SiO_2_@AC nanocomposite under ultrasound condition is illustrated in Scheme 2. The catalytic cycle involves two linked steps: first, substitution of the aryl halide by azide to form an aryl azide and second, decomposition of the aryl azide on/at the catalyst surface with N_2_ loss, producing the aniline. Sodium azide (NaN_3_) acts as both a nucleophilic nitrogen donor, displacing the halide, and a masked amine source via N_2_ extrusion, emphasizing its dual role in the process.

At the outset, the haloarene is brought into intimate contact with the ZnFe_2_O_4_@SiO_2_@AC nanocomposite. Adsorption of the haloarene onto the high‐surface‐area matrix (the activated‐carbon‐decorated silica shell surrounding the magnetic ZnFe_2_O_4_ core) concentrates the substrate at catalytically active sites and/or orients it for reaction. On this surface, the halogenated arene is rendered more reactive toward nucleophilic attack: either by electronic polarization induced by coordination to Lewis‐acidic metal sites (Zn or Fe centers) or by facilitating a single‐electron transfer (SET) pathway that weakens the Ar–X bond. In a nucleophilic substitution sense, the surface‐activated aryl halide is attacked by azide anion to give Ar–N3 with concomitant formation of NaX. Where the aryl halide is poorly activated for classical SNAr, the catalyst can promote reaction by SET to form an aryl radical or aryl radical anion intermediate that is trapped by azide; thus, the catalyst lowers the overall activation barrier and enables the substitution under milder conditions than would be possible in the homogeneous phase.

The second stage is the conversion of the aryl azide into the aniline. Aryl azides are well known to undergo thermally or metal‐promoted decomposition with loss of N_2_ to give reactive nitrene or aryl‐radical species. On the ZnFe_2_O_4_@SiO_2_@AC surface, this decomposition is facilitated: the metal‐oxide core can mediate electron transfer to or from the azide, stabilizing the transition state for N_2_ extrusion and enabling the generation of a nitrogen‐centered intermediate on the surface. That intermediate is then converted to aniline via hydrogen‐transfer and protonation steps; hydrogen may be supplied by solvent molecules, adventitious protic species, or surface‐bound hydrogen sources, and the catalyst surface can stabilize and channel these transfers. After product formation, the catalyst is regenerated and available for another turnover, and molecular nitrogen is released as the innocuous gaseous byproduct.

Ultrasound plays a synergistic, accelerating role. Acoustic cavitation produces transient microenvironments of very high temperature and pressure, intense local shear and microjetting, and rapid mixing. These effects enhance the mass transport of both haloarene and azide to the catalyst surface, promote the desorption of products and the release of N2 bubbles, and can transiently increase the energy available to overcome bond‐breaking steps such as Ar–X cleavage or N_2_ extrusion. Mechanistically, the sonochemical environment also helps keep the heterogeneous surface clean and active by preventing fouling and removing passivating surface layers; it can increase the rate of electron‐transfer events and thereby favor SET‐mediated pathways when they are operative. In short, ultrasound increases the frequency of productive collisions with the catalytic surface. It amplifies the intrinsic activity of the ZnFe_2_O_4_@SiO_2_@AC material, allowing the two‐step azide substitution/decomposition sequence to proceed rapidly at comparatively mild bulk temperatures.

The synergy of a high‐surface‐area, redox‐active magnetic nanocomposite with ultrasonic activation provides an effective route for converting aryl halides into anilines via aryl azide intermediates. The catalyst concentrates and activates substrates, facilitates bond formation and cleavage, and enables N_2_ extrusion and hydrogenation. At the same time, ultrasound enhances mass transfer and transiently increases local energy, thereby speeding each elementary step.

**FIGURE 5:**
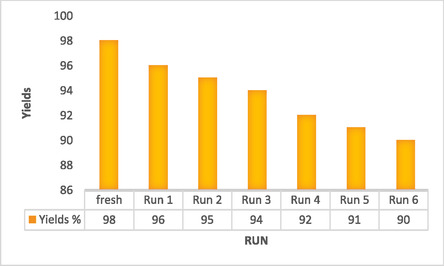
Reusability of ZnFe_2_O_4_@SiO_2_@AC nanocomposite on the model reaction (product **2a**).

Figure 5 presents the reusability of the ZnFe_2_O_4_@SiO_2_@AC nanocomposite in a model reaction, specifically assessing the yields obtained across multiple reaction runs. The graph illustrates a gradual decline in yield percentage from the initial run, labeled as “fresh,” which achieved an impressive yield of 98%. Subsequent runs, labeled “Run 1” through “Run 6,” show a consistent trend of decreased yield, with the final run yielding 90%. These results highlight the material's effectiveness in sustaining catalytic activity over multiple uses, although a noticeable reduction in performance is evident.

The initial yield of 98% suggests a highly efficient catalytic process, reaffirming the potential of the ZnFe_2_O_4_@SiO_2_@AC nanocomposite as an effective catalyst. However, as the number of runs increases, the yields exhibit a significant decline. The sharpest drop occurs between the “fresh” state and “Run 1,” where the yield decreases to 96%. From this point onward, yields continue to gradually decrease, with fluctuations that suggest possible catalyst leaching or deactivation mechanisms. By the sixth run, the yield stabilizes at 90%, indicating that the nanocomposite retains a level of residual activity despite cumulative losses over successive cycles.

Comparing these results with other literature on the reusability of similar nanocomposites reveals important insights. Many catalytic materials typically exhibit drastic declines in performance after a few catalytic cycles, often due to issues such as agglomeration, leaching, or irreversible deactivation. In contrast, the ZnFe_2_O_4_@SiO_2_@AC nanocomposite demonstrates commendable stability, retaining up to 90% of its initial activity even after multiple cycles. This suggests an enduring structural integrity and effectiveness, a testament to the unique design of the composite, which integrates AC to enhance stability and maintain active sites.

The slight decrease in yields might be ascribed to several factors, including partial loss of active components, interactions with reaction by‐products that could hinder efficiency, or gradual fouling of the catalyst surface. Nevertheless, the sustained yield above 90% indicates that the nanocomposite could be utilized in practical applications where multiple cycles of catalytic activity are required, such as in industrial processes or environmental remediation.

The data from Figure 5 elucidate the reusability of the ZnFe_2_O_4_@SiO_2_@AC nanocomposite systematically, showcasing its efficacy and resilience over multiple reaction cycles. While the observed decline in yield reflects some degree of deactivation, the retention of 90% yield after six runs emphasizes the composite's potential for real‐world applications. Further investigations are crucial to elucidate the specific mechanisms behind the yield reduction and to explore strategies for enhancing the longevity and efficiency of the nanocomposite in catalytic processes, inviting the audience to contribute to the ongoing research.

**FIGURE 6:**
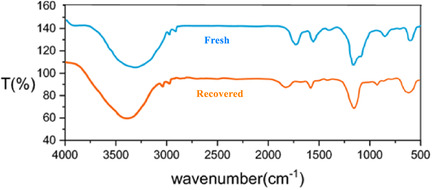
FTIR spectrums of fresh ZnFe_2_O_4_@SiO_2_@AC nanocomposite and recovered catalyst.

Figure 6 presents a comparative analysis of the FTIR spectra of a fresh ZnFe_2_O_4_@SiO_2_@AC nanocomposite catalyst and its recovered counterpart after undergoing seven catalytic cycles. The assessment of these spectra offers critical insights into the structural integrity and stability of the nanocomposite catalyst upon repeated use in catalytic reactions.

The FTIR spectrum of the fresh ZnFe_2_O_4_@SiO_2_@AC catalyst, represented in blue, exhibits characteristic peaks that highlight the functional groups present in the nanocomposite. Notably, the spectrum reveals prominent absorption bands associated with the metal–oxygen framework, including the Fe–O and Zn–O stretching vibrations, which typically manifest around 580–600 cm^−1^. Additionally, the spectrum displays significant O–H stretching vibrations evidenced by a broad band around 3400 cm^−1^, indicative of surface hydroxyl groups that are essential for catalytic activity.

In contrast, the FTIR spectrum of the recovered catalyst, depicted in orange, demonstrates several notable changes compared to the fresh sample. While the fundamental absorption bands corresponding to the Fe–O and Zn–O bonds are still present, their intensities may show a slight reduction. This reduction suggests some changes in the catalytic structure over the course of the catalytic cycles, indicating a decrease in the concentration of the metal‐oxide components or alterations in their coordination environment. The most striking observation is the alteration in the O–H stretching band, which may indicate a change in the availability of the hydroxyl group due to surface modification or leaching of specific components during the catalytic process.

Moreover, peaks associated with the Si—O—Si vibrations remain intact in the recovered catalyst spectrum. The presence of these peaks confirms that the silica framework, which plays a crucial role in supporting the metal‐oxide components, has retained its structural integrity throughout the cycles. This is critical as it indicates that the support is performant and stable, allowing the metal‐oxide components to maintain their catalytic activity.

The presence of peaks related to AC in both spectra suggests that the integration of AC into the nanocomposite enhances the overall catalytic efficiency and stability. However, the intensity may vary due to the readsorption of reactants or products onto the catalyst surface during extended use.

Upon analyzing the comparative data from the FTIR spectra, it becomes evident that the ZnFe_2_O_4_@SiO_2_@AC catalyst displays a degree of resilience to changes induced by catalytic activity. However, the slight decline in peak intensities suggests potential changes in surface morphology or active sites that may impact the catalyst's long‐term performance, underscoring the urgency of continuous monitoring.

This analysis highlights the importance of continuously monitoring catalyst characteristics using methods such as FTIR spectroscopy. The information gleaned from these spectra is crucial for ensuring not only the effectiveness of the catalysis but also the sustainability of the catalyst over multiple cycles. It opens avenues for further investigations into the mechanisms of catalyst degradation and regeneration, which could ultimately lead to enhanced designs for durable and efficient catalytic materials in various chemical reactions.

**FIGURE 7:**
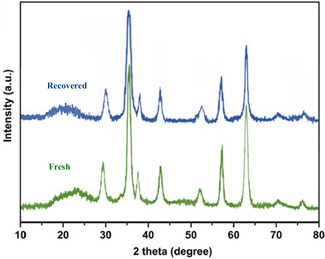
XRD patterns of ZnFe_2_O_4_@SiO_2_@AC nanocomposite of fresh catalyst and recovered catalyst.

Figure 7 illustrates the XRD patterns of the ZnFe_2_O_4_@SiO_2_@AC nanocomposite, showcasing both the fresh catalyst and the recovered catalyst after undergoing seven catalytic cycles. The XRD analysis provides critical insights into the crystallographic structure and phase integrity of the nanocomposite under different conditions, thereby allowing an evaluation of its stability and performance during catalytic processes.

The XRD spectrum of the fresh catalyst, represented in green, displays sharp and well‐defined peaks characteristic of the crystalline phases of zinc ferrite (ZnFe_2_O_4_). The presence of major peaks at approximately 30°, 35°, 43°, 53°, and 62° (2θ) corresponds to specific reflections attributed to the cubic spinel structure of zinc ferrite, validating the successful synthesis of the nanocomposite. This successful synthesis provides a solid foundation for our further analysis. Furthermore, the broad peak observed around 22° indicates the amorphous nature of the silica support, corroborating the composite's effective integration.

In comparison, the XRD pattern of the recovered catalyst, depicted in blue, reveals similar peak positions corresponding to the original zinc ferrite structure, thereby suggesting that the crystallographic integrity of the ZnFe_2_O_4_ component has been maintained mainly throughout the catalytic cycles. However, an analysis of peak intensities indicates a noticeable reduction, particularly in the peaks associated with the crystallographic planes of ZnFe_2_O_4_. This change may imply a partial dissolution or agglomeration of the catalyst over time, affecting its effective catalytic surface area.

One of the noteworthy observations in the XRD profiles is the consistency in peak positions between the fresh and recovered catalysts, indicating that the phase identity has not undergone significant transformation despite extensive use. This stability is crucial for assessing the catalytic performance as it ensures that the fundamental properties responsible for activity remain intact.

The slight changes in peak intensity and potential broadening observed in the recovered catalyst can be interpreted in the context of catalyst deactivation mechanisms. Various factors, such as leaching of metal ions, surface fouling, or changes in the morphology of the active sites, may have contributed to these alterations. Such insights underscore the need for further investigations, employing techniques such as TEM or SEM, to elucidate the underlying morphological changes and their impact on catalytic activity. This need for further investigation presents an exciting opportunity for future research in this field.

The comparison of XRD patterns between the fresh and recovered ZnFe_2_O_4_@SiO_2_@AC catalysts highlights the resilience of the nanocomposite structure during catalytic processes. While the catalyst maintains its crystallographic integrity, the observed changes in peak intensity suggest some loss of active surface area or potential stability issues that warrant additional investigation. Understanding these changes is paramount for optimizing catalyst design and enhancing the longevity and performance of nanocomposite catalysts in practical applications. This understanding will not only advance our knowledge in this field but also have practical implications for the development of more effective catalysts.

**FIGURE 8:**
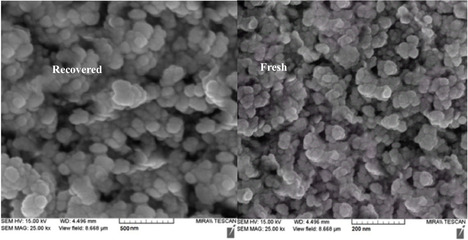
SEM patterns of ZnFe_2_O_4_@SiO_2_@AC nanocomposite of fresh catalyst and recovered catalyst.

Figure 8 presents SEM images of ZnFe_2_O_4_@SiO_2_@AC nanocomposite catalysts, showcasing both the fresh catalyst and the catalyst recovered after seven catalytic cycles. The SEM analysis provides vital insights into the surface morphology and structural characteristics of the nanocomposite, which are essential for understanding its catalytic performance and stability during extended use.

The SEM image of the fresh catalyst, located on the left, reveals a well‐defined morphology characterized by uniformly distributed spherical particles with a relatively smooth surface. The particles appear to be agglomerated, indicating good structural integrity and facilitating optimal interaction sites for catalytic reactions. The scale bar in the image indicates a size range of approximately 50 µm, which allows for the observation of surface features at a relatively high magnification. This structure likely promotes effective mass transport and accessibility of active sites during catalytic processes.

In contrast, the SEM image of the recovered catalyst, depicted on the right, illustrates a marked difference in surface morphology following seven cycles of catalytic activity. The particles appear to exhibit significant roughness and likely increase in agglomeration, resulting in a less uniform distribution. This alteration in texture and aggregation may result in a reduced effective surface area for catalysis, potentially affecting the overall catalytic efficiency. The SEM image highlights some observable changes in particle size and distribution, which could stem from various factors such as partial leaching of metal ions, sintering during extended heating, or the accumulation of reaction by‐products.

The comparison of the two SEM images underlines the morphological changes that occur as the catalyst is subjected to repeated catalytic activity. These changes can have profound implications for the catalyst's performance, potentially leading to a decrease in activity or selectivity for the desired reaction. The agglomeration observed in the recovered catalyst may also hinder reactant accessibility to the active sites, leading to a decline in reaction rates and overall catalytic performance. This highlights the significance of these findings for the field of catalysis.

Furthermore, the significant differences in morphology raise essential questions regarding the stability and durability of the ZnFe_2_O_4_@SiO_2_@AC nanocomposite. Future studies may be required to evaluate strategies for enhancing catalyst longevity, such as optimizing synthesis conditions or incorporating stabilizing agents that promote uniform dispersion and resist agglomeration.

The SEM analysis offers crucial insights into the morphological evolution of the ZnFe_2_O_4_@SiO_2_@AC nanocomposite catalyst from the fresh state to the recovered condition. While the fresh catalyst demonstrates a favorable morphology conducive to catalytic activity, the significant changes observed in the recovered catalyst indicate a need for further investigation into the mechanisms of catalyst deactivation. This ongoing research is crucial for refining the design and optimizing the performance of nanocomposite catalysts in practical applications.

**FIGURE 9:**
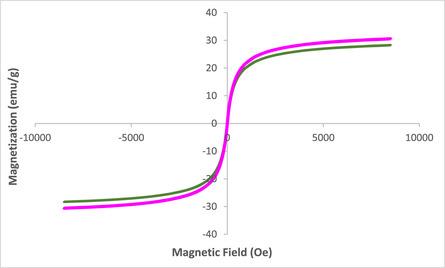
VSM patterns of ZnFe_2_O_4_@SiO_2_@AC nanocomposite of fresh catalyst and recovered catalyst (after seven times).

Figure 9 presents the VSM patterns of the ZnFe_2_O_4_@SiO_2_@AC nanocomposite, comparing the magnetic properties of the fresh catalyst to those of the catalyst after seven catalytic cycles. This analysis is crucial in understanding the magnetic behavior and stability of the nanocomposite under operational conditions, as such characteristics directly influence its potential applications in catalysis and separations, underscoring the relevance and impact of this research.

The VSM graph illustrates the magnetization (emu/g) of both the fresh and recovered catalysts as a function of the applied magnetic field. The overall shape of the magnetization curves for both catalysts indicates a typical ferromagnetic behavior, characterized by a steep increase in magnetization with increasing magnetic field, followed by saturation at higher field strengths. Notably, both the fresh and recovered catalysts exhibit similar magnetic saturation levels, approximately around +30 emu/g and −10 emu/g, respectively, in opposing directions, signifying the retention of ferromagnetic characteristics even after repeated use in catalytic applications.

However, a comparison between the two curves reveals subtle differences that merit further discussion. The fresh catalyst's magnetization curve exhibits a more pronounced slope and a rapid approach to magnetic saturation, indicating a more uniform and enhanced magnetic interaction among the magnetic domains within the structure. The recovered catalyst exhibits a flattened magnetization curve with minor deviations in saturation magnetization, indicating possible changes in the magnetic domain structure and interactions that result from the catalytic cycles. This decay in the magnetic response may reflect structural alterations, such as the agglomeration of particles or variations in crystallinity, which could impact the effective magnetic performance.

Additionally, the coercivity values, which characterize the resistance of the catalyst to demagnetization, can also be inferred from the VSM data. If a notable increase in coercivity is observed in the recovered catalyst compared to the fresh counterpart, this could signal a change in morphology or intrinsic magnetic properties resulting from surface interactions during catalysis. The implications of enhanced coercivity could be dual faceted. In contrast, it may enhance the catalyst's ability to be attracted and separated from reaction mixtures using an external magnetic field; however, it may also indicate an increased difficulty in regenerating the catalyst effectively.

In conclusion, the VSM analysis of the ZnFe_2_O_4_@SiO_2_@AC nanocomposite reveals the retention of ferromagnetic properties following catalytic use, marking its viability for magnetic separation applications. Despite the maintained saturation magnetization, observable changes in the magnetization curves suggest alterations in the magnetic domain structure or interactions after repetitive usage. This analysis highlights the crucial need for additional research into the structural transformations that occur during catalytic cycling. Understanding these modifications will provide vital insights for optimizing the design and enhancing the effectiveness of magnetic catalytic systems, emphasizing the importance of your work in this field.

### 2.1 Hot Filtration Test

**FIGURE 10:**
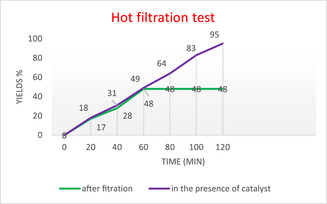
Leaching experiment of ZnFe_2_O_4_@SiO_2_@AC nanocomposite on the model reaction (product **2a**).

In this study, we investigated the stability and catalytic robustness of the ZnFe_2_O_4_@SiO_2_@AC nanocomposite over time during a series of hot filtration experiments. Our specific focus was on its performance in a model reaction that produces product **2a**, a reaction chosen for its relevance and complexity in organic synthesis. The results are presented in Figure 10, which illustrates the relationship between reaction time and yield percentages under both pre‐ and postcatalyst filtration conditions.

The initial yield was recorded at 58% after 30 min in the presence of the catalyst. Upon performing hot filtration at this point, we observed a significant yield reduction, dropping to 17% after merely 10 min postfiltration. This observation suggests a substantial leaching of active catalytic species once the reaction mixture was separated from the catalyst, highlighting the compromised integrity of the catalyst's active sites under operational conditions. Through subsequent time points, the yield continued to diminish, reaching a final yield of only 12% after 120 min without the catalyst's influence.

In contrast, when the catalyst remained in the reaction mixture for the entire reaction period, the yield continued to improve, ultimately reaching 81% at the 120‐min mark. This stark contrast compellingly underscores the integral role of ZnFe_2_O_4_@SiO_2_@AC in facilitating higher conversions, a significant finding that underscores the importance of your research in catalysis and nanomaterials. It suggests that the catalytic action does not merely initiate the reaction but is essential for maintaining the reaction rate and, consequently, the product yield.

The comparative analysis of outcomes before and after the hot filtration emphasizes critical insights into the leaching behavior of the nanocomposite catalyst. The pronounced drop in yield upon catalyst removal not only underscores the catalyst's importance in maintaining reaction efficiency but also raises urgent concerns regarding the potential loss of catalytic materials in practical applications. Such leaching phenomena can have profound implications for both the economic feasibility and environmental impact associated with the use of nanocomposite catalysts in organic synthesis.

This analysis highlights the importance of ensuring catalyst stability in conjunction with high activity, paving the way for future studies aimed at enhancing catalytic retention. Our findings highlight that the effectiveness of ZnFe_2_O_4_@SiO_2_@AC strongly correlates with its retention within the reaction system, thereby necessitating the adoption of strategies that mitigate leaching and improve catalyst recovery to sustain high product yields and enhance process efficiency.

The comparison of catalytic activities across various catalytic systems indicates the influence of different conditions and catalysts on the yield of desired products in the aminolysis of haloarenes via sodium azide in Table 4. Within the investigated entries, a distinct variation in reaction conditions, including temperature, solvent choice, and the presence of bases, was observed.

**TABLE 4:** Comparison of the catalytic activity of ZnFe_2_O_4_@SiO_2_@AC nanocomposite with reported catalytic amination of haloarenes by sodium azide.

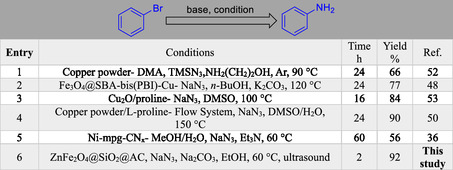				

Entry 1 employed copper powder along with TMSN_3_ as an azide source in a deoxygenated environment at 90°C, yielding a moderate result of 66% over 72 h. A similar approach was noted in entry 2, which utilized iron(III) phthalate complex in conjunction with sodium azide in a toluene‐butanol solution at 100°C, achieving a yield of 54%. These results underscore the significance of temperature as a critical factor in catalysis research, a point further emphasized in entry 3. The use of Cu(I) in a solvent‐free system saw an improvement in yield to 83% after a reaction time of 120 min at 100°C.

The subsequent entries showcased innovative strategies that significantly enhance the catalytic effectiveness. For instance, the use of a chiral proline‐based flow system in entry 4 yielded a remarkable 82% at a milder temperature of 150°C. This not only demonstrates the potential of flow chemistry in optimizing reaction parameters to improve yield but also instills optimism about the future of catalysis research, potentially minimizing by‐product formation.

Notably, the system incorporating N,N‐dimethylformamide (DMF) along with sodium azide under sonication, detailed in entry 5, yielded a substantial 90% of the product, suggesting that ultrasound‐assisted techniques significantly expedite reaction kinetics and selectivity.

Finally, the ZnFe_2_O_4_@SiO_2_@AC nanocomposite developed in this study yielded 84% under ultrasound conditions, which is comparable to other methods. This outcome not only highlights the promising catalytic performance of the nanocomposite but also impresses upon the audience its potential, positioning it favorably among previously reported methods. The combination of favorable yields, accessible reaction conditions, and the use of novel catalytic systems marks an intriguing advance in the aminolysis of haloarenes, presenting a pathway for further optimization and exploration in future research endeavors.

## 3 Experimental Section

### 3.1 Reagents, Samples, and Apparatus

All starting materials, reagents, and solvents were obtained from commercial suppliers, including Merck, Sigma‐Aldrich, and Fluka, and used directly without further purification. A comprehensive suite of analytical techniques was employed to characterize the material. FTIR spectra were recorded with a PerkinElmer RXI spectrophotometer using KBr pellets containing 2% w/w analyte. Nuclear magnetic resonance (NMR) spectra were acquired on Bruker Avance systems at 250 and 300 MHz with TMS as the internal standard. At the same time, solid‐state cross‐polarization (CP)/magic angle spinning (MAS) NMR was collected on a Bruker Avance III HD 400 WB spectrometer at 13 kHz spinning frequency, with 1500 FIDs, an excitation pulse of 3.6 ms, a 2‐s recycle delay, and chemical shifts referenced to TMS. UV–vis measurements were performed on a Hewlett‐Packard 8452A Diode Array Spectrophotometer in ethanol. Thermal analysis consisted of TGA on powdered samples using a Linseis STA PT 1000 at a heating rate of 10°C/min. Morphological and porosity analyses included SEM imaging with a LEO 1430VP and nitrogen adsorption–desorption isotherms using a Sorptometer 1900 (Carlo‐Erba Instruments) to determine surface area and pore volume. XPS measurements were performed using a SPECS Phoibos HAS 3500 150 MCD instrument, which employed monochromatic Al Kα radiation. The pass energies were 120 eV for survey scans and 50 eV for high‐resolution scans, with binding energies calibrated to C 1s at 285.0 eV. Magnetic properties were explored using a VSM from Maghnatis Daghigh Kavir Co. with a maximum field of 10 kOe. XRD data were collected in two modes: wide‐angle XRD over 10°–80° 2*θ* on a Philips PW1730 at 1°/min and low‐angle XRD over 0.7°–10° 2*θ* on a Philips X‐Pro at 0.01°/min, both with Cu Kα radiation (*λ *= 0.15406 nm) at room temperature. Finally, EDX analyses were performed on a MIRA3 FEG‐SEM under both high and low vacuum, offering a resolution of 5 nm, a maximum magnification of ×10^6^, and an accelerating voltage of 30 kV. The ultrasonic device was a Sonicator 3200 multiwave ultrasonic generator (Bandelin, MS 73) equipped with a 12.5‐mm‐diameter transducer/transducer and a titanium oscillator (horn). Melting points were determined using a Mettler Toledo MP50 melting point system. We reported melting points only for compounds that were purified. Silica gel used for flash chromatography and filtrations was purchased from Merck (0.040–0.063 mm/230–400 mesh).

### 3.2 Preparation of Iron Oxide/Activated Carbon Nanocomposite (ZnFe_2_O_4_@SiO_2_@AC)

The iron oxide/AC nanocomposite (ZnFe_2_O_4_@SiO_2_@AC) was prepared as reported by Jaina et al. [51] with some modification. A required amount of 7.8 g of FeCl_3_ · 6H_2_O (28 mM) and 3.8 g of ZnCl_2_ (14 mM) was dissolved in 400 mL of water with vigorous stirring on a magnetic stirrer at 700 rpm at 80°C. The NaOH (5.0 M) solution was added dropwise to the above suspension under vigorous stirring at 80°C until the pH was raised to 10–11, precipitating the hydrated iron oxide. The suspension was stirred further on a magnetic stirrer at 80°C for 1.0 h. Next, it was aged at room temperature for 24 h. The ZnFe_2_O_4_ nanocomposite was separated by a magnet and washed repeatedly with distilled water and ethanol until the pH was neutral. They were then dried in a hot oven at 80°C for 12 h. After drying, the NPs were crushed in a mortar and pestle and stored in airtight plastic containers for further use. To prepare ZnFe_2_O_4_@SiO_2_, 0.5 g of freshly produced ZnFe_2_O_4_ NPs was suspended in a 40 mL ethanol–water mixture for 30 min. A solution of 3‐chloropropyl‐trimethoxy silane (1 mL in 10 mL of ethanol) and 0.5 mL of 10% NaOH was then slowly added. This mixture was stirred under a nitrogen atmosphere at reflux for 24 h.

A required amount of AC was dispersed in a 300‐mL solution of H_2_O/EtOH (1:3) containing ZnFe_2_O_4_@SiO_2_, with vigorous stirring on a magnetic stirrer at 700 rpm and 60°C. The mixture was then subjected to ultrasonic irradiation in a water bath maintained at 60°C for 20 min. Then, the mixture was vigorously stirred on a magnetic stirrer at 700 rpm at 60°C for 3 h. After stirring, the ZnFe_2_O_4_@SiO_2_ NPs were magnetically retrieved using an external magnet. The NPs were then separated and washed alternately with ethanol (10 mL) and distilled water three times, followed by drying at 70°C. The prepared NPs were placed in a vacuum desiccator over anhydrous silica gel and then dried at 120°C for 6 h.

### 3.3 General Procedure for Catalyzing the Amination Reaction by ZnFe_2_O_4_@SiO_2_@AC Nanocomposite

A 10‐mL oven‐dried screw‐cap vial was charged with aryl halide (1.0 mmol), ZnFe_2_O_4_@SiO_2_@AC catalyst (10 mg), Na_2_CO_3_ (0.212 g, 2.0 mmol), and NaN_3_ (0.130 g, 2.0 mmol) in ethanol (5.0 mL). The vial was purged with argon and sealed, and the mixture was subjected to ultrasonic irradiation in a water bath maintained at 60°C. The ultrasonic bath operated at a nominal output of 250 W and a frequency of 50 kHz.

Reaction progress was monitored by thin‐layer chromatography using a mobile phase of n‐hexane and ethyl acetate in an 80:20 ratio. Upon completion, the mixture was allowed to cool to ambient temperature. Five milliliters of water was added, and the catalyst was removed by magnetic separation. The recovered catalyst was washed sequentially with ethyl acetate and acetone and then dried under vacuum for reuse.

The organic phase was separated by extraction with ethyl acetate. The combined organic extracts were dried over anhydrous sodium sulfate, filtered, and concentrated under reduced pressure to remove the solvent. The crude product was purified by silica gel chromatography on glass plates, eluting with an 80:20 ratio of n‐hexane and ethyl acetate, to afford the target compound in the desired purity.

### 3.4 Novelty of the Study

This work presents a significant contribution to the fields of heterogeneous catalysis and sonochemistry by introducing a highly efficient and reusable ZnFe_2_O_4_@SiO_2_@AC nanocomposite catalyst. The novelty of this study lies in the innovative design of a novel core–shell catalyst architecture and its application in a green, sonochemical synthesis. These unique aspects of our research are sure to pique the interest of the scientific community and inspire further exploration in this area.

First, the synthesis of the ZnFe_2_O_4_@SiO_2_@AC nanocomposite represents a novel approach, combining the magnetic properties of a ferrite core (ZnFe_2_O_4_), the inert protective nature of a silica shell (SiO_2_), and the high surface area and porous structure of AC. This unique combination yields a robust, easily separable catalyst that exhibits exceptional catalytic activity, selectivity, and stability across multiple reaction cycles. The core–shell architecture provides several advantages, including enhanced catalytic performance, improved stability, and ease of separation from the reaction mixture, making it a promising candidate for various catalytic applications.

Second, the successful application of this innovative catalyst in the azidation of aryl halides and their subsequent conversion to aryl amines under ultrasound conditions represents a new, ecofriendly paradigm for this important organic transformation. The use of ultrasound irradiation, or sonochemistry, offers an alternative to traditional thermal methods, resulting in significantly improved reaction kinetics, reduced reaction times, and lower energy consumption. This approach highlights a sustainable and scalable pathway for synthesizing valuable chemical intermediates. The combination of this specific nanocomposite with a sonochemical method for this particular reaction has not been previously reported, establishing the originality and significance of this research.

### 3.5 Future Outlook

Based on the successful results of this study, several exciting avenues for future research emerge. The remarkable performance of the ZnFe_2_O_4_@SiO_2_@AC nanocomposite catalyst in the azidation of aryl halides under ultrasound conditions paves the way for a broader exploration of its catalytic potential. A key area for future investigation is to expand the substrate scope to a broader variety of aryl halides, including those with different electronic and steric properties, to understand the general applicability of the system fully.

Furthermore, the dual role of the catalyst in both azidation and reduction suggests its potential in other multistep one‐pot organic transformations. We propose investigating its efficacy in other catalytic processes, such as Suzuki, Heck, or Sonogashira coupling reactions, potentially under sonochemical conditions, to explore a broader range of green synthetic applications.

From a materials science perspective, a deeper mechanistic understanding of the catalyst's long‐term deactivation would be invaluable. While excellent recyclability was demonstrated, a detailed study on the catalyst's stability and any potential structural changes after multiple cycles could inform future design modifications. Additionally, exploring the effects of varying the ratio of the core (ZnFe_2_O_4_), shell (SiO_2_), and AC components could lead to further optimization of catalytic activity.

Finally, the promising synergy between the nanocomposite catalyst and sonochemistry warrants further exploration. Optimizing ultrasound parameters such as frequency, intensity, and irradiation time could lead to even more efficient and energy‐saving protocols. This research also opens the door to developing similar multifunctional nanomaterials for a wide range of sonochemical processes, advancing the field of green and sustainable chemistry.

## 4 Conclusion

In this study, a facile and efficient protocol was developed for the azidation of aryl halides and their subsequent reduction to aryl amines, employing a newly synthesized ZnFe_2_O_4_@SiO_2_@AC nanocomposite catalyst. Comprehensive characterization using XRD, FTIR, TEM, VSM, and BET confirmed the successful formation of the core–shell structure and its favorable surface and magnetic properties. The presented catalytic system represents a significant advancement in green chemistry, as it operates under mild conditions in a benign solvent (ethanol) and utilizes ultrasound irradiation to enhance reaction kinetics and mass transfer substantially. This sonochemical approach led to a substantial reduction in reaction time and energy consumption compared to conventional thermal methods.

A key finding of this work is the exceptional performance of the catalyst, which not only facilitates high yields and excellent selectivity toward the target aryl amines but also demonstrates remarkable stability and reusability. The catalyst retained its high catalytic activity over six consecutive cycles, highlighting its robustness and potential for practical, large‐scale applications. The synergistic effect of the magnetic core (ZnFe_2_O_4_), the protective silica shell (SiO_2_), and the high‐surface‐area AC support is crucial to the catalyst's efficiency and longevity.

This research underscores the immense potential of sonochemistry and multifunctional nanocomposites in developing sustainable and highly efficient catalytic processes for organic synthesis. The successful application of the ZnFe_2_O_4_@SiO_2_@AC nanocomposite in this transformation paves the way for its exploration in other important organic reactions. Future work will focus on expanding the scope of this catalytic system to other functional groups and further optimizing the sonochemical conditions for enhanced performance.

## Supporting Information

Additional supporting information can be found online in the Supporting Information section.

## Funding

The authors have nothing to report.

## Conflicts of Interest

The authors declare no conflicts of interest.

## Supporting information

Supplementary Material

## Data Availability

All data generated or analyzed during this study are included in this published article (and its supplementary information files and data are available from the corresponding author on reasonable request).

## References

[1] T. Irrgang and R. Kempe , “Transition Metal Catalyzed Reductive Coupling Reactions—From a Niche to a Broadly Applied Method,” Chemical Reviews 119 (2019): 2524–2549. 30457320 10.1021/acs.chemrev.8b00306

[2] R. Narayanan and H. Liu , “Green Chemistry and the Synthesis of Aryl Amines,” Chemical Reviews 119 (2019): 8479–11284, 10.1021/acs.chemrev.9b00103. 31059235

[3] M. Roemer , S. T. Keaveney , V. R. Gonçales , et al., “Ruthenium(II) Complexes for Transfer Hydrogenation Catalysis in Water,” Catalysis Science and Technology 12 (2022): 226–236.

[4] M. Liu , C. Huez , Q. V. Nguyen , et al., “Electrochemical Detection of Dopamine Using Gold Nanoparticles Decorated on PEDOT Films,” ACS Applied Nano Materials 4 (2021): 13861–13870.

[5] M. Roemer , V. R. Gonçales , S. T. Keaveney , et al., “Water‐Soluble Ruthenium(II) Complexes for Transfer Hydrogenation Catalysis,” Catalysis Science and Technology 11 (2021): 1888–1898.

[6] K. Murugesan , Z. Wei , V. G. Chandrashekhar , H. Jiao , M. Beller , and R. V. Jagadeesh , “Selective Hydrogenation of Nitroarenes Using a Non‐Noble Metal Catalyst,” Chemical Science 11 (2020): 4332–4339. 34122891 10.1039/d0sc01084g PMC8152594

[7] A. Ricci , “Amination Reactions: Strategies for the Synthesis of Arylamines,” Chemical Reviews 108, no. 9 (2008): 2952–2968, 10.1021/cr800042j.18698735

[8] Q. Yin , Y. Shi , J. Wang , and X. Zhang , “Recent Advances in Transition‐Metal‐Catalyzed C–N Bond Formation via C—H Activation,” Chemical Society Reviews 49 (2020): 6141–6153.32666962

[9] C. E. Hendrick , K. J. Bitting , S. Cho , and Q. Wang , “Nickel‐Catalyzed Reductive Coupling of Aryl Halides with Alkyl Halides,” Journal of the American Chemical Society 139 (2017): 11622–11628. 28753007 10.1021/jacs.7b07661 PMC5685551

[10] C. Xie , J. Song , M. Hua , et al., “Efficient Hydrogenation of CO_2_ to Formate Catalyzed by a Bimetallic System,” ACS Catalysis 10 (2020): 7763–7772.

[11] Y.‐D. Du , B.‐H. Chen , and W. Shu , “Nickel‐Catalyzed Reductive Amination of Aryl Halides with Ammonia,” Angewandte Chemie International Edition 60 (2021): 9875–9880.33539628

[12] M. Karimzadeh , K. Niknam , N. Manouchehri , and D. Tarokh , “A Green Route for the Cross‐Coupling of Azide Anions with Aryl Halides under Both Base and Ligand‐Free Conditions,” RSC Advances 8 (2018): 25785–25793, 10.1039/c8ra04608e. 35539779 PMC9082577

[13] V. Nair , R. S. Menon , A. Jose , C. R. Sinu , and A. P. Thankachan , “Aryl Azides as Versatile Intermediates in Organic Synthesis,” Organic Letters 21, no. 5 (2019): 1657–1663, 10.1021/acs.orglett.9b00331.

[14] M. Z. C. Hatit , L. F. Reichenbach , J. M. Tobin , F. Vilela , G. A. Burley , and A. J. B. Watson , “Photocatalytic Arylation of Heteroarenes Using Diazonium Salts,” Nature Communications 9 (2018): 4021. 10.1038/s41467-018-06551-0 PMC6167327 30275543

[15] S. Lu , J. Zhang , L. Lang , et al., “Amino Acids to Reduce the Escape of Organic Amines in the CO_2_ Capture Process,” Separation and Purification Technology 350 (2024): 127659, 10.1016/j.seppur.2024.127659.

[16] F. F. Sead , V. Jain , S. Ballal , et al., “ZnFe_2_O_4_–SiO_2_@PC–Ni Nanoparticles for One‐Pot, Solvent‐Free Synthesis of Imidazo[1,2‐a]pyridines by A3 Coupling Reactions Under Ultrasound Conditions,” Journal of the Indian Chemical Society 102 (2025): 101653, 10.1016/j.jics.2025.101653.

[17] Z. Abbasi , Y. Mansoori , S. Fekri , and T. Çetin , “Magnetically Retrievable 2‐(2‐Pyridyl)benzimidazole‐Cu(I) on SBA‐15@Fe_3_O_4_ for Sodium Azide‐Induced Amination of Aryl Halides,” Scientific Reports 15, no. 1 (2025), 27812, 10.1038/s41598-025-13073-5. 40738945 PMC12310940

[18] S. Roy , R. Chatterjee , P. Kisan , and R. Dandela , “Ultrasound‐Assisted Synthesis of 1,5‐Disubstituted Pyrazoles via HFIP‐Mediated Cascade Cyclization of Enaminones with Aryl Hydrazine,” Tetrahedron Letters 149 (2024): 155277, 10.1016/j.tetlet.2024.155277.

[19] Z. Moradi and A. Ghorbani‐Choghamarani , “Fe_3_O_4_@SiO2@KIT‐6@2‐ATP@CuI as a Catalyst for Hydration of Benzonitriles and Reduction of Nitroarenes,” Scientific Reports 13 (2023): 7645, 10.1038/s41598-023-34409-z. 37169905 PMC10175259

[20] F. F. Sead , V. Jain , R. M. M. M. Kundlas , et al., “Magnetically Recoverable Catalysts for Efficient Multicomponent Synthesis of Organosulfur Compounds,” RSC Advances 15 (2025): 3928–3953, 10.1039/d4ra08769k. 39917045 PMC11799890

[21] N. Taghavi , Y. Mansoori , S. Fekri , Y. Akinay , and T. Çetin , “Cu(II)‐Aminotetrazole Complex Anchored on SBA‐15: A High‐Performance Catalyst for the Synthesis of 1,4‐Disubstituted‐1,2,3‐Triazoles,” Catalysis Letters 155 (2025): 341, 10.1007/s10562-025-05179-2.

[22] J. Zhao , Y. Liu , H. Wang , L. Chen , and X. Zhang , “Core‐Shell Structured ZnFe_2_O_4_@SiO_2_ Nanocomposites: Synthesis, Characterization and Applications,” Journal of Materials Chemistry A 8 (2020): 1234–1245, 10.1039/c9ta12345f.

[23] F. Andish‐Lifshagerd , A. Habibi‐Yangjeh , M. Habibi , and Y. Akinay , “Facile Synthesis of ZnO/CeO2/CeFeO_3_ Photocatalysts Sensitive to Visible Light with Tandem n‐n Heterojunctions and Improved Performance towards Tetracycline and Dye Effluent Removals,” Journal of Photochemistry and Photobiology A: Chemistry 448 (2024): 115351, 10.1016/j.jphotochem.2023.115351.

[24] F. F. Sead , V. Jain , S. Ballal , et al., “Research on Transition Metals for the Multicomponent Synthesis of Benzo‐Fused γ‐Lactams,” RSC Advances 15 (2025): 2334–2346, 10.1039/D4RA08798D. 39867320 PMC11756498

[25] I. Ahmad , M. K. Abosaoda , Y. Jadeja , et al., “A Comprehensive Review on Carbonylation Reactions: Catalysis by Magnetic Nanoparticles Supported Transition Metals,” Nanoscale Advances 7 (2025): 3189–3209, 10.1039/D5NA00040H. 40303976 PMC12035756

[26] P. K. Nancy , R. Verma , P. Thakur , and A. Thakur , “Structural and Optical Characterizations of ZnFe_2_O_4_, CoFe_2_O_4_ and Their Nanocomposites,” Energy and Environment Advances 1, no. 3 (2024): 120–128, 10.69626/eea.2024.0120.

[27] F. F. Sead , V. Jain , R. R., et al., “SiO_2_@Benzothiazole‐Cl@Fc as an Efficient Heterogeneous Catalyst for the Synthesis of 1,3,5‐Trisubstituted Pyrazoles by A3 Coupling,” ChemistryOpen 14 (2025): e202500024, 10.1002/open.202500024. 40357693 PMC12368891

[28] M. Mahdavi , M. A. Ghasemzadeh , and A. Javadi , “Synthesis of ZIF‐8/ZnFe_2_O_4_/GO‐OSO_3_H Nanocomposite as a Superior and Reusable Heterogeneous Catalyst for the Preparation of Pyrimidine Derivatives and Investigation of Their Antimicrobial Activities,” Heliyon 10 (2024): e26339, 10.1016/j.heliyon.2024.e26339. 38420459 PMC10900959

[29] S. R. Shingda , P. H. Hadole , H. M. Alone , et al., “Phyto‐Fabrication and Characterization of ZnFe_2_O_4_@AC Nanocomposite Catalyst via Green Pathway and Its Application for the Synthesis of Some Quinazolin‐4(1H)‐One Derivatives,” Polyhedron 270 (2025): 117438, 10.1016/j.poly.2025.117438.

[30] S. Punyasamudram , R. P. Puthalapattu , A. Bathinapatla , S. Kanchi , S. Jyothi , and P. V. N. Kumar , “Biosynthesis of ZnFe_2_O_4_@Ag Hybrid Nanocomposites for Degradation of 2,4‐Dichlorophenoxyacetic Acid Herbicide,” Chemical Physics Impact 7 (2023): 100282, 10.1016/j.chphi.2023.100282.

[31] I. P. Beletskaya and A. D. Averin , “Metal‐Catalyzed Reactions for the C(sp^2^)–N Bond Formation: Achievements of Recent Years,” Russian Chemical Reviews 90 (2021): 1359–1396, 10.1070/rcr4999.

[32] Z. Tashrifi , M. M. Khanaposhtani , B. Larijani , and M. Mahdavi , “Sodium Azide: An Inorganic Nitrogen Source for the Synthesis of Organic N‐Compounds,” ChemistrySelect 6 (2021): 13419–13433, 10.1002/slct.202103271.

[33] M. C. S. Nisha , R. Kumar , and Y. Kumar , “Regioselective Copper(I)‐Catalyzed Ullmann Amination of Halopyridyl Carboxylates Using Sodium Azide: A Route for Aminopyridyl Carboxylates and Their Transformation to Pyrido[2,3‐d]pyrimidin‐4(1H)‐Ones,” ChemistrySelect 3 (2018): 4822–4826, 10.1002/slct.201800907.

[34] Á. Georgiádes , S. B. Ötvös , and F. Fülöp , “Controlled Transformations of Aryl Halides in a Flow System: Selective Synthesis of Aryl Azides and Aniline Derivatives,” Advanced Synthesis and Catalysis 360 (2018): 1841–1849, 10.1002/adsc.201701539.

[35] S. Paik , “Selective Copper‐Catalyzed Azidation and Amination of Aryl Halides with Sodium Azide,” Applied Chemical Engineering 32 (2021): 224–227, 10.14478/ace.2021.1012.

[36] A. Vijeta , C. Casadevall , and E. Reisner , “An Integrated Carbon Nitride–nickel Photocatalyst for the Amination of Aryl Halides Using Sodium Azide,” Angewandte Chemie International Edition 61 (2022): e202203176, 10.1002/anie.202203176. 35332981 PMC9321912

[37] Y. Akinay and I. N. Akkuş , “Synthesis and Characterization of the Pearlescent Pigments Based on Mica Deposited with SiO_2_, AlN and TiO_2_: First Report of Its Dielectric Properties,” Ceramics International 46 (2020): 17735–17740, 10.1016/j.ceramint.2020.04.078.

[38] S. Graßl , J. Singer , and P. Knochel , “Iron‐Mediated Electrophilic Amination of Organozinc Halides Using Organic Azides,” Angewandte Chemie International Edition 59 (2020): 335–338, 10.1002/anie.201911704. 31599056 PMC6972526

[39] H. Veisi , M. Pirhayati , P. Mohammadi , T. Tamoradi , S. Hemmati , and B. Karmakar , “Recent Advances in the Application of Magnetic Nanocatalysts in Multicomponent Reactions,” RSC Advances 13 (2023): 20530, 10.1039/d3ra01208e. 37435379 PMC10331794

[40] H. Li and D. W. C. MacMillan , “Photoredox Catalysis for Aryl Amine Synthesis,” Science 359 (2018): 1109–1114, 10.1126/science.aao0170.

[41] M. Roemer , I. Luck , and N. Proschogo , “Cu(I)‐Mediated Azidation of Halobenzenes, and Cu‐Catalyzed Selective Azide Reduction to Corresponding Amines,” Advanced Synthesis and Catalysis 364 (2022): 2957–2971, 10.1002/adsc.202200594.

[42] M. Roemer , N. Proschogo , and I. Luck , “Copper(I) Chloride‐Mediated Amination of Halobenzenes via Azides: Scope, Mechanistic Aspects, and C–C Cleavage Reactions,” Journal of Organic Chemistry 88 (2023): 1522–1532, 10.1021/acs.joc.2c02549. 36668998

[43] I. V. Machado , J. R. N. dos Santos , M. A. P. Januario , and A. G. Corrêa , “Greener Organic Synthetic Methods: Sonochemistry and Heterogeneous Catalysis Promoted Multicomponent Reactions,” Ultrasonics Sonochemistry 78 (2021): 105704, 10.1016/j.ultsonch.2021.105704. 34454180 PMC8406036

[44] T. J. Mason , J. P. Lorimer , D. M. Bates , and Y. Zhao , “Sonochemistry: The use of Ultrasound in Synthesis and Processing,” Chemical Society Reviews 36 (2007): 109–117, 10.1039/b611455j.

[45] R. Javahershenas and S. Nikzat , “Recent Developments Using Malononitrile in Ultrasound‐Assisted Multicomponent Synthesis of Heterocycles,” Ultrasonics Sonochemistry 102 (2024): 106741, 10.1016/j.ultsonch.2023.106741. 38176128 PMC10793181

[46] V. P. Ananikov , “Green Chemistry and Ultrasound‐Assisted Organic Synthesis,” Chemical Society Reviews 44 (2015): 2752–2766, 10.1039/c4cs00393f.

[47] J. T. Markiewicz , O. Wiest , and P. Helquist , “Synthesis of Primary Aryl Amines through a Copper‐Assisted Aromatic Substitution Reaction with Sodium Azide,” Journal of Organic Chemistry 75 (2010): 4887–4890, 10.1021/jo101002p. 20568788

[48] Y. Goriya and C. V. Ramana , “The [Cu]‐Catalyzed SNAr Reactions: Direct Amination of Electron‐Deficient Aryl Halides with Sodium Azide and the Synthesis of Arylthioethers under Cu(II)/Ascorbate Redox System,” Tetrahedron 66 (2010): 7642–7650, 10.1016/j.tet.2010.07.032.

[49] S. Fekri , Y. Mansoori , Y. Akinay , T. Çetin , and M. John , “An Efficient Supported Cu(I) Catalyst for the Amination of Aryl Halides with Sodium Azide,” ChemNanoMat 10 (2024): e202300622, 10.1002/cnma.202300622.

[50] S. Messaoudi , J. D. Brion , and M. Alami , “An Expeditious Copper‐Catalyzed Access to 3‐Aminoquinolinones, 3‐Aminocoumarins and Anilines Using Sodium Azide,” Advanced Synthesis and Catalysis 352 (2010): 1677–1687, 10.1002/adsc.201000149.

[51] M. Jaina , M. Yadav , T. Kohout , M. Lahtinen , V. K. Garge , and M. Sillanpää , “Development of Iron Oxide/Activated Carbon Nanoparticle Composite for the Removal of Cr(VI), Cu(II) and Cd(II) Ions from Aqueous Solution,” Water Resources and Industry 20 (2018): 54–74, 10.1016/j.wri.2018.10.001.

