# Supplementary material for: ZnFe2O4@SiO2@AC Magnetic Nanocomposite as an Efficient Catalyst for Ultrasound‐Assisted Azidation of Aryl Halides to Aryl Amines
Source: ChemistryOpen. 2026 Mar 29;15(4):e70172. doi: 10.1002/open.70172 (PMC13140919; doi:10.1002/open.70172)
Supplement: Supplementary file 1 — Supplementary Material [file OPEN-15-e70172-s001.pdf]

$ZnFe_2O_4$@SiO_2@AC Nanocomposite:

A Novel Approach Azidation of Aryl Halides to Aryl Amines under ultrasound conditions

Manish Kumar[1], Rima Heider Al Omari[2], Soumya V. Menon[3], Shaker Al-Hasnaawei (a,b)[4]

Ahmad Sajjadi[5*], Amrita Pal[6], Renu Sharma[7], Aashna Sinha[8]

1

Manish Kumar

Department of Electronics and Communication Engineering, GLA University, Mathura-281406, India.

Mail: manish.kumar@gla.ac.in

2

Rima Heider Al Omari
Faculty of Allied Medical Sciences, Hourani Center for Applied Scientific Research, Al-Ahliyya Amman University, Amman, Jordan.
Mail: r.alomari@ammanu.edu.jo

3

Soumya V. Menon
Department of Chemistry and Biochemistry, School of Sciences, JAIN (Deemed to be University), Bangalore, Karnataka, India.
Mail: v.soumya@jainuniversity.ac.in

4

Shaker Al-Hasnaawei (a,b)

[a] College of pharmacy, the Islamic University, Najaf, Iraq.

[b] Department of medical analysis, Medical laboratory technique college, the Islamic University of Al Diwaniyah, Al Diwaniyah, Iraq.

Mail: shakeralhasnawi@iunajaf.edu.iq

5

Ahmad Sajjadi

Young Researchers and Elite Club, Tehran Branch, Islamic Azad University, Tehran, Iran.

sajjadiahmmad@gmail.com

https://orcid.org/0009-0005-4223-2576

6

Amrita Pal

Department of Chemistry, Sathyabama Institute of Science and Technology, Chennai, Tamil Nadu, India.

Mail: amritapal.chemistry@sathyabama.ac.in

7

Renu Sharma

Department of Chemistry, University Institute of Sciences, Chandigarh University, Mohali, Punjab, India.

Mail: drrenusharma01@outlook.com

8

Aashna Sinha

School of Applied and Life Sciences, Division of Research and Innovation, Uttaranchal University, Dehradun, Uttarakhand, Indian.

Mail: aashna07sinha@gmail.com

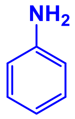

Aniline

(Liquid)

^{1}H NMR (400 MHz, DMSO) δ 7.13 (t, *J* = 7.8 Hz, 2H), 6.76 (d, *J* = 8.4 Hz, 2H), 6.67 (t, *J* = 7.5 Hz, 1H), 5.02 (s, 2H); ^{13}C NMR (100 MHz, DMSO) δ 148.1, 131.5, 129.7, 115.6 ppm.

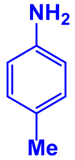

p-toluidine

(M.P: 41-43 ^{o}C)

^{1}H NMR (400 MHz, DMSO) δ 7.04 (d, *J* = 8.0 Hz, 2H), 6.42 (d, *J* = 7.4 Hz, 2H), 4.94 (s, 2H), 2.36 (s, 3H); ^{13}C NMR (100 MHz, DMSO) δ 145.2, 131.0, 129.5, 115.4, 22.7 ppm.

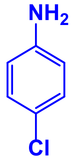

(M.P: 71-73 ^{o}C)

4-chloroaniline

(M.P: 41-43 ^{o}C)

^{1}H NMR (400 MHz, DMSO) δ 7.11 (d, *J* = 6.8 Hz, 2H), 6.64 (d, *J* = 7.7 Hz, 2H), 5.51 (s, 2H); ^{13}C NMR (100 MHz, DMSO) δ 146.5, 129.8, 127.1, 116.4 ppm.

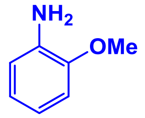

2-methoxyaniline

(colorless oil)

^{1}H NMR (400 MHz, DMSO) δ 6.92-6.80 (m, 4H), 4.96 (s, 2H), 3.84 (s, 3H); ^{13}C NMR (100 MHz, DMSO) δ 147.4, 137.2, 123.4, 121.7, 115.6, 110.8, 55.1 ppm.

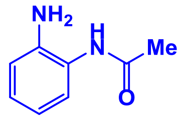

N-(2-aminophenyl)acetamide

(M.P: 126-128 °C)

^{1}H NMR (400 MHz, DMSO) δ 9.30 (s, 1H), 7.78 (d, *J* = 7.6 Hz, 1H), 7.44 (t, *J* = 8.0 Hz, 1H), 7.01 (d, *J* = 8.0 Hz, 1H), 6.88 (t, *J* = 7.3 Hz, 1H), 5.09 (s, 2H); ^{13}C NMR (100 MHz, DMSO) δ 168.1, 149.3, 125.4, 118.7, 114.0, 24.5 ppm.

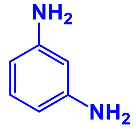

benzene-1,3-diamine

(yellow oil)

^{1}H NMR (400 MHz, DMSO) δ 7.05 (t, *J* = 8.5 Hz, 2H), 6.19 (d, *J* = 7.4 Hz, 2H), 6.09 (s, 1H), 5.13 (s, 2H); ^{13}C NMR (100 MHz, DMSO) δ 147.8, 130.2, 105.2, 100.4 ppm.

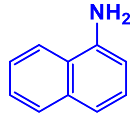

naphthalen-1-amine

(M.P: 46-48 °C)

^{1}H NMR (400 MHz, DMSO) δ 8.07 (d, *J* = 7.7 Hz, 2H), 7.62 (d, *J* = 7.7 Hz, 1H), 7.47 (t, *J* = 7.6 Hz, 2H), 7.16 (t, *J* = 8.4 Hz, 1H), 6.76 (d, *J* = 7.9 Hz, 1H), 5.58 (s, 2H); ^{13}C NMR (100 MHz, DMSO) δ 143.2, 134.5, 129.0, 126.1, 125.6, 123.4, 121.0, 119.7, 109.4 ppm.

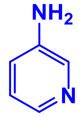

pyridin-3-amine

(M.P: 60-62 °C)

^{1}H NMR (400 MHz, DMSO) δ 8.03 (s, 1H), 7.78 (d, *J* = 6.7 Hz, 1H), 7.35 (t, *J* = 7.8 Hz, 1H), 7.09 (d, *J* = 8.0 Hz, 1H), 6.61 (s, 2H); ^{13}C NMR (100 MHz, DMSO) δ 145.3, 138.1, 137.6, 124.2, 122.7 ppm.

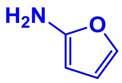

furan-2-amine

(Liquid)

^{1}H NMR (400 MHz, DMSO) δ 7.84 (d, *J* = 7.7 Hz, 1H), 7.02 (d, *J* = 7.2 Hz, 1H), 6.67 (d, *J* = 8.0 Hz, 1H), 6.20 (s, 2H); ^{13}C NMR (100 MHz, DMSO) δ 144.5, 143.2, 109.1, 108.4 ppm.

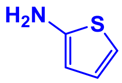

thiophen-2-amine

(Liquid)

^{1}H NMR (400 MHz, DMSO) δ 7.39 (s, 2H), 6.70 (d, *J* = 7.6 Hz, 1H), 6.51 (t, *J* = 7.8 Hz, 1H), 6.02 (d, *J* = 6.9 Hz, 1H); ^{13}C NMR (100 MHz, DMSO) δ 138.2, 122.3, 118.9 ppm.

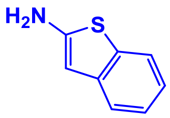

benzo[b]thiophen-2-amine

(M.P: 98-100 °C)

^{1}H NMR (400 MHz, DMSO) δ 7.95 (d, *J* = 7.5 Hz, 1H), 7.76 (d, *J* = 7.7 Hz, 1H), 7.52 (t, *J* = 8.0 Hz, 1H), 7.44 (t, *J* = 6.9 Hz, 1H), 7.32 (s, 1H), 6.36 (s, 2H); ^{13}C NMR (100 MHz, DMSO) δ 139.8, 126.2, 124.1, 123.7, 122.3 ppm.

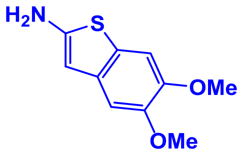

5,6-dimethoxybenzo[b]thiophen-2-amine

(M.P: 120-122 °C)

^{1}H NMR (400 MHz, DMSO) δ 8.29 (s, 1H), 7.42 (s, 2H), 7.38 (s, 1H), 6.31 (s, 1H), 3.93 (s, 3H), 3.84 (s, 3H); ^{13}C NMR (100 MHz, DMSO) δ 152.1, 147.4, 133.2, 126.9, 123.6, 107.2, 104.8, 56.4 ppm.

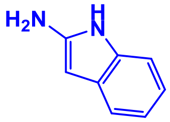

1H-indol-2-amine

(Liquid)

^{1}H NMR (400 MHz, DMSO) δ 11.52 (s, 1H), 7.66 (d, *J* = 8.5 Hz, 1H), 7.53 (d, *J* = 7.4 Hz, 1H), 7.03 (t, *J* = 8.0 Hz, 2H), 6.75 (s, 1H), 6.33 (s, 2H); ^{13}C NMR (100 MHz, DMSO) δ 135.6, 128.7, 124.3, 122.1, 120.5, 119.3, 111.4, 102.7 ppm.

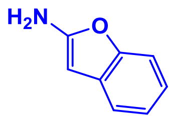

benzofuran-2-amine

(Liquid)

^{1}H NMR (400 MHz, DMSO) δ 7.61 (d, *J* = 6.8 Hz, 2H), 7.48 (t, *J* = 7.6 Hz, 1H), 7.22 (t, *J* = 7.0 Hz, 1H), 7.09 (s, 1H), 6.22 (s, 2H); ^{13}C NMR (100 MHz, DMSO) δ 156.1, 145.2, 128.3, 124.1, 123.7, 120.5, 115.0, 105.4 ppm.

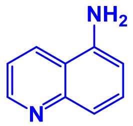

quinolin-5-amine

(M.P: 105-107 °C)

^{1}H NMR (400 MHz, DMSO) δ 8.89 (d, *J* = 9.0 Hz, 1H), 8.38 (d, *J* = 8.3 Hz, 1H), 7.64-7.53 (m, 3H), 7.42 (d, *J* = 7.6 Hz, 1H), 5.78 (s, 2H); ^{13}C NMR (100 MHz, DMSO) δ 149.1, 145.3, 138.4, 131.0, 121.4, 118.7, 116.6, 107.8 ppm.

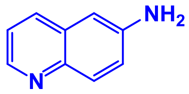

quinolin-6-amine

(M.P: 113-115 °C)

^{1}H NMR (400 MHz, DMSO) δ 8.73 (dd, *J* = 9.3, 5.1 Hz, 1H), 8.49 (d, *J* = 7.3 Hz, 1H), 8.33 (d, *J* = 7.6 Hz, 1H), 7.47 (d, *J* = 8.4 Hz, 1H), 7.36 (t, *J* = 7.7 Hz, 1H), 6.62 (s, 1H), 4.61 (s, 2H); ^{13}C NMR (100 MHz, DMSO) δ 147.0, 145.3, 141.1, 132.9, 131.7, 129.8, 120.3, 104.2 ppm.

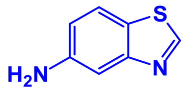

benzo[d]thiazol-5-amine

(M.P: 71-73 °C)

^{1}H NMR (400 MHz, DMSO) δ 9.39 (s, 1H), 7.62 (d, *J* = 7.2 Hz, 1H), 6.85 (s,1H), 6.58 (d, *J* = 7.9 Hz, 1H), 5.21 (s, 2H); ^{13}C NMR (100 MHz, DMSO) δ 156.1, 154.3, 147.5, 128.0, 122.3, 113.4, 106.7 ppm.

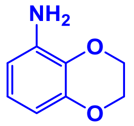

2,3-dihydrobenzo[b][1,4]dioxin-5-amine

(Liquid)

^{1}H NMR (400 MHz, DMSO) δ 6.79 (t, *J* = 7.4 Hz, 1H), 6.51 (t, *J* = 8.0 Hz, 1H), 6.17 (d, *J* = 7.6 Hz, 1H), 5.29 (s, 2H), 4.23 (s, 4H); ^{13}C NMR (100 MHz, DMSO) δ 147.0, 137.6, 131.2, 121.5, 106.7, 105.4, 64.3 ppm.

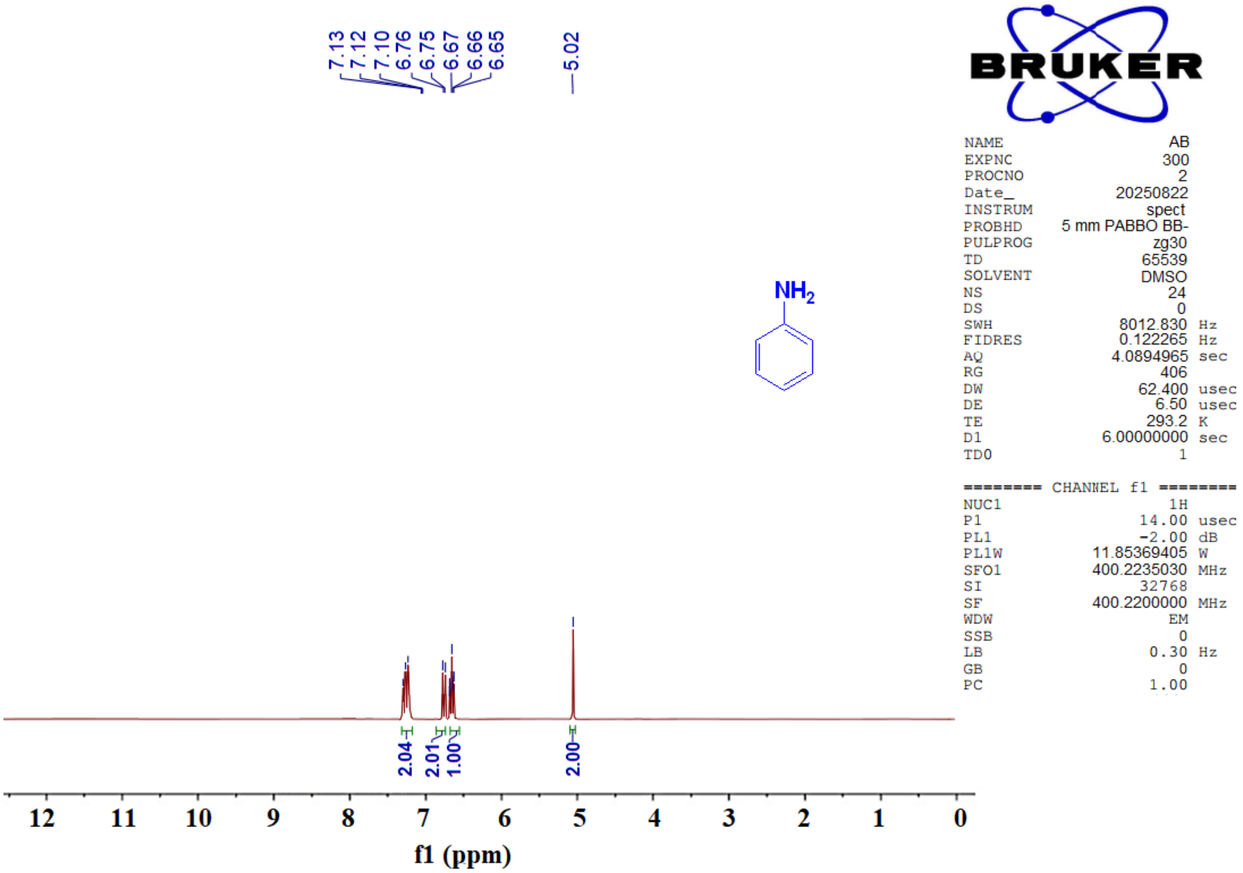

7.13
7.12
7.10
6.76
6.75
6.67
6.66
6.65
5.02
NH2
2.04
2.01
1.00
2.00
12
11
10
9
8
7
6
5
4
3
2
1
0
f1 (ppm)
NAME AB
EXPNC 300
PROCNO 2
Date_ 20250822
INSTRUM spect
PROBHD 5 mm PABBO BB-
PULPROG zg30
TD 65539
SOLVENT DMSO
NS 24
DS 0
SWH 8012.830 Hz
FIDRES 0.122265 Hz
AQ 4.0894965 sec
RG 406
DW 62.400 usec
DE 6.50 usec
TE 293.2 K
D1 6.00000000 sec
TD0 1
======== CHANNEL f1 ========
NUC1 1H
P1 14.00 usec
PL1 -2.00 dB
PL1W 11.85369405 W
SFO1 400.2235030 MHz
SI 32768
SF 400.2200000 MHz
WDW EM
SSB 0
LB 0.30 Hz
GB 0
PC 1.00

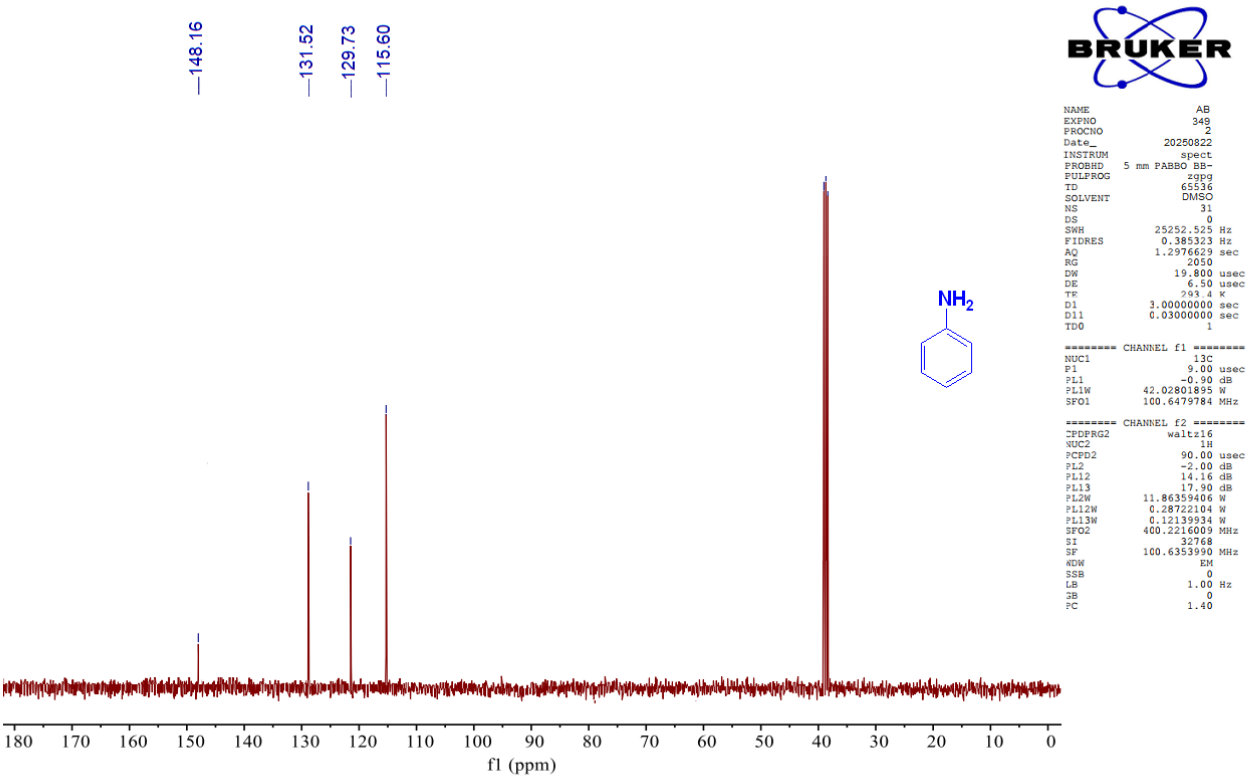

148.16
131.52
129.73
115.60
NH2
180
170
160
150
140
130
120
110
100
90
80
70
60
50
40
30
20
10
0
f1 (ppm)
NAME AB
EXPNO 349
PROCNO 2
Date_ 20250822
INSTRUM spect
PROBHD 5 mm PABBO BB-
PULPROG zgpg
TD 65536
SOLVENT DMSO
NS 31
DS 0
SWH 25252.525 Hz
FIDRES 0.385323 Hz
AQ 1.2976629 sec
RG 2050
DW 19.800 usec
DE 6.50 usec
TE 293.4 K
D1 3.00000000 sec
D11 0.03000000 sec
TD0 1
======== CHANNEL f1 ========
NUC1 13C
P1 9.00 usec
PL1 -0.90 dB
PL1W 42.02801895 W
SFO1 100.6479784 MHz
======== CHANNEL f2 ========
CPDPRG2 waltz16
NUC2 1H
PCPD2 90.00 usec
PL2 -2.00 dB
PL12 14.16 dB
PL13 17.90 dB
PL2W 11.86359406 W
PL12W 0.28722104 W
PL13W 0.12139934 W
SFO2 400.2216009 MHz
SI 32768
SF 100.6353990 MHz
WDW EM
SSB 0
LB 1.00 Hz
GB 0
PC 1.40

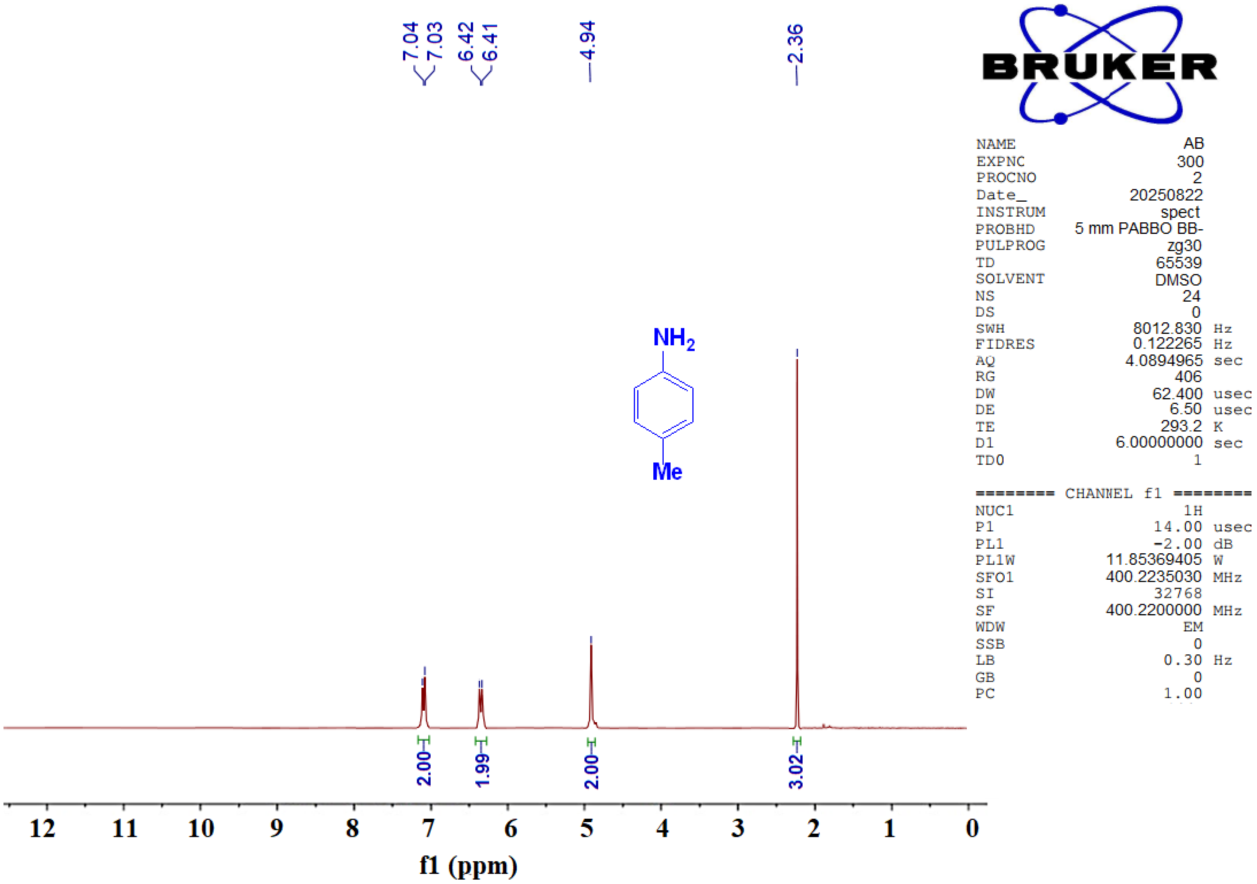

7.04
7.03
6.42
6.41
4.94
2.36
BRUKER
NAME AB
EXPNO 300
PROCNO 2
Date_ 20250822
INSTRUM spect
PROBHD 5 mm PABBO BB-
PULPROG zg30
TD 65539
SOLVENT DMSO
NS 24
DS 0
SWH 8012.830 Hz
FIDRES 0.122265 Hz
AQ 4.0894965 sec
RG 406
DW 62.400 usec
DE 6.50 usec
TE 293.2 K
D1 6.00000000 sec
TD0 1
======== CHANNEL f1 ========
NUC1 1H
P1 14.00 usec
PL1 -2.00 dB
PL1W 11.85369405 W
SFO1 400.2235030 MHz
SI 32768
SF 400.2200000 MHz
WDW EM
SSB 0
LB 0.30 Hz
GB 0
PC 1.00
NH2
Me
2.00
1.99
2.00
3.02
12 11 10 9 8 7 6 5 4 3 2 1 0
f1 (ppm)

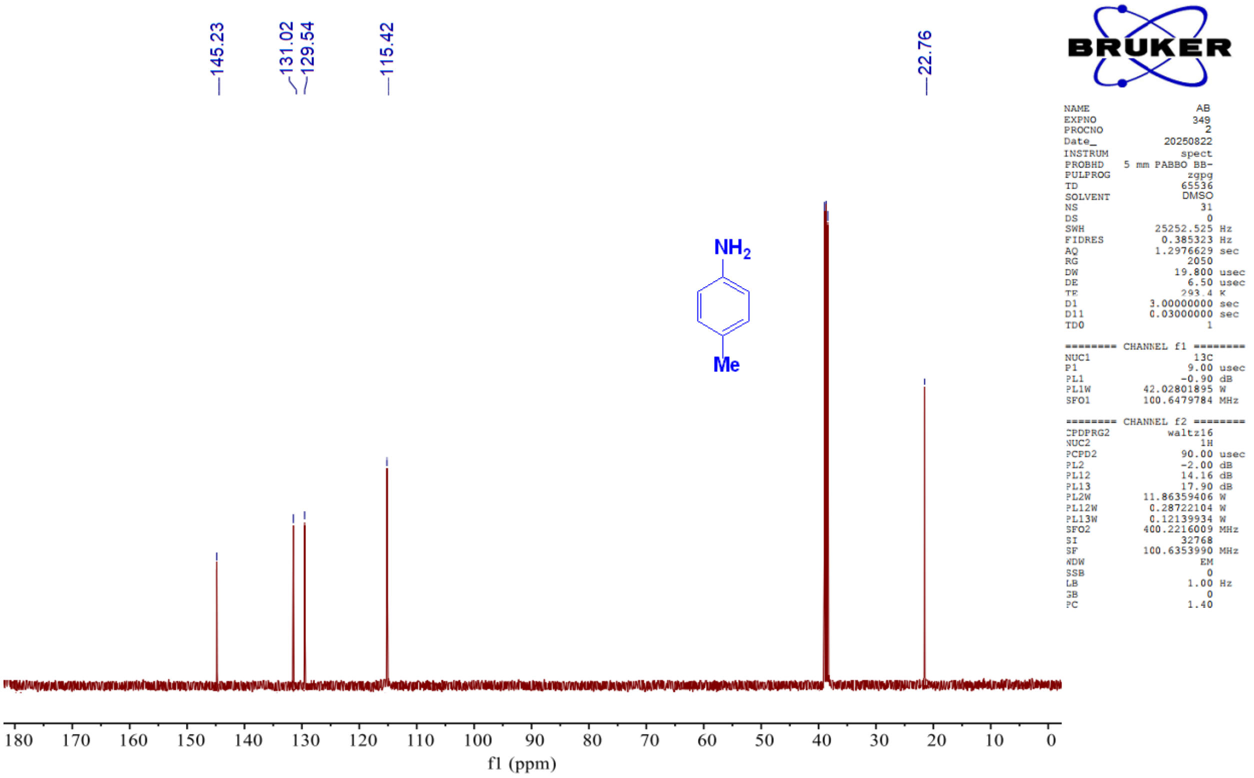

145.23
131.02
129.54
115.42
22.76
BRUKER
NAME AB
EXPNO 349
PROCNO 2
Date_ 20250822
INSTRUM spect
PROBHD 5 mm PABBO BB-
PULPROG zgpg
TD 65536
SOLVENT DMSO
NS 31
DS 0
SWH 25252.525 Hz
FIDRES 0.385323 Hz
AQ 1.2976629 sec
RG 2050
DW 19.800 usec
DE 6.50 usec
TE 293.4 K
D1 3.00000000 sec
D11 0.03000000 sec
TD0 1
======== CHANNEL f1 ========
NUC1 13C
P1 9.00 usec
PL1 -0.90 dB
PL1W 42.02801895 W
SFO1 100.6479784 MHz
======== CHANNEL f2 ========
CPDPRG2 waltz16
NUC2 1H
PCPD2 90.00 usec
PL2 -2.00 dB
PL12 14.16 dB
PL13 17.90 dB
PL2W 11.86359406 W
PL12W 0.28722104 W
PL13W 0.12139934 W
SFO2 400.2216009 MHz
SI 32768
SF 100.6353990 MHz
WDW EM
SSB 0
LB 1.00 Hz
GB 0
PC 1.40
NH2
Me
180 170 160 150 140 130 120 110 100 90 80 70 60 50 40 30 20 10 0
f1 (ppm)

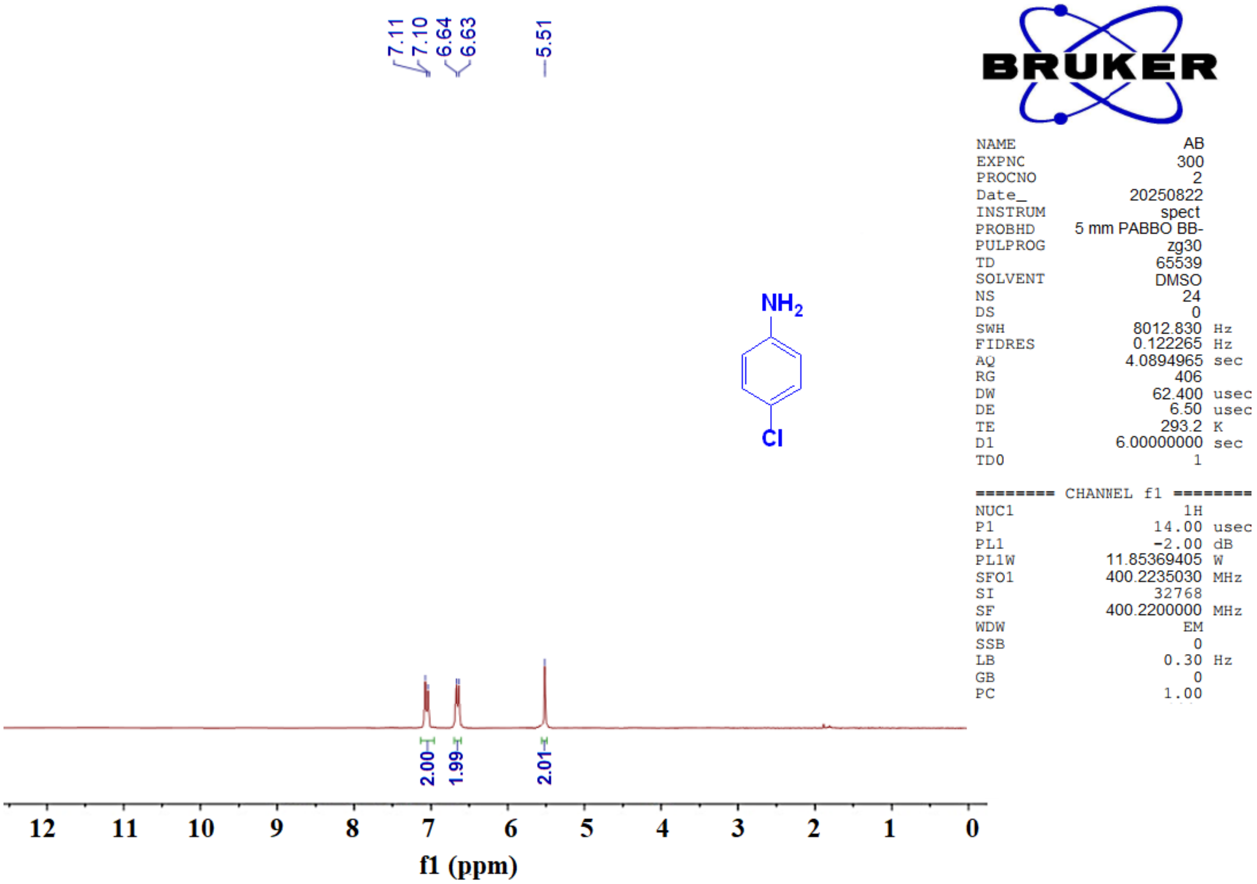
7.11
7.10
6.64
6.63
5.51
BRUKER
NAME AB
EXPNC 300
PROCNO 2
Date_ 20250822
INSTRUM spect
PROBHD 5 mm PABBO BB-
PULPROG zg30
TD 65539
SOLVENT DMSO
NS 24
DS 0
SWH 8012.830 Hz
FIDRES 0.122265 Hz
AQ 4.0894965 sec
RG 406
DW 62.400 usec
DE 6.50 usec
TE 293.2 K
D1 6.00000000 sec
TD0 1
======== CHANNEL f1 ========
NUC1 1H
P1 14.00 usec
PL1 -2.00 dB
PL1W 11.85369405 W
SFO1 400.2235030 MHz
SI 32768
SF 400.2200000 MHz
WDW EM
SSB 0
LB 0.30 Hz
GB 0
PC 1.00
NH2
Cl
2.00
1.99
2.01
12 11 10 9 8 7 6 5 4 3 2 1 0
f1 (ppm)

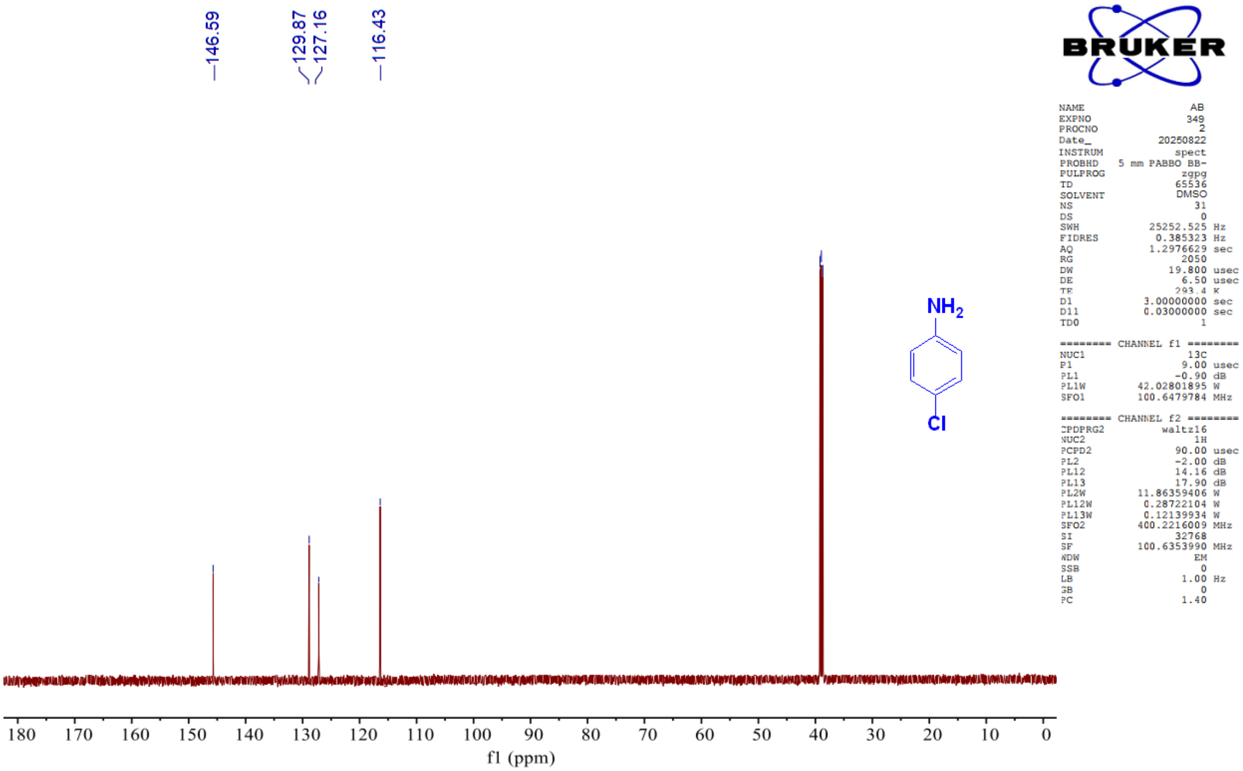
146.59
129.87
127.16
116.43
BRUKER
NAME AB
EXPNO 349
PROCNO 2
Date_ 20250822
INSTRUM spect
PROBHD 5 mm PABBO BB-
PULPROG zgpg
TD 65536
SOLVENT DMSO
NS 31
DS 0
SWH 25252.525 Hz
FIDRES 0.385323 Hz
AQ 1.2976629 sec
RG 2050
DW 19.800 usec
DE 6.50 usec
TE 293.4 K
D1 3.00000000 sec
D11 0.03000000 sec
TD0 1
======== CHANNEL f1 ========
NUC1 13C
P1 9.00 usec
PL1 -0.90 dB
PL1W 42.02801895 W
SFO1 100.6479784 MHz
======== CHANNEL f2 ========
CPDPRG2 waltz16
NUC2 1H
PCPD2 90.00 usec
PL2 -2.00 dB
PL12 14.16 dB
PL13 17.90 dB
PL2W 11.86359406 W
PL12W 0.28722104 W
PL13W 0.12139934 W
SFO2 400.2216009 MHz
SI 32768
SF 100.6353990 MHz
WDW EM
SSB 0
LB 1.00 Hz
GB 0
PC 1.40
NH2
Cl
180 170 160 150 140 130 120 110 100 90 80 70 60 50 40 30 20 10 0
f1 (ppm)

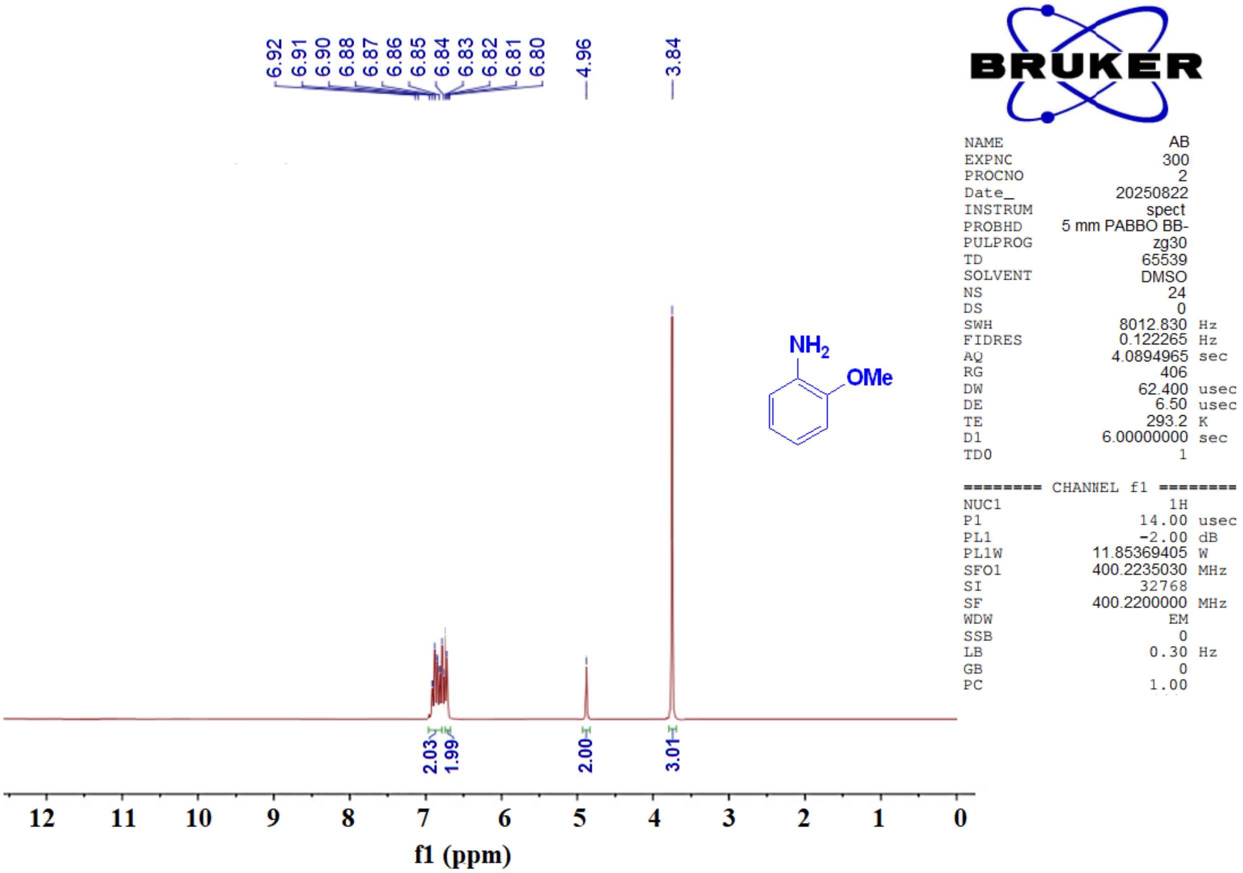
6.92
6.91
6.90
6.88
6.87
6.86
6.85
6.84
6.83
6.82
6.81
6.80
4.96
3.84
BRUKER
NAME AB
EXPNC 300
PROCNO 2
Date_ 20250822
INSTRUM spect
PROBHD 5 mm PABBO BB-
PULPROG zg30
TD 65539
SOLVENT DMSO
NS 24
DS 0
SWH 8012.830 Hz
FIDRES 0.122265 Hz
AQ 4.0894965 sec
RG 406
DW 62.400 usec
DE 6.50 usec
TE 293.2 K
D1 6.00000000 sec
TD0 1
======== CHANNEL f1 ========
NUC1 1H
P1 14.00 usec
PL1 -2.00 dB
PL1W 11.85369405 W
SFO1 400.2235030 MHz
SI 32768
SF 400.2200000 MHz
WDW EM
SSB 0
LB 0.30 Hz
GB 0
PC 1.00
NH2
OMe
2.03
1.99
2.00
3.01
f1 (ppm)

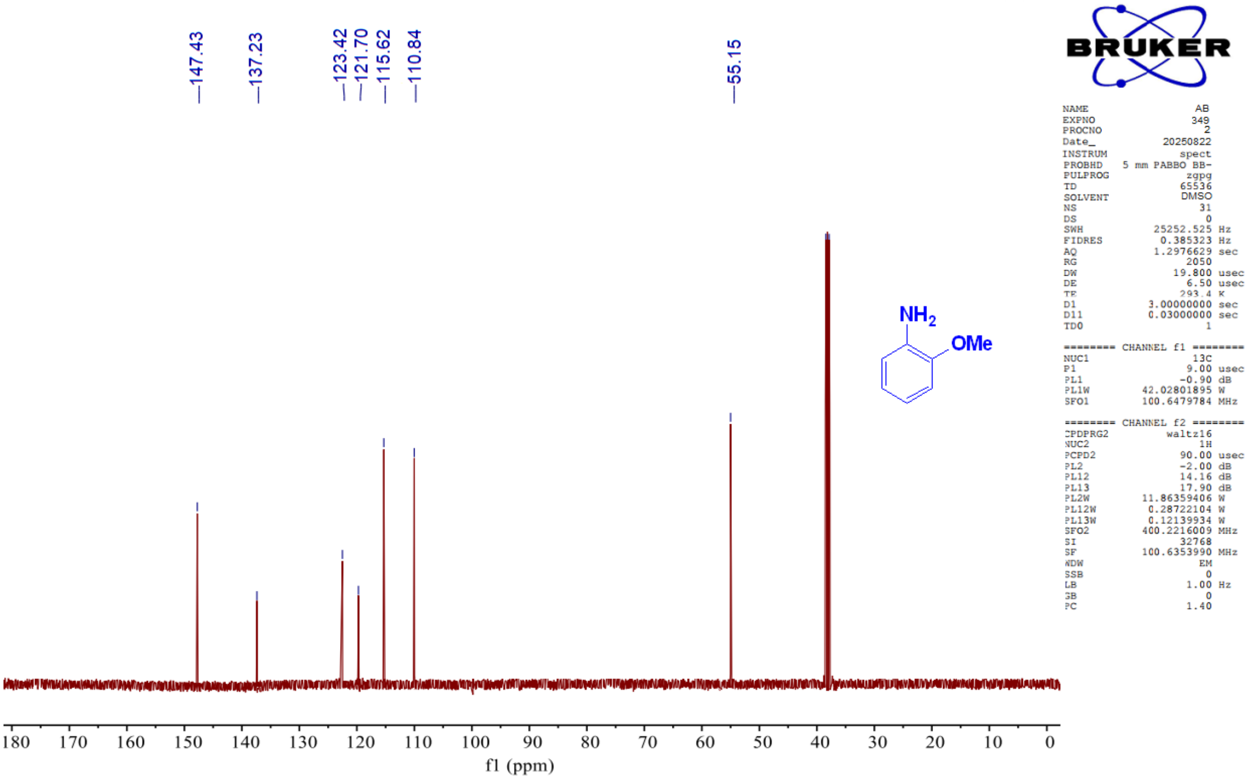
147.43
137.23
123.42
121.70
115.62
110.84
55.15
BRUKER
NH2
OMe
f1 (ppm)

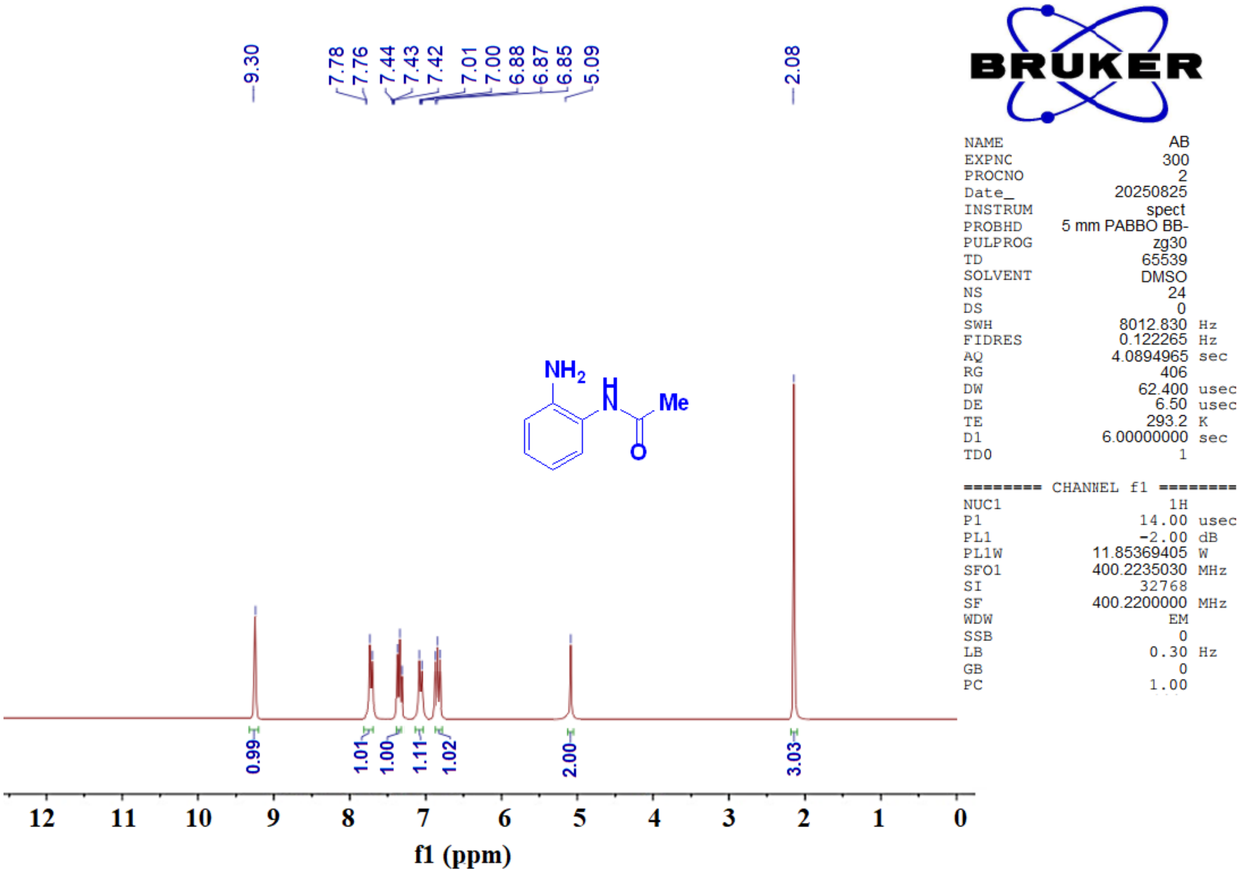

9.30
7.78
7.76
7.44
7.43
7.42
7.01
7.00
6.88
6.87
6.85
5.09
2.08
BRUKER
NAME AB
EXPNC 300
PROCNO 2
Date_ 20250825
INSTRUM spect
PROBHD 5 mm PABBO BB-
PULPROG zg30
TD 65539
SOLVENT DMSO
NS 24
DS 0
SWH 8012.830 Hz
FIDRES 0.122265 Hz
AQ 4.0894965 sec
RG 406
DW 62.400 usec
DE 6.50 usec
TE 293.2 K
D1 6.00000000 sec
TD0 1
======== CHANNEL f1 ========
NUC1 1H
P1 14.00 usec
PL1 -2.00 dB
PL1W 11.85369405 W
SFO1 400.2235030 MHz
SI 32768
SF 400.2200000 MHz
WDW EM
SSB 0
LB 0.30 Hz
GB 0
PC 1.00
NH2
H
N
Me
O
0.99
1.01
1.00
1.11
1.02
2.00
3.03
12 11 10 9 8 7 6 5 4 3 2 1 0
f1 (ppm)

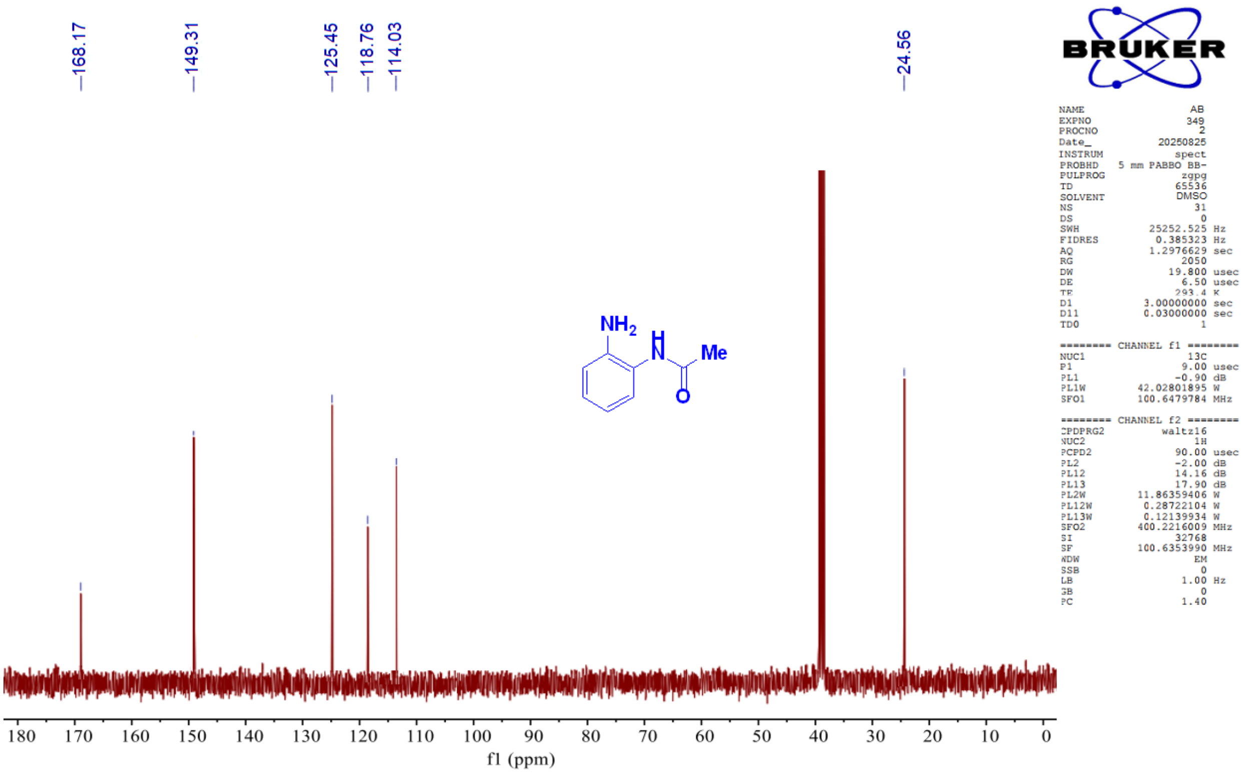

168.17
149.31
125.45
118.76
114.03
24.56
BRUKER
NH2
H
N
Me
O
180 170 160 150 140 130 120 110 100 90 80 70 60 50 40 30 20 10 0
f1 (ppm)

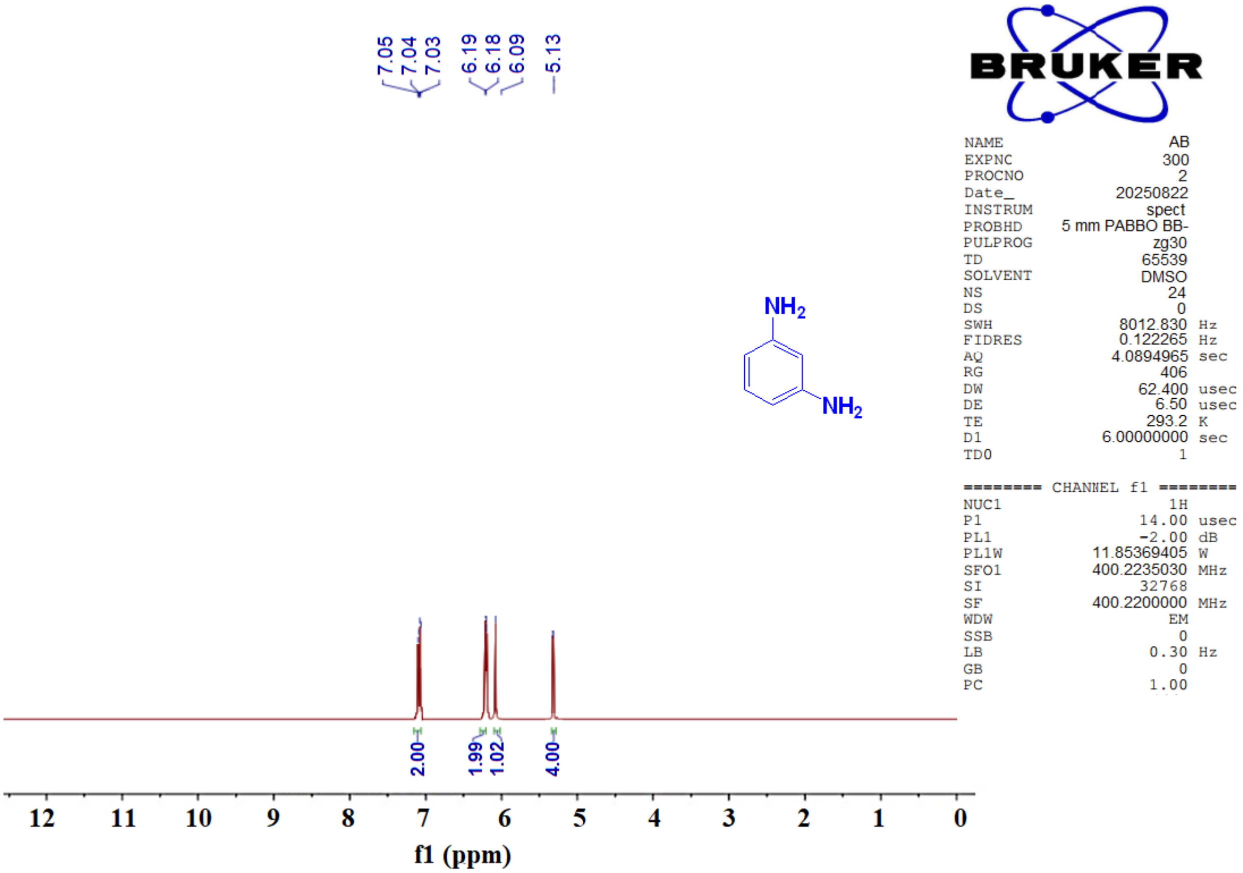

7.05
7.04
7.03
6.19
6.18
6.09
5.13
BRUKER
NAME AB
EXPNC 300
PROCNO 2
Date_ 20250822
INSTRUM spect
PROBHD 5 mm PABBO BB-
PULPROG zg30
TD 65539
SOLVENT DMSO
NS 24
DS 0
SWH 8012.830 Hz
FIDRES 0.122265 Hz
AQ 4.0894965 sec
RG 406
DW 62.400 usec
DE 6.50 usec
TE 293.2 K
D1 6.00000000 sec
TD0 1
======== CHANNEL f1 ========
NUC1 1H
P1 14.00 usec
PL1 -2.00 dB
PL1W 11.85369405 W
SFO1 400.2235030 MHz
SI 32768
SF 400.2200000 MHz
WDW EM
SSB 0
LB 0.30 Hz
GB 0
PC 1.00
NH_2
NH_2
2.00
1.99
1.02
4.00
12 11 10 9 8 7 6 5 4 3 2 1 0
f1 (ppm)

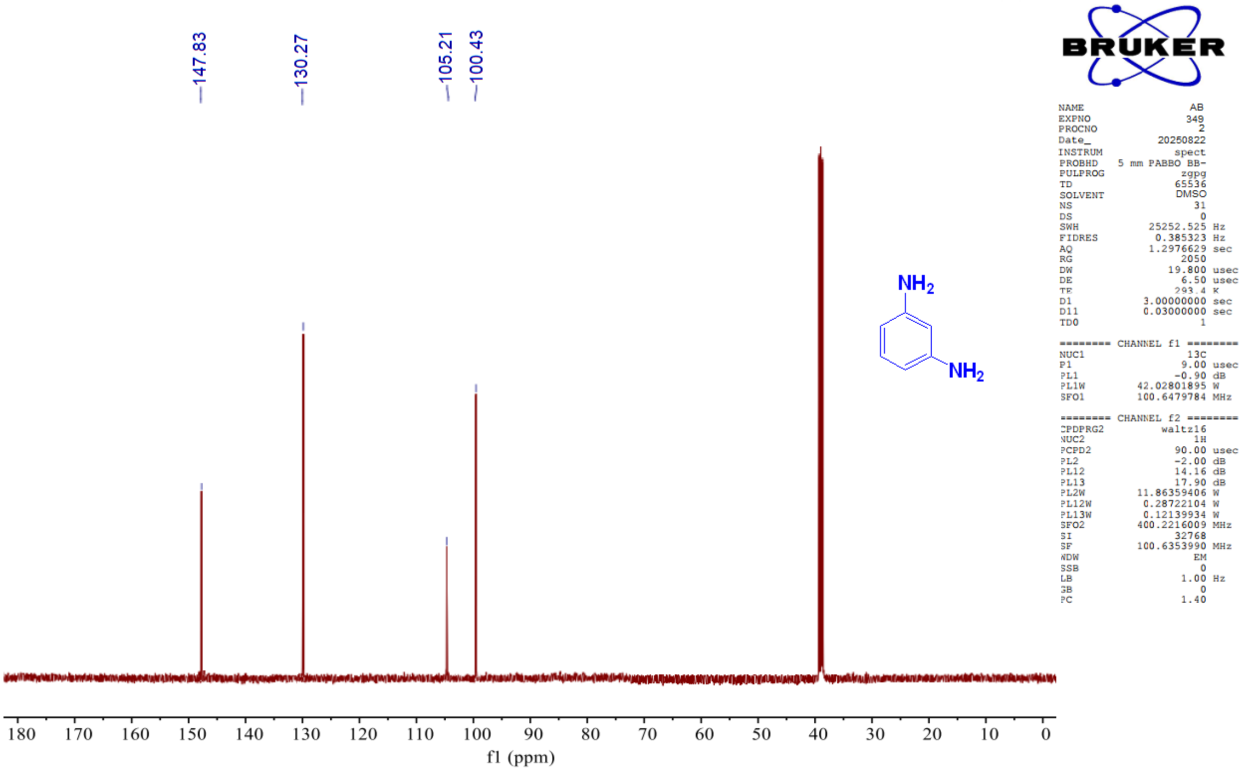

147.83
130.27
105.21
100.43
BRUKER
NAME AB
EXPNO 349
PROCNO 2
Date_ 20250822
INSTRUM spect
PROBHD 5 mm PABBO BB-
PULPROG zgpg
TD 65536
SOLVENT DMSO
NS 31
DS 0
SWH 25252.525 Hz
FIDRES 0.385323 Hz
AQ 1.2976629 sec
RG 2050
DW 19.800 usec
DE 6.50 usec
TE 293.4 K
D1 3.00000000 sec
D11 0.03000000 sec
TD0 1
======= CHANNEL f1 =======
NUC1 13C
P1 9.00 usec
PL1 -0.90 dB
PL1W 42.02801895 W
SFO1 100.6479784 MHz
======= CHANNEL f2 =======
CPDPRG2 waltz16
NUC2 1H
PCPD2 90.00 usec
PL2 -2.00 dB
PL12 14.16 dB
PL13 17.90 dB
PL2W 11.86359406 W
PL12W 0.28722104 W
PL13W 0.12139934 W
SFO2 400.2216009 MHz
SI 32768
SF 100.6353990 MHz
WDW EM
SSB 0
LB 1.00 Hz
GB 0
PC 1.40
NH_2
NH_2
180 170 160 150 140 130 120 110 100 90 80 70 60 50 40 30 20 10 0
f1 (ppm)

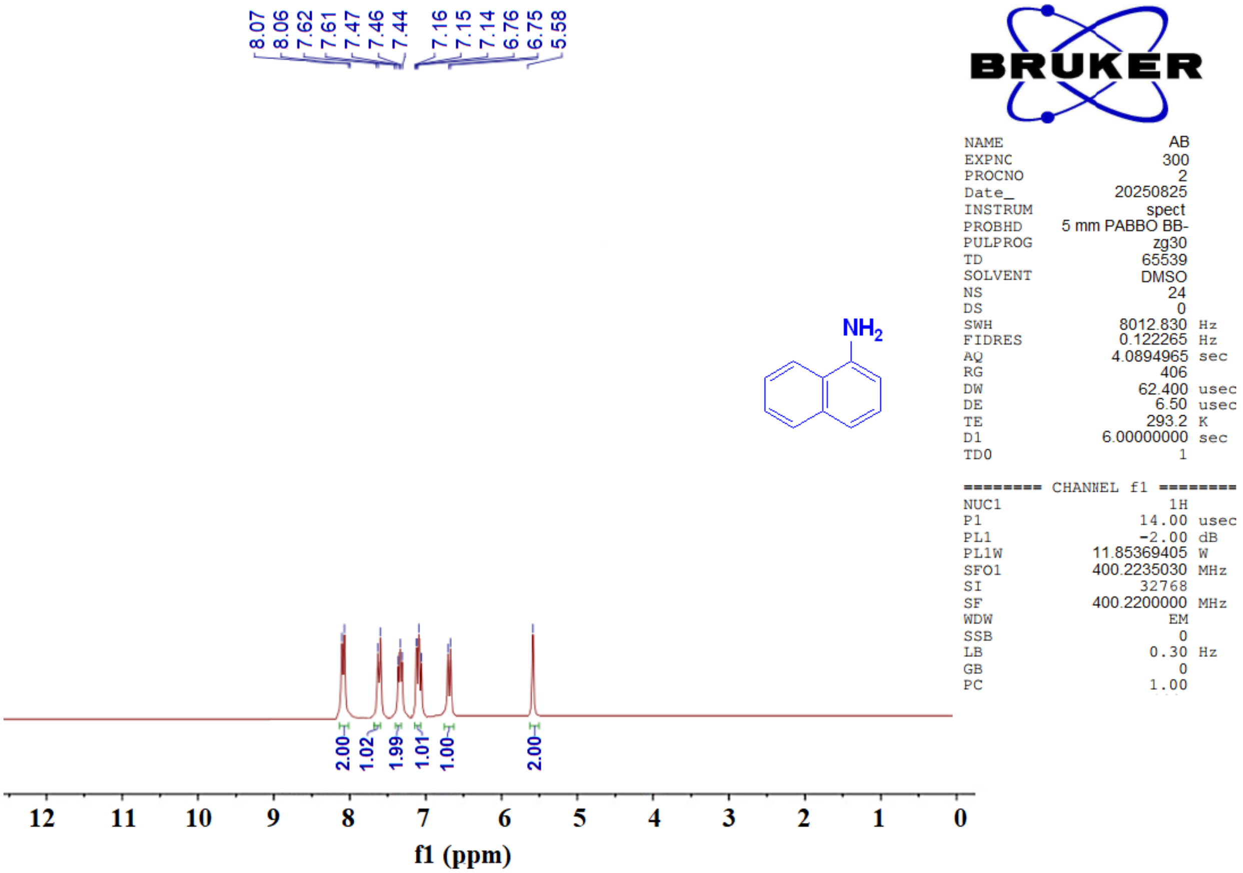
8.07
8.06
7.62
7.61
7.47
7.46
7.44
7.16
7.15
7.14
6.76
6.75
5.58
BRUKER
NAME AB
EXPNC 300
PROCNO 2
Date_ 20250825
INSTRUM spect
PROBHD 5 mm PABBO BB-
PULPROG zg30
TD 65539
SOLVENT DMSO
NS 24
DS 0
SWH 8012.830 Hz
FIDRES 0.122265 Hz
AQ 4.0894965 sec
RG 406
DW 62.400 usec
DE 6.50 usec
TE 293.2 K
D1 6.00000000 sec
TD0 1
======== CHANNEL f1 ========
NUC1 1H
P1 14.00 usec
PL1 -2.00 dB
PL1W 11.85369405 W
SFO1 400.2235030 MHz
SI 32768
SF 400.2200000 MHz
WDW EM
SSB 0
LB 0.30 Hz
GB 0
PC 1.00
NH_2
2.00
1.02
1.99
1.01
1.00
2.00
12
11
10
9
8
7
6
5
4
3
2
1
0
f1 (ppm)

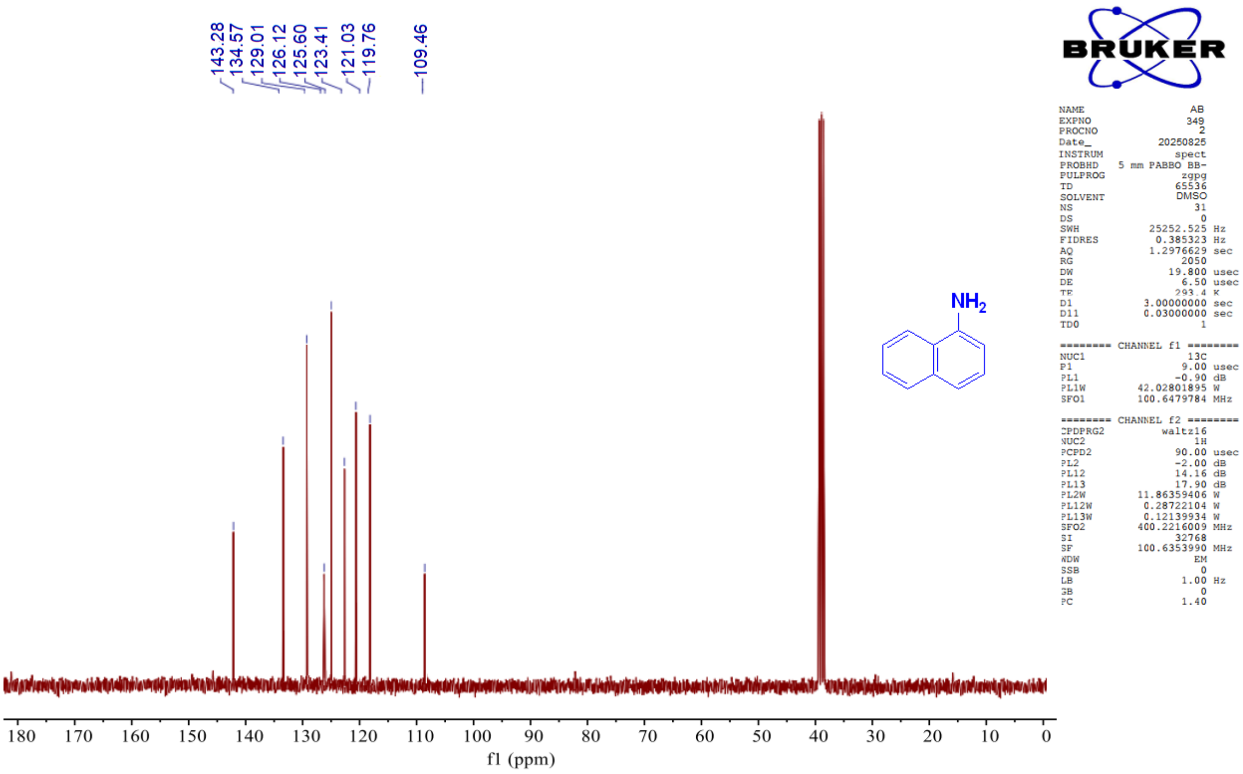
143.28
134.57
129.01
126.12
125.60
123.41
121.03
119.76
109.46
BRUKER
NAME AB
EXPNO 349
PROCNO 2
Date_ 20250825
INSTRUM spect
PROBHD 5 mm PABBO BB-
PULPROG zgpg
TD 65536
SOLVENT DMSO
NS 31
DS 0
SWH 25252.525 Hz
FIDRES 0.385323 Hz
AQ 1.2976629 sec
RG 2050
DW 19.800 usec
DE 6.50 usec
TE 293.4 K
D1 3.00000000 sec
D11 0.03000000 sec
TD0 1
======== CHANNEL f1 ========
NUC1 13C
P1 9.00 usec
PL1 -0.90 dB
PL1W 42.02801895 W
SFO1 100.6479784 MHz
======== CHANNEL f2 ========
CPDPRG2 waltz16
NUC2 1H
PCPD2 90.00 usec
PL2 -2.00 dB
PL12 14.16 dB
PL13 17.90 dB
PL2W 11.86359406 W
PL12W 0.28722104 W
PL13W 0.12139934 W
SFO2 400.2216009 MHz
SI 32768
SF 100.6353990 MHz
WDW EM
SSB 0
LB 1.00 Hz
GB 0
PC 1.40
NH_2
180
170
160
150
140
130
120
110
100
90
80
70
60
50
40
30
20
10
0
f1 (ppm)

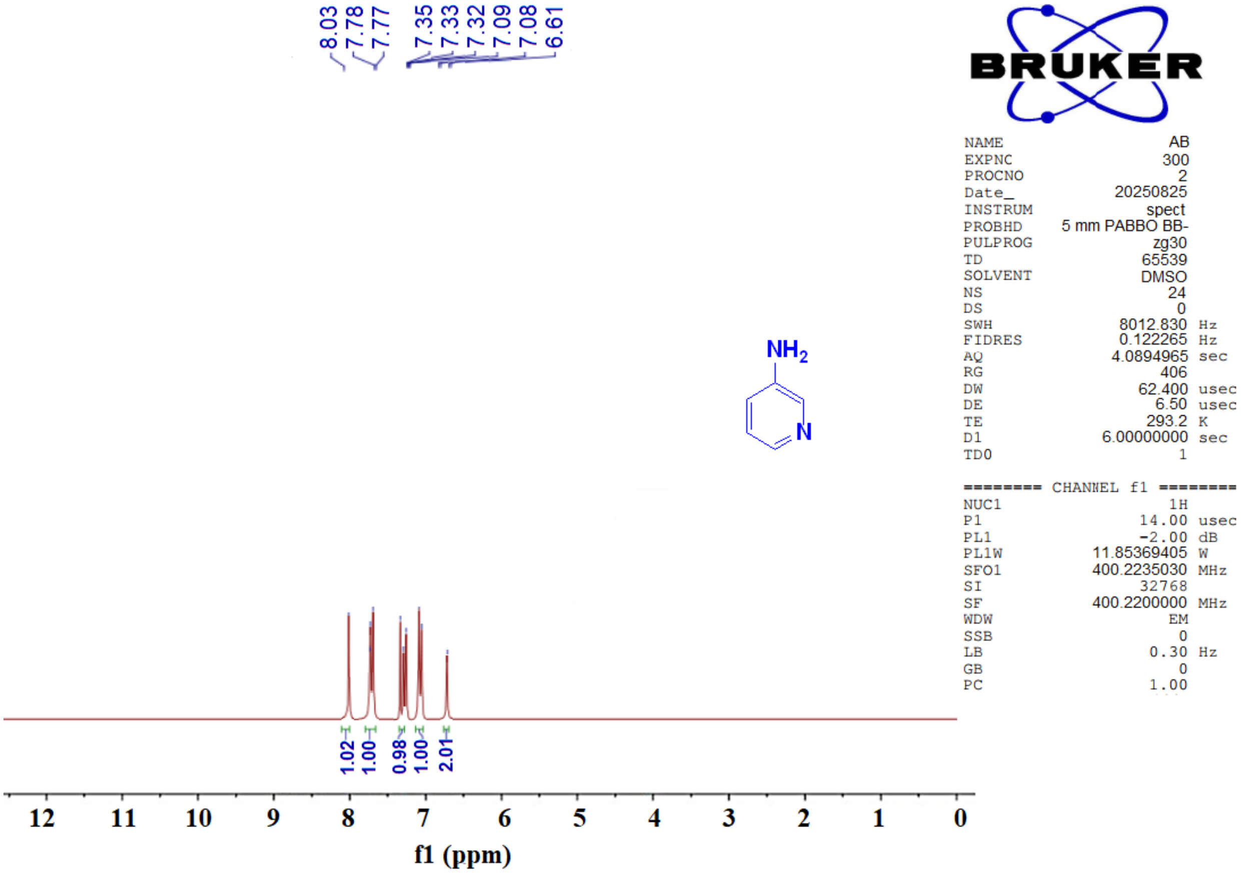
8.03
7.78
7.77
7.35
7.33
7.32
7.09
7.08
6.61
NH2
N
BRUKER
NAME AB
EXPNC 300
PROCNO 2
Date_ 20250825
INSTRUM spect
PROBHD 5 mm PABBO BB-
PULPROG zg30
TD 65539
SOLVENT DMSO
NS 24
DS 0
SWH 8012.830 Hz
FIDRES 0.122265 Hz
AQ 4.0894965 sec
RG 406
DW 62.400 usec
DE 6.50 usec
TE 293.2 K
D1 6.00000000 sec
TD0 1
======== CHANNEL f1 ========
NUC1 1H
P1 14.00 usec
PL1 -2.00 dB
PL1W 11.85369405 W
SFO1 400.2235030 MHz
SI 32768
SF 400.2200000 MHz
WDW EM
SSB 0
LB 0.30 Hz
GB 0
PC 1.00
1.02
1.00
0.98
1.00
2.01
12 11 10 9 8 7 6 5 4 3 2 1 0
f1 (ppm)

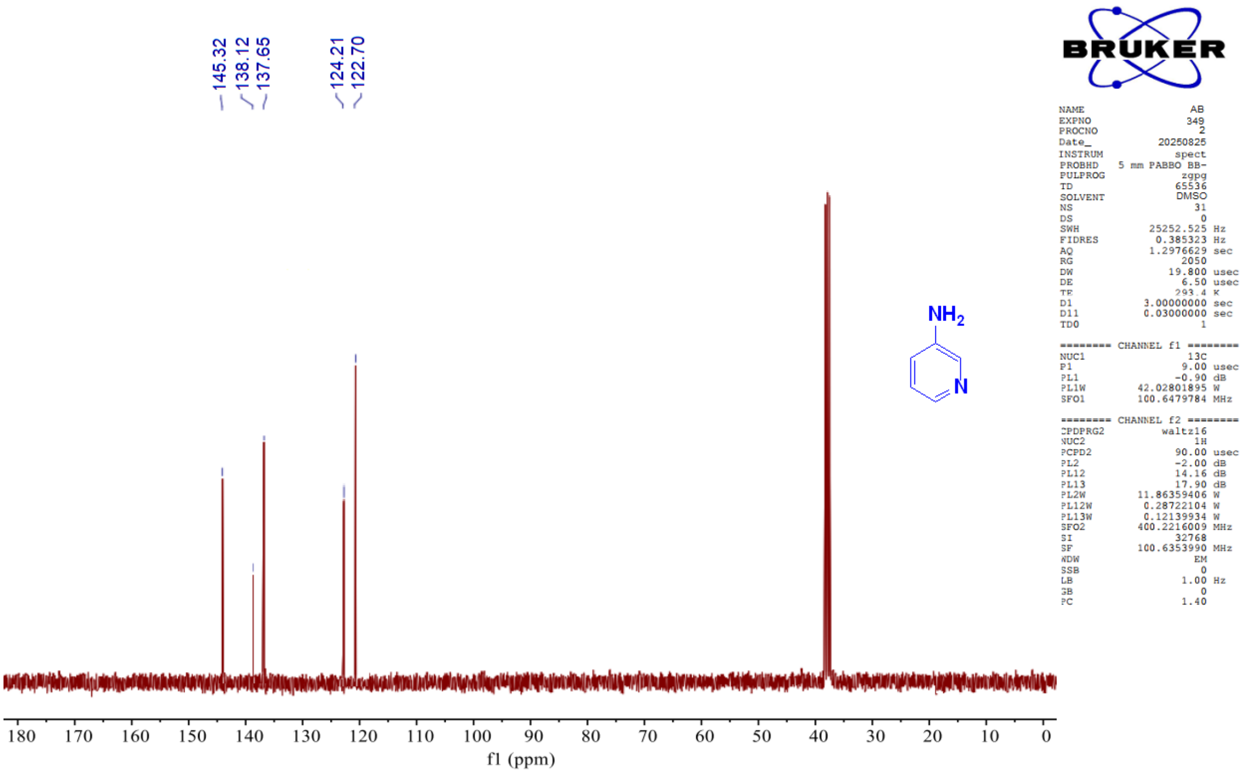
145.32
138.12
137.65
124.21
122.70
NH2
N
BRUKER
NAME AB
EXPNO 349
PROCNO 2
Date_ 20250825
INSTRUM spect
PROBHD 5 mm PABBO BB-
PULPROG zgpg
TD 65536
SOLVENT DMSO
NS 31
DS 0
SWH 25252.525 Hz
FIDRES 0.385323 Hz
AQ 1.2976629 sec
RG 2050
DW 19.800 usec
DE 6.50 usec
TE 293.4 K
D1 3.00000000 sec
D11 0.03000000 sec
TD0 1
======== CHANNEL f1 ========
NUC1 13C
P1 9.00 usec
PL1 -0.90 dB
PL1W 42.02801895 W
SFO1 100.6479784 MHz
======== CHANNEL f2 ========
CPDPRG2 waltz16
NUC2 1H
PCPD2 90.00 usec
PL2 -2.00 dB
PL12 14.16 dB
PL13 17.90 dB
PL2W 11.86359406 W
PL12W 0.28722104 W
PL13W 0.12139934 W
SFO2 400.2216009 MHz
SI 32768
SF 100.6353990 MHz
WDW EM
SSB 0
LB 1.00 Hz
GB 0
PC 1.40
180 170 160 150 140 130 120 110 100 90 80 70 60 50 40 30 20 10 0
f1 (ppm)

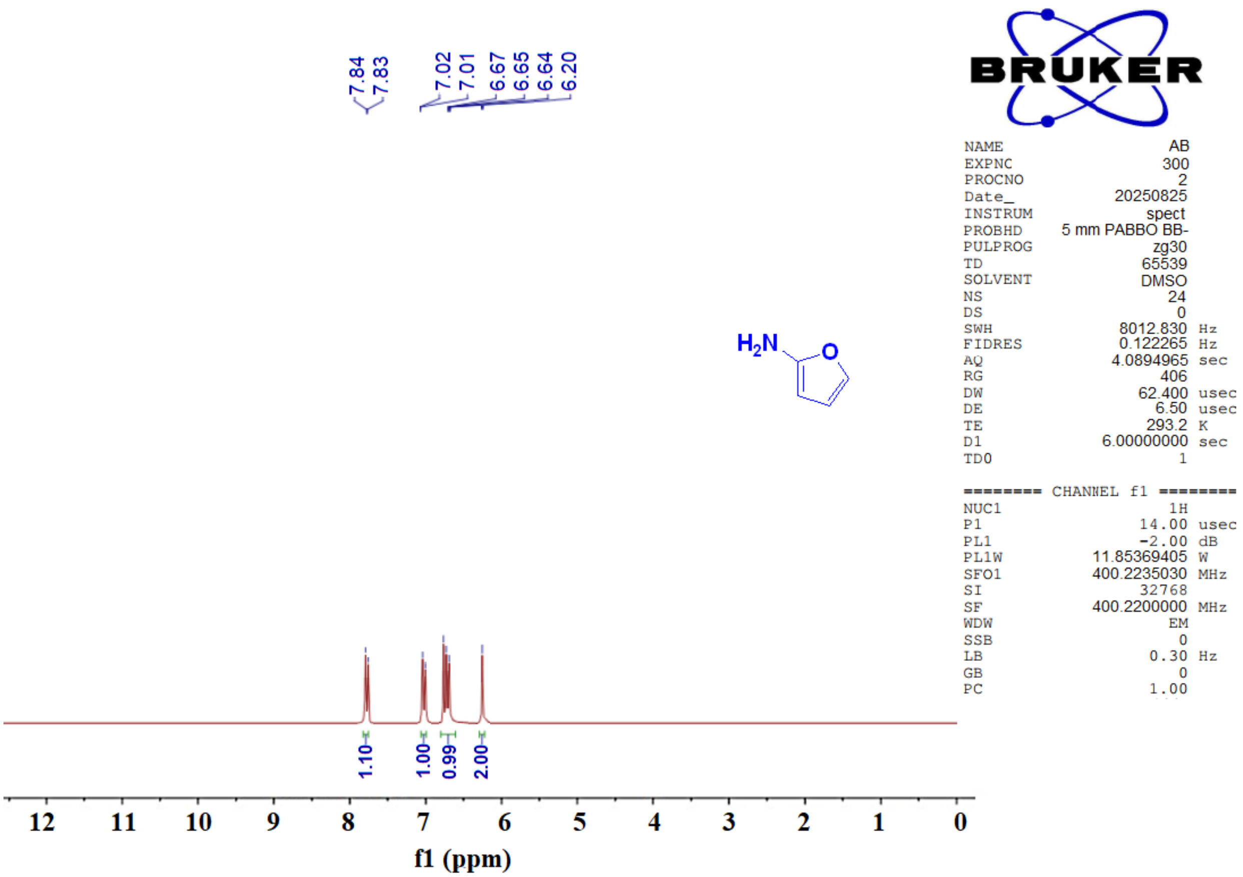

7.84
7.83
7.02
7.01
6.67
6.65
6.64
6.20
BRUKER
NAME AB
EXPNC 300
PROCNO 2
Date_ 20250825
INSTRUM spect
PROBHD 5 mm PABBO BB-
PULPROG zg30
TD 65539
SOLVENT DMSO
NS 24
DS 0
SWH 8012.830 Hz
FIDRES 0.122265 Hz
AQ 4.0894965 sec
RG 406
DW 62.400 usec
DE 6.50 usec
TE 293.2 K
D1 6.00000000 sec
TD0 1
======== CHANNEL f1 ========
NUC1 1H
P1 14.00 usec
PL1 -2.00 dB
PL1W 11.85369405 W
SFO1 400.2235030 MHz
SI 32768
SF 400.2200000 MHz
WDW EM
SSB 0
LB 0.30 Hz
GB 0
PC 1.00
H_2N
O
1.10
1.00
0.99
2.00
12 11 10 9 8 7 6 5 4 3 2 1 0
f1 (ppm)

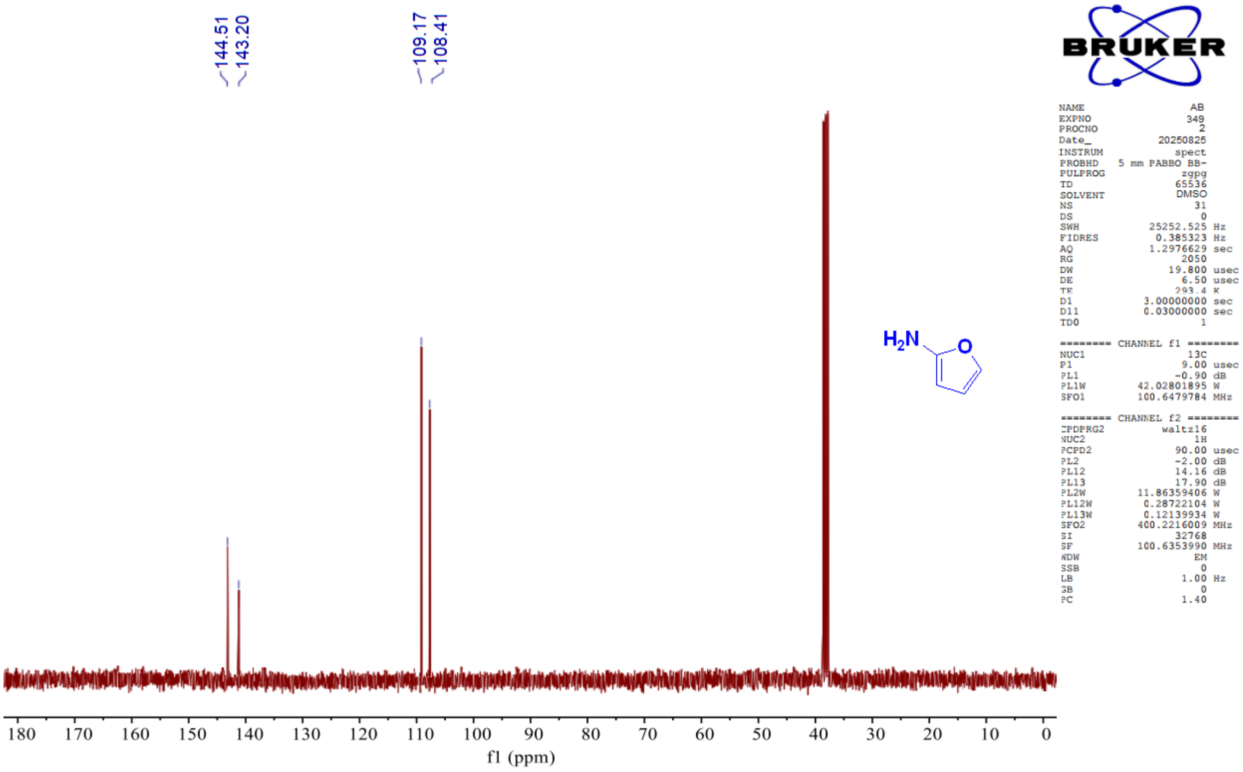

144.51
143.20
109.17
108.41
BRUKER
NAME AB
EXPNO 349
PROCNO 2
Date_ 20250825
INSTRUM spect
PROBHD 5 mm PABBO BB-
PULPROG zgpg
TD 65536
SOLVENT DMSO
NS 31
DS 0
SWH 25252.525 Hz
FIDRES 0.385323 Hz
AQ 1.2976629 sec
RG 2050
DW 19.800 usec
DE 6.50 usec
TE 293.4 K
D1 3.00000000 sec
D11 0.03000000 sec
TD0 1
======== CHANNEL f1 ========
NUC1 13C
P1 9.00 usec
PL1 -0.90 dB
PL1W 42.02801895 W
SFO1 100.6479784 MHz
======== CHANNEL f2 ========
CPDPRG2 waltz16
NUC2 1H
PCPD2 90.00 usec
PL2 -2.00 dB
PL12 14.16 dB
PL13 17.90 dB
PL2W 11.86359406 W
PL12W 0.28722104 W
PL13W 0.12139934 W
SFO2 400.2216009 MHz
SI 32768
SF 100.6353990 MHz
WDW EM
SSB 0
LB 1.00 Hz
GB 0
PC 1.40
H_2N
O
180 170 160 150 140 130 120 110 100 90 80 70 60 50 40 30 20 10 0
f1 (ppm)

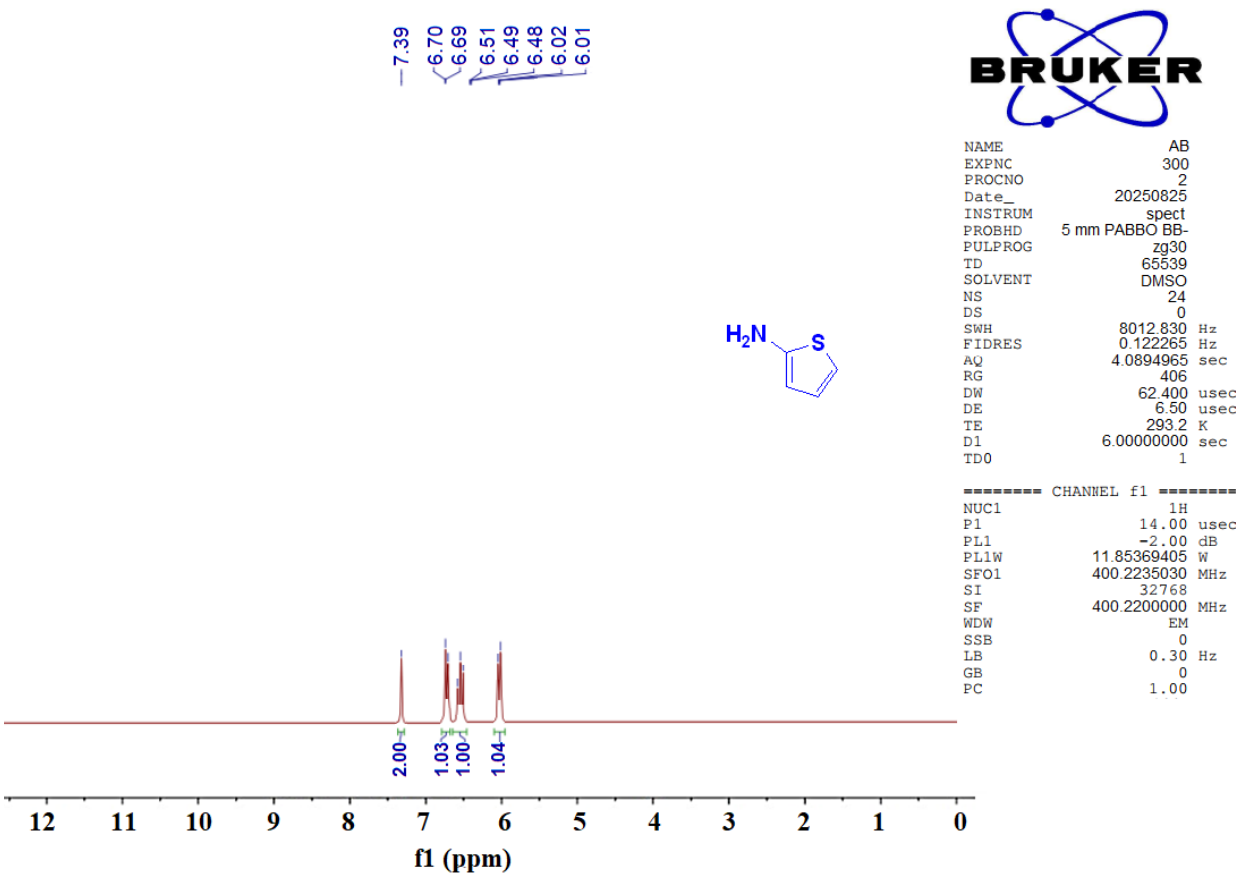
7.39
6.70
6.69
6.51
6.49
6.48
6.02
6.01
NAME AB
EXPNC 300
PROCNO 2
Date_ 20250825
INSTRUM spect
PROBHD 5 mm PABBO BB-
PULPROG zg30
TD 65539
SOLVENT DMSO
NS 24
DS 0
SWH 8012.830 Hz
FIDRES 0.122265 Hz
AQ 4.0894965 sec
RG 406
DW 62.400 usec
DE 6.50 usec
TE 293.2 K
D1 6.00000000 sec
TD0 1
======== CHANNEL f1 ========
NUC1 1H
P1 14.00 usec
PL1 -2.00 dB
PL1W 11.85369405 W
SFO1 400.2235030 MHz
SI 32768
SF 400.2200000 MHz
WDW EM
SSB 0
LB 0.30 Hz
GB 0
PC 1.00
H2N
S
2.00
1.03
1.00
1.04
12
11
10
9
8
7
6
5
4
3
2
1
0
f1 (ppm)

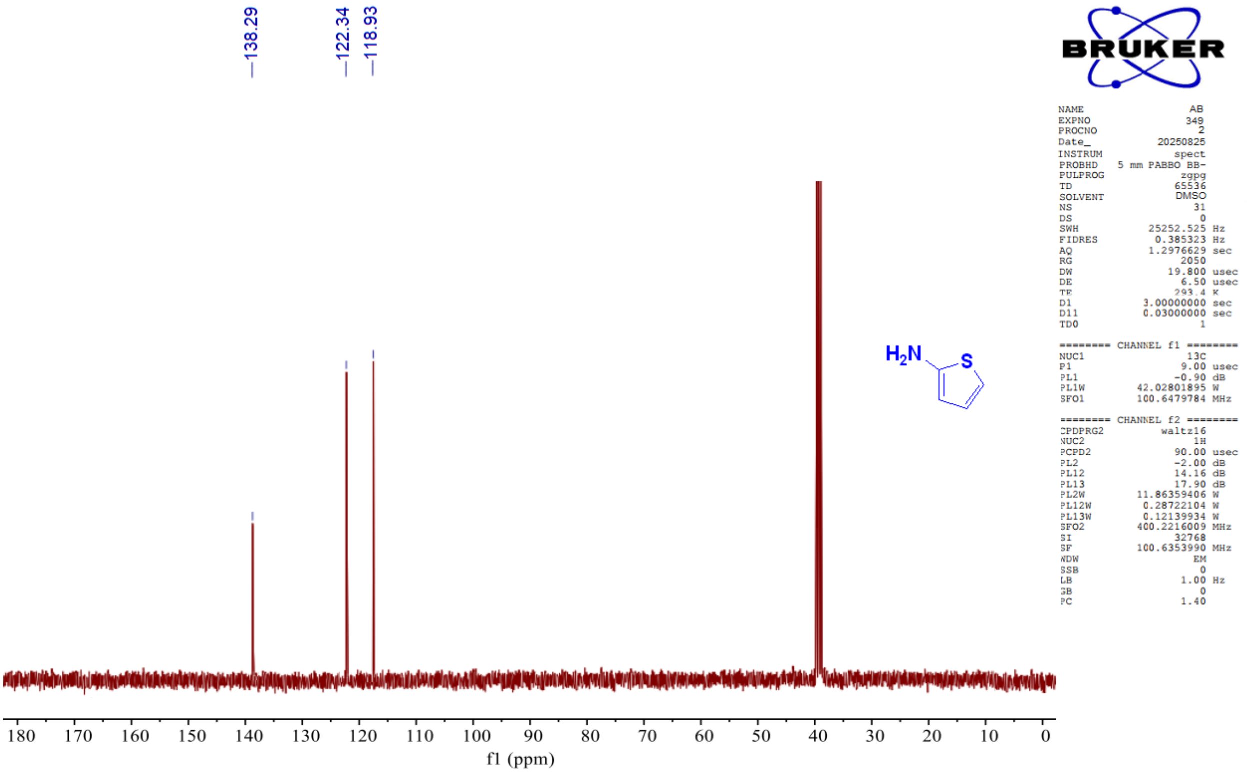
138.29
122.34
118.93
NAME AB
EXPNO 349
PROCNO 2
Date_ 20250825
INSTRUM spect
PROBHD 5 mm PABBO BB-
PULPROG zgpg
TD 65536
SOLVENT DMSO
NS 31
DS 0
SWH 25252.525 Hz
FIDRES 0.385323 Hz
AQ 1.2976629 sec
RG 2050
DW 19.800 usec
DE 6.50 usec
TE 293.4 K
D1 3.00000000 sec
D11 0.03000000 sec
TD0 1
======== CHANNEL f1 ========
NUC1 13C
P1 9.00 usec
PL1 -0.90 dB
PL1W 42.02801895 W
SFO1 100.6479784 MHz
======== CHANNEL f2 ========
CPDPRG2 waltz16
NUC2 1H
PCPD2 90.00 usec
PL2 -2.00 dB
PL12 14.16 dB
PL13 17.90 dB
PL2W 11.86359406 W
PL12W 0.28722104 W
PL13W 0.12139934 W
SFO2 400.2216009 MHz
SI 32768
SF 100.6353990 MHz
WDW EM
SSB 0
LB 1.00 Hz
GB 0
PC 1.40
H2N
S
180
170
160
150
140
130
120
110
100
90
80
70
60
50
40
30
20
10
0
f1 (ppm)

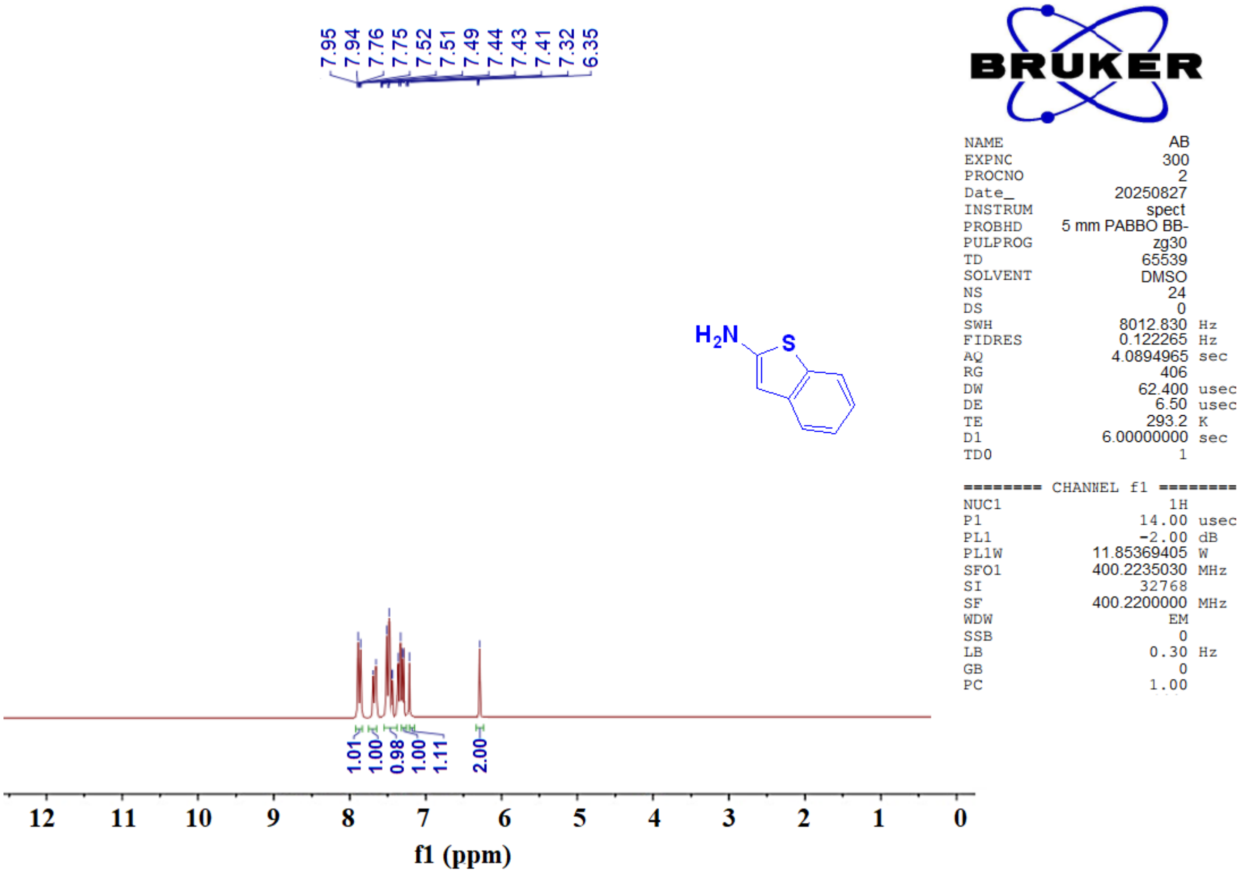

7.95
7.94
7.76
7.75
7.52
7.51
7.49
7.44
7.43
7.41
7.32
6.35
H_2N
S
NAME AB
EXPNC 300
PROCNO 2
Date_ 20250827
INSTRUM spect
PROBHD 5 mm PABBO BB-
PULPROG zg30
TD 65539
SOLVENT DMSO
NS 24
DS 0
SWH 8012.830 Hz
FIDRES 0.122265 Hz
AQ 4.0894965 sec
RG 406
DW 62.400 usec
DE 6.50 usec
TE 293.2 K
D1 6.00000000 sec
TD0 1
======== CHANNEL f1 ========
NUC1 1H
P1 14.00 usec
PL1 -2.00 dB
PL1W 11.85369405 W
SFO1 400.2235030 MHz
SI 32768
SF 400.2200000 MHz
WDW EM
SSB 0
LB 0.30 Hz
GB 0
PC 1.00
1.01
1.00
0.98
1.00
1.11
2.00
12 11 10 9 8 7 6 5 4 3 2 1 0
f1 (ppm)

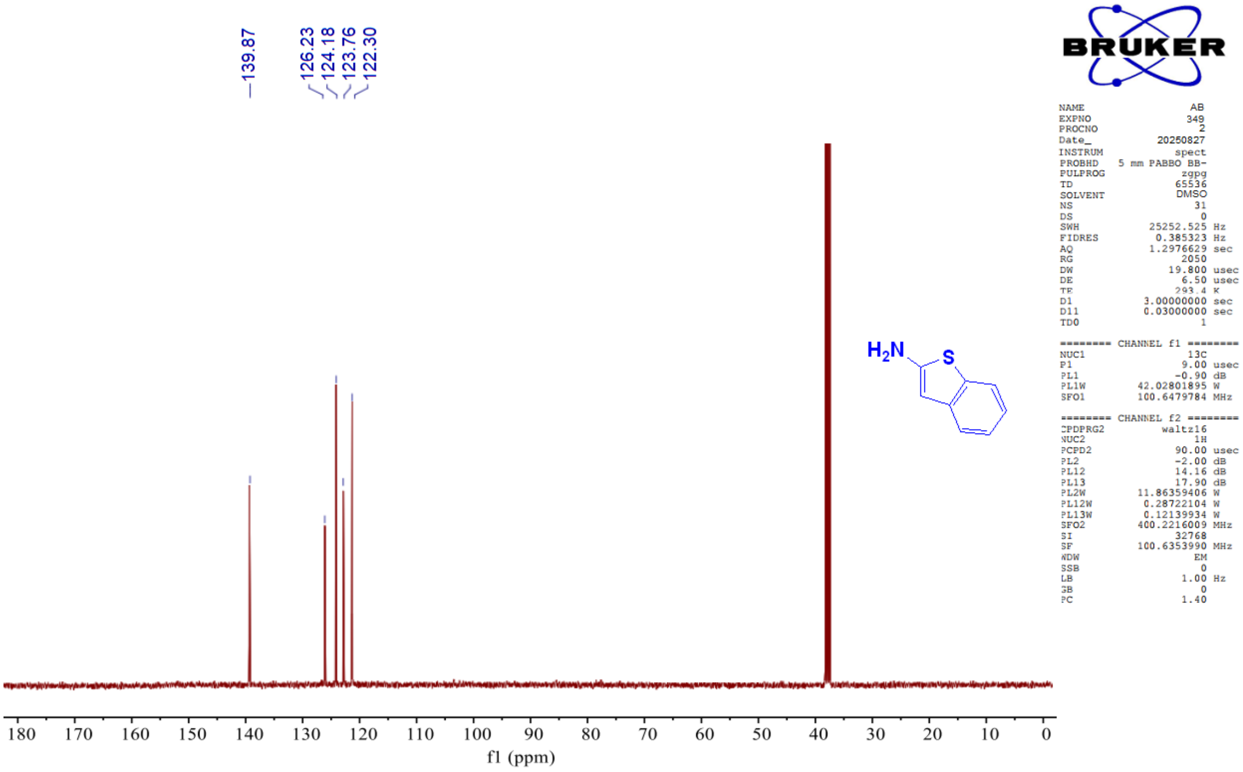

139.87
126.23
124.18
123.76
122.30
H_2N
S
NAME AB
EXPNO 349
PROCNO 2
Date_ 20250827
INSTRUM spect
PROBHD 5 mm PABBO BB-
PULPROG zgpg
TD 65536
SOLVENT DMSO
NS 31
DS 0
SWH 25252.525 Hz
FIDRES 0.385323 Hz
AQ 1.2976629 sec
RG 2050
DW 19.800 usec
DE 6.50 usec
TE 293.4 K
D1 3.00000000 sec
D11 0.03000000 sec
TD0 1
======== CHANNEL f1 ========
NUC1 13C
P1 9.00 usec
PL1 -0.90 dB
PL1W 42.02801895 W
SFO1 100.6479784 MHz
======== CHANNEL f2 ========
CPDPRG2 waltz16
NUC2 1H
PCPD2 90.00 usec
PL2 -2.00 dB
PL12 14.16 dB
PL13 17.90 dB
PL2W 11.86359406 W
PL12W 0.28722104 W
PL13W 0.12139934 W
SFO2 400.2216009 MHz
SI 32768
SF 100.6353990 MHz
WDW EM
SSB 0
LB 1.00 Hz
GB 0
PC 1.40
180 170 160 150 140 130 120 110 100 90 80 70 60 50 40 30 20 10 0
f1 (ppm)

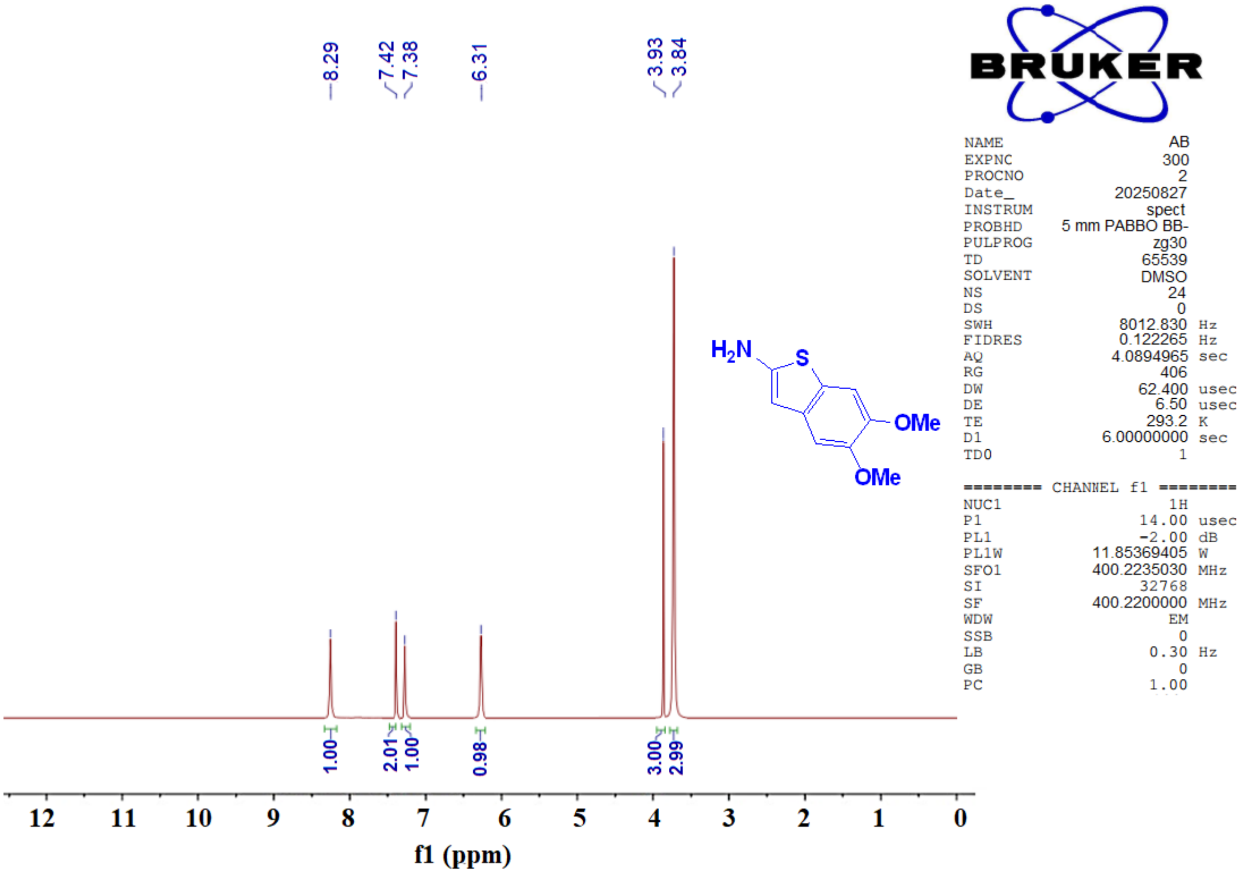
8.29
7.42
7.38
6.31
3.93
3.84
H2N
S
OMe
OMe
1.00
2.01
1.00
0.98
3.00
2.99
12 11 10 9 8 7 6 5 4 3 2 1 0
f1 (ppm)
BRUKER
NAME AB
EXPNC 300
PROCNO 2
Date_ 20250827
INSTRUM spect
PROBHD 5 mm PABBO BB-
PULPROG zg30
TD 65539
SOLVENT DMSO
NS 24
DS 0
SWH 8012.830 Hz
FIDRES 0.122265 Hz
AQ 4.0894965 sec
RG 406
DW 62.400 usec
DE 6.50 usec
TE 293.2 K
D1 6.00000000 sec
TD0 1
======== CHANNEL f1 ========
NUC1 1H
P1 14.00 usec
PL1 -2.00 dB
PL1W 11.85369405 W
SFO1 400.2235030 MHz
SI 32768
SF 400.2200000 MHz
WDW EM
SSB 0
LB 0.30 Hz
GB 0
PC 1.00

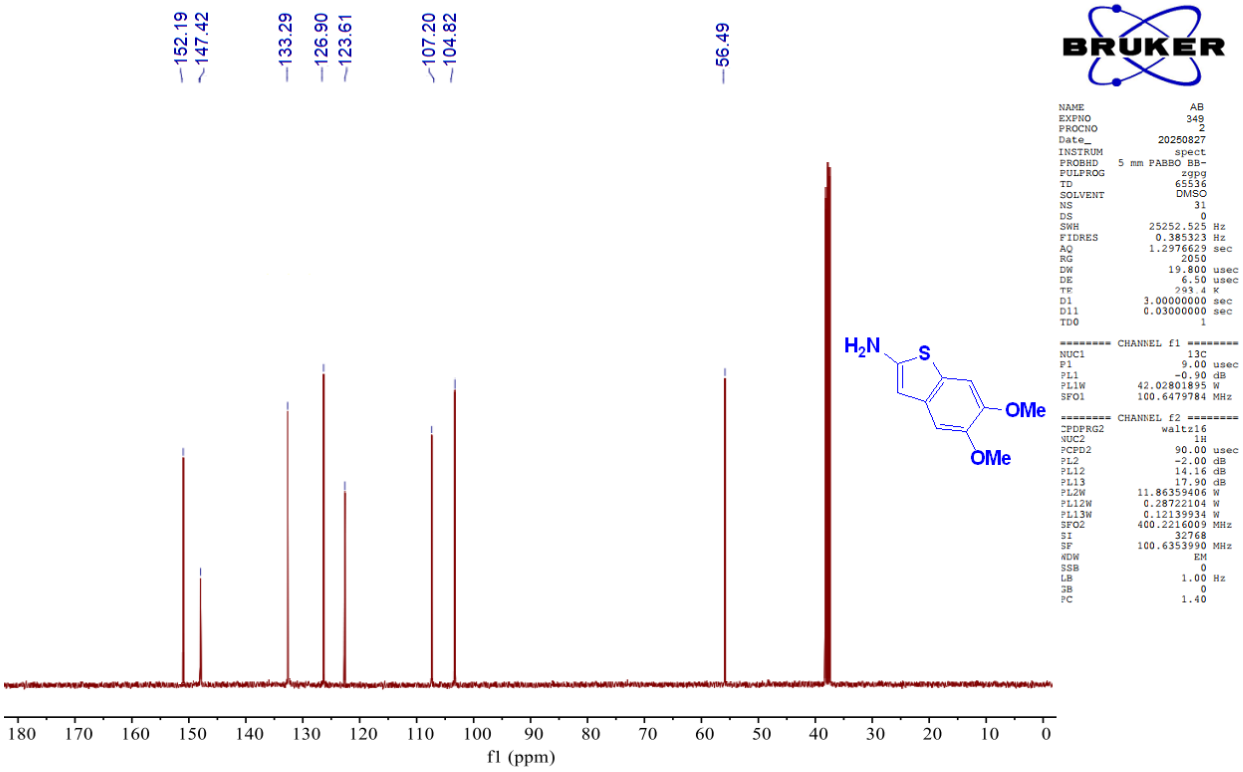
152.19
147.42
133.29
126.90
123.61
107.20
104.82
56.49
H2N
S
OMe
OMe
180 170 160 150 140 130 120 110 100 90 80 70 60 50 40 30 20 10 0
f1 (ppm)
BRUKER
NAME AB
EXPNO 349
PROCNO 2
Date_ 20250827
INSTRUM spect
PROBHD 5 mm PABBO BB-
PULPROG zgpg
TD 65536
SOLVENT DMSO
NS 31
DS 0
SWH 25252.525 Hz
FIDRES 0.385323 Hz
AQ 1.2976629 sec
RG 2050
DW 19.800 usec
DE 6.50 usec
TE 293.4 K
D1 3.00000000 sec
D11 0.03000000 sec
TD0 1
======== CHANNEL f1 ========
NUC1 13C
P1 9.00 usec
PL1 -0.90 dB
PL1W 42.02801895 W
SFO1 100.6479784 MHz
======== CHANNEL f2 ========
CPDPRG2 waltz16
NUC2 1H
PCPD2 90.00 usec
PL2 -2.00 dB
PL12 14.16 dB
PL13 17.90 dB
PL2W 11.86359406 W
PL12W 0.28722104 W
PL13W 0.12139934 W
SFO2 400.2216009 MHz
SI 32768
SF 100.6353990 MHz
WDW EM
SSB 0
LB 1.00 Hz
GB 0
PC 1.40

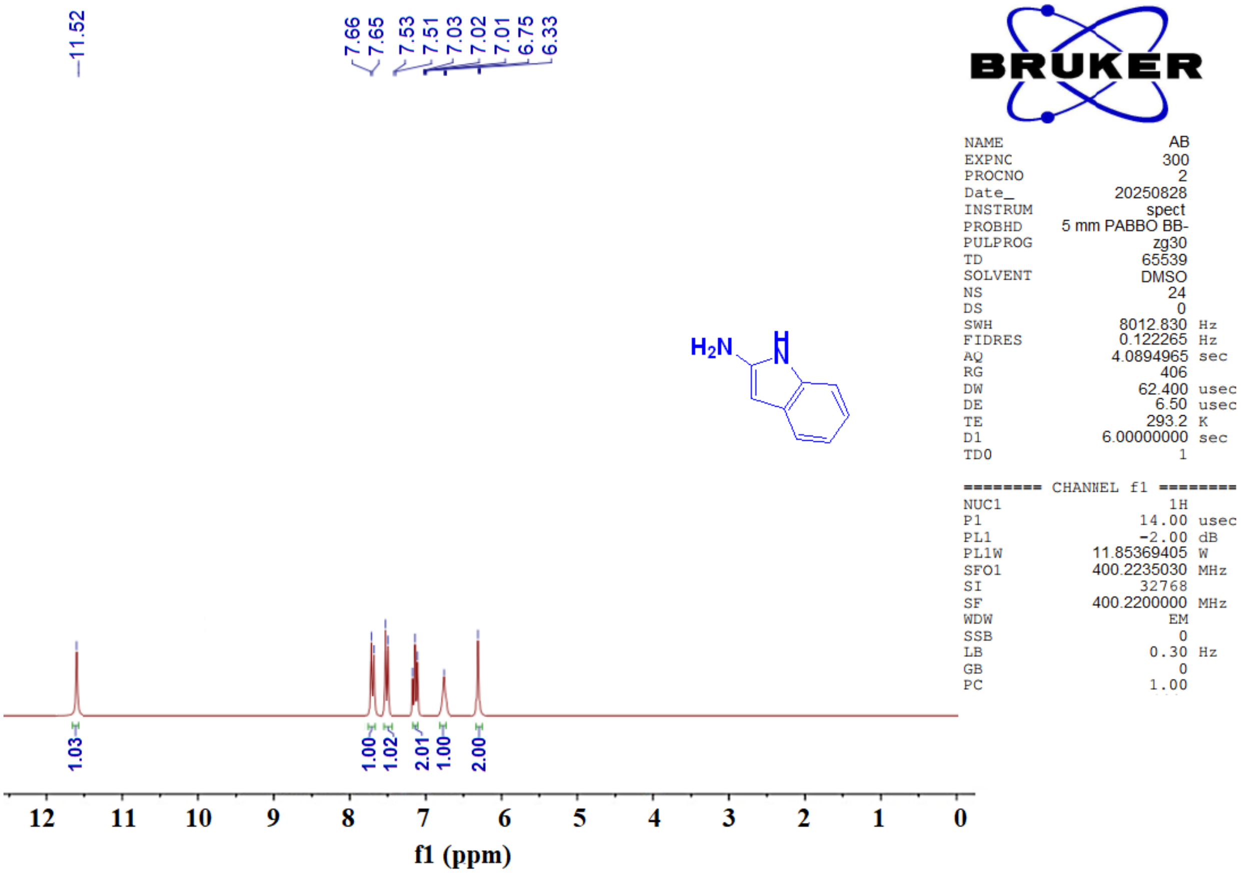
11.52
7.66
7.65
7.53
7.51
7.03
7.02
7.01
6.75
6.33
H₂N
H
N
1.03
1.00
1.02
2.01
1.00
2.00
12 11 10 9 8 7 6 5 4 3 2 1 0
f1 (ppm)
NAME AB
EXPNC 300
PROCNO 2
Date_ 20250828
INSTRUM spect
PROBHD 5 mm PABBO BB-
PULPROG zg30
TD 65539
SOLVENT DMSO
NS 24
DS 0
SWH 8012.830 Hz
FIDRES 0.122265 Hz
AQ 4.0894965 sec
RG 406
DW 62.400 usec
DE 6.50 usec
TE 293.2 K
D1 6.00000000 sec
TD0 1
======== CHANNEL f1 ========
NUC1 1H
P1 14.00 usec
PL1 -2.00 dB
PL1W 11.85369405 W
SFO1 400.2235030 MHz
SI 32768
SF 400.2200000 MHz
WDW EM
SSB 0
LB 0.30 Hz
GB 0
PC 1.00

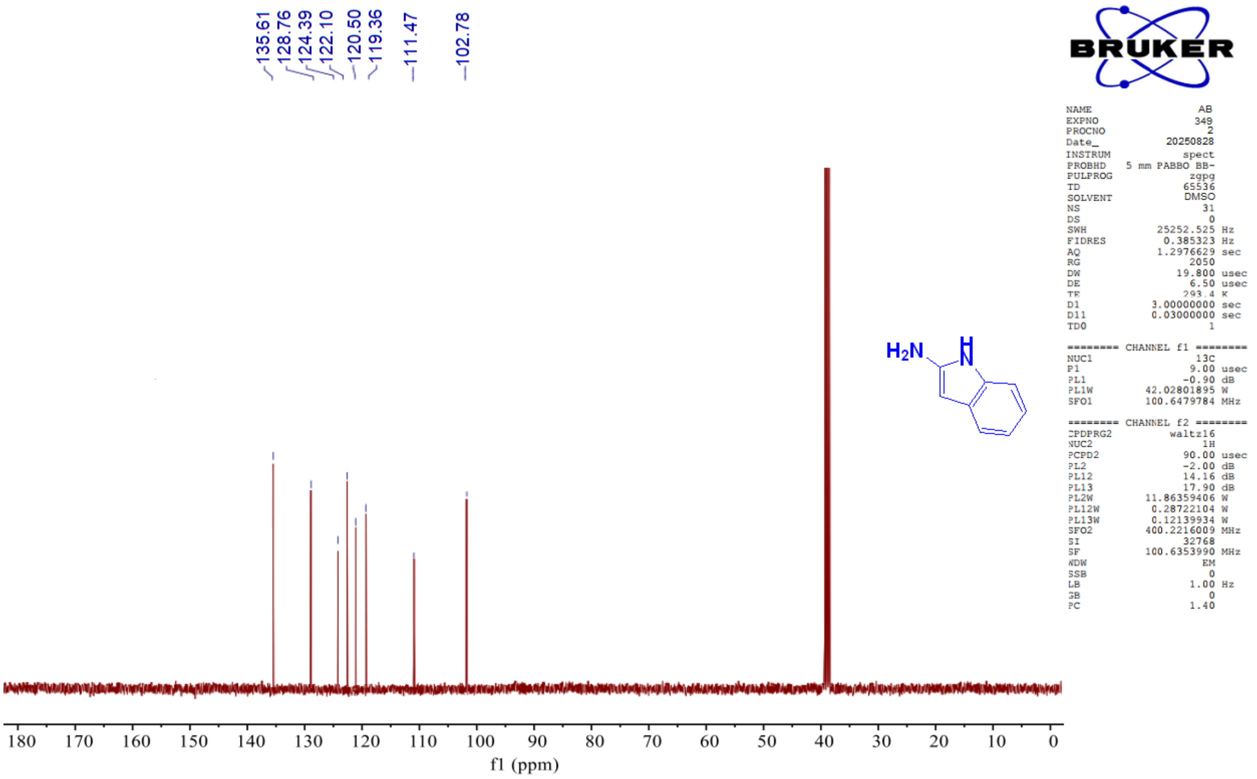
135.61
128.76
124.39
122.10
120.50
119.36
111.47
102.78
H₂N
H
N
180 170 160 150 140 130 120 110 100 90 80 70 60 50 40 30 20 10 0
f1 (ppm)
NAME AB
EXPNO 349
PROCNO 2
Date_ 20250828
INSTRUM spect
PROBHD 5 mm PABBO BB-
PULPROG zgpg
TD 65536
SOLVENT DMSO
NS 31
DS 0
SWH 25252.525 Hz
FIDRES 0.385323 Hz
AQ 1.2976629 sec
RG 2050
DW 19.800 usec
DE 6.50 usec
TE 293.4 K
D1 3.00000000 sec
D11 0.03000000 sec
TD0 1
======== CHANNEL f1 ========
NUC1 13C
P1 9.00 usec
PL1 -0.90 dB
PL1W 42.02801895 W
SFO1 100.6479784 MHz
======== CHANNEL f2 ========
CPDPRG2 waltz16
NUC2 1H
PCPD2 90.00 usec
PL2 -2.00 dB
PL12 14.16 dB
PL13 17.90 dB
PL2W 11.86359406 W
PL12W 0.28722104 W
PL13W 0.12139934 W
SFO2 400.2216009 MHz
SI 32768
SF 100.6353990 MHz
WDW EM
SSB 0
LB 1.00 Hz
GB 0
PC 1.40

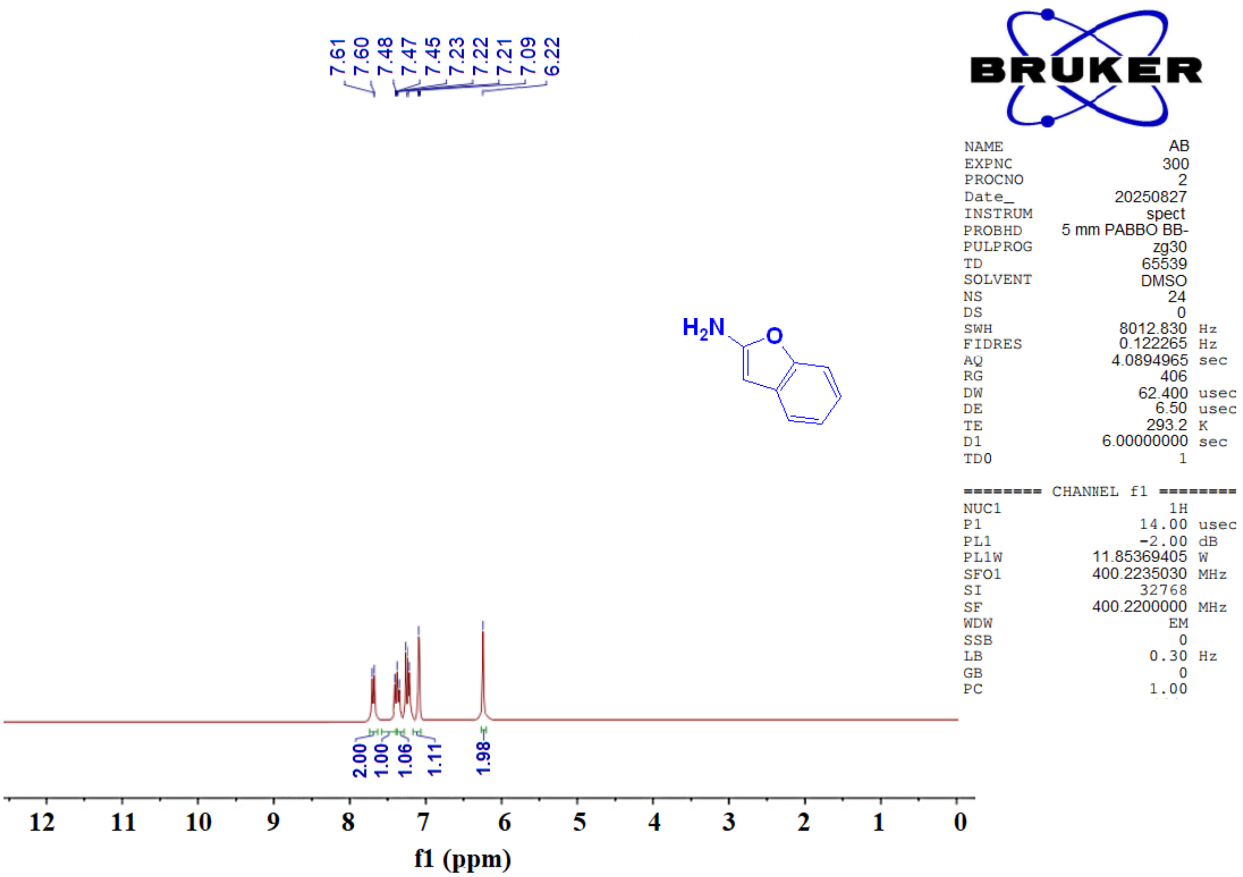
7.61
7.60
7.48
7.47
7.45
7.23
7.22
7.21
7.09
6.22
H_2N
O
2.00
1.00
1.06
1.11
1.98
12
11
10
9
8
7
6
5
4
3
2
1
0
f1 (ppm)
NAME AB
EXPNC 300
PROCNO 2
Date_ 20250827
INSTRUM spect
PROBHD 5 mm PABBO BB-
PULPROG zg30
TD 65539
SOLVENT DMSO
NS 24
DS 0
SWH 8012.830 Hz
FIDRES 0.122265 Hz
AQ 4.0894965 sec
RG 406
DW 62.400 usec
DE 6.50 usec
TE 293.2 K
D1 6.00000000 sec
TD0 1
======== CHANNEL f1 ========
NUC1 1H
P1 14.00 usec
PL1 -2.00 dB
PL1W 11.85369405 W
SFO1 400.2235030 MHz
SI 32768
SF 400.2200000 MHz
WDW EM
SSB 0
LB 0.30 Hz
GB 0
PC 1.00

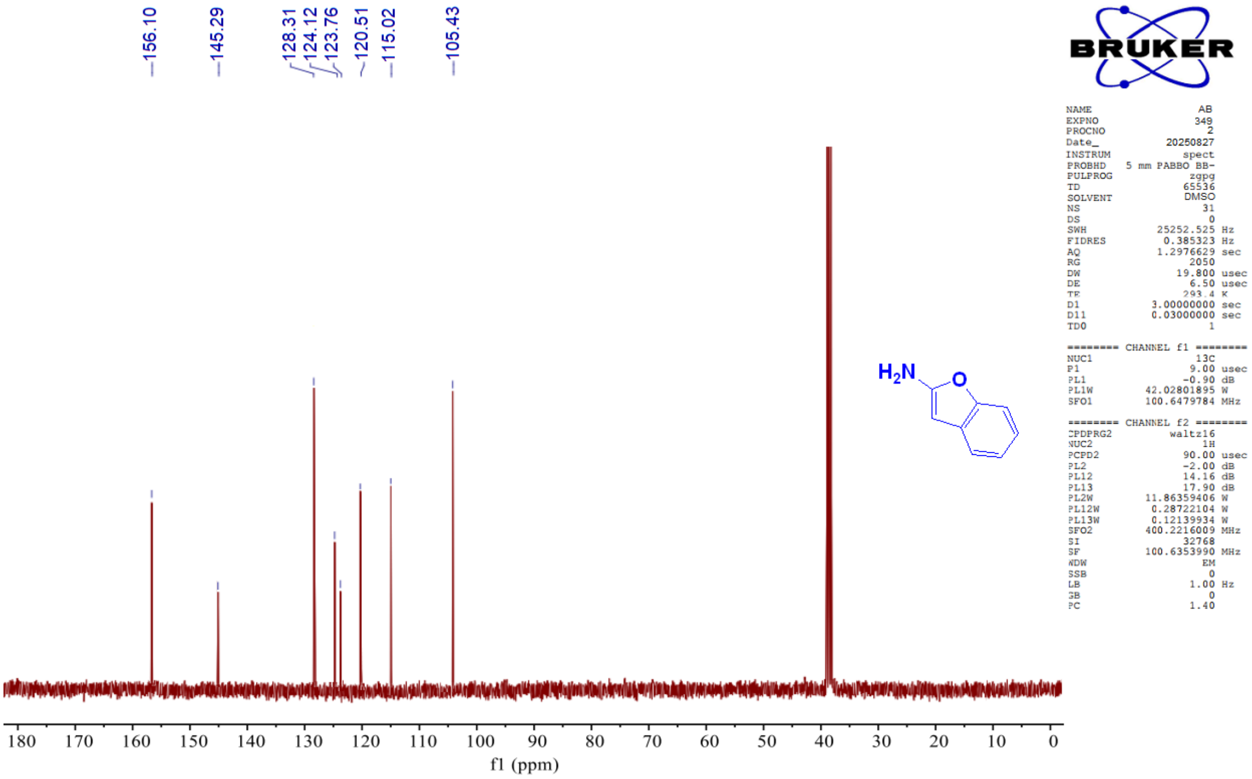
156.10
145.29
128.31
124.12
123.76
120.51
115.02
105.43
H_2N
O
180
170
160
150
140
130
120
110
100
90
80
70
60
50
40
30
20
10
0
f1 (ppm)
NAME AB
EXPNO 349
PROCNO 2
Date_ 20250827
INSTRUM spect
PROBHD 5 mm PABBO BB-
PULPROG zgpg
TD 65536
SOLVENT DMSO
NS 31
DS 0
SWH 25252.525 Hz
FIDRES 0.385323 Hz
AQ 1.2976629 sec
RG 2050
DW 19.800 usec
DE 6.50 usec
TE 293.4 K
D1 3.00000000 sec
D11 0.03000000 sec
TD0 1
======== CHANNEL f1 ========
NUC1 13C
P1 9.00 usec
PL1 -0.90 dB
PL1W 42.02801895 W
SFO1 100.6479784 MHz
======== CHANNEL f2 ========
CPDPRG2 waltz16
NUC2 1H
PCPD2 90.00 usec
PL2 -2.00 dB
PL12 14.16 dB
PL13 17.90 dB
PL2W 11.86359406 W
PL12W 0.28722104 W
PL13W 0.12139934 W
SFO2 400.2216009 MHz
SI 32768
SF 100.6353990 MHz
WDW EM
SSB 0
LB 1.00 Hz
GB 0
PC 1.40

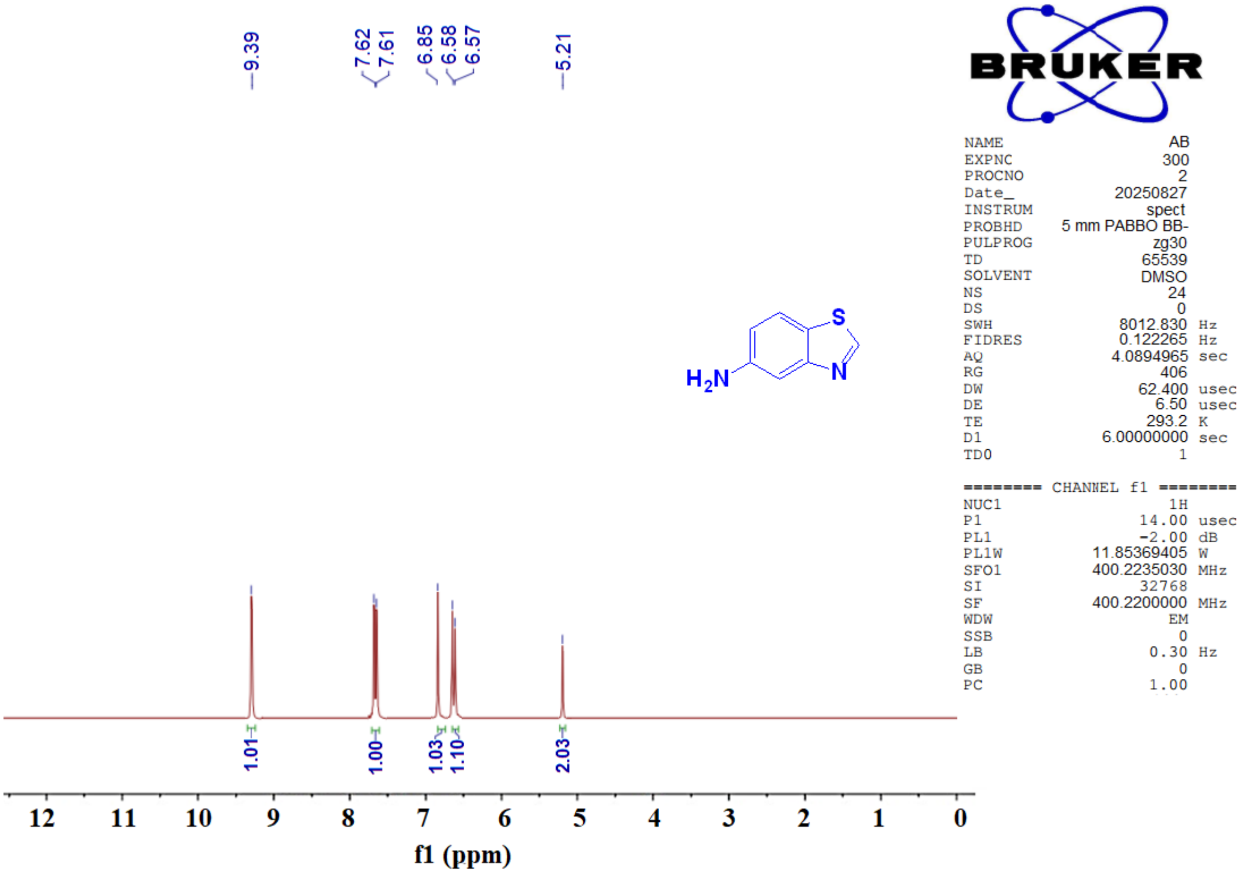
9.39
7.62
7.61
6.85
6.58
6.57
5.21
S
N
H_2N
BRUKER
NAME AB
EXPNC 300
PROCNO 2
Date_ 20250827
INSTRUM spect
PROBHD 5 mm PABBO BB-
PULPROG zg30
TD 65539
SOLVENT DMSO
NS 24
DS 0
SWH 8012.830 Hz
FIDRES 0.122265 Hz
AQ 4.0894965 sec
RG 406
DW 62.400 usec
DE 6.50 usec
TE 293.2 K
D1 6.00000000 sec
TD0 1
======== CHANNEL f1 ========
NUC1 1H
P1 14.00 usec
PL1 -2.00 dB
PL1W 11.85369405 W
SFO1 400.2235030 MHz
SI 32768
SF 400.2200000 MHz
WDW EM
SSB 0
LB 0.30 Hz
GB 0
PC 1.00
1.01
1.00
1.03
1.10
2.03
12
11
10
9
8
7
6
5
4
3
2
1
0
f1 (ppm)

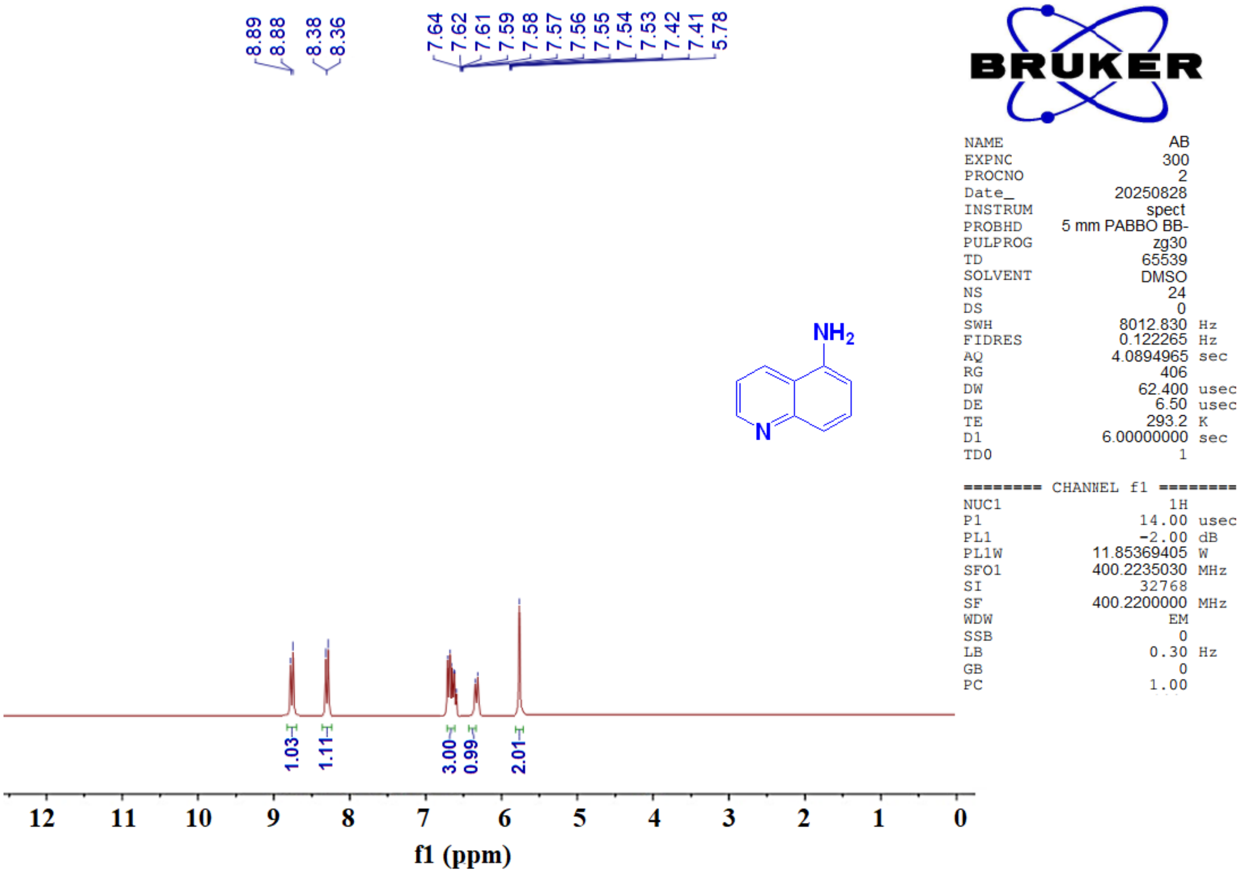
8.89
8.88
8.38
8.36
7.64
7.62
7.61
7.59
7.58
7.57
7.56
7.55
7.54
7.53
7.42
7.41
5.78
BRUKER
NAME AB
EXPNC 300
PROCNO 2
Date_ 20250828
INSTRUM spect
PROBHD 5 mm PABBO BB-
PULPROG zg30
TD 65539
SOLVENT DMSO
NS 24
DS 0
SWH 8012.830 Hz
FIDRES 0.122265 Hz
AQ 4.0894965 sec
RG 406
DW 62.400 usec
DE 6.50 usec
TE 293.2 K
D1 6.00000000 sec
TD0 1
======== CHANNEL f1 ========
NUC1 1H
P1 14.00 usec
PL1 -2.00 dB
PL1W 11.85369405 W
SFO1 400.2235030 MHz
SI 32768
SF 400.2200000 MHz
WDW EM
SSB 0
LB 0.30 Hz
GB 0
PC 1.00
NH_2
N
1.03
1.11
3.00
0.99
2.01
12 11 10 9 8 7 6 5 4 3 2 1 0
f1 (ppm)

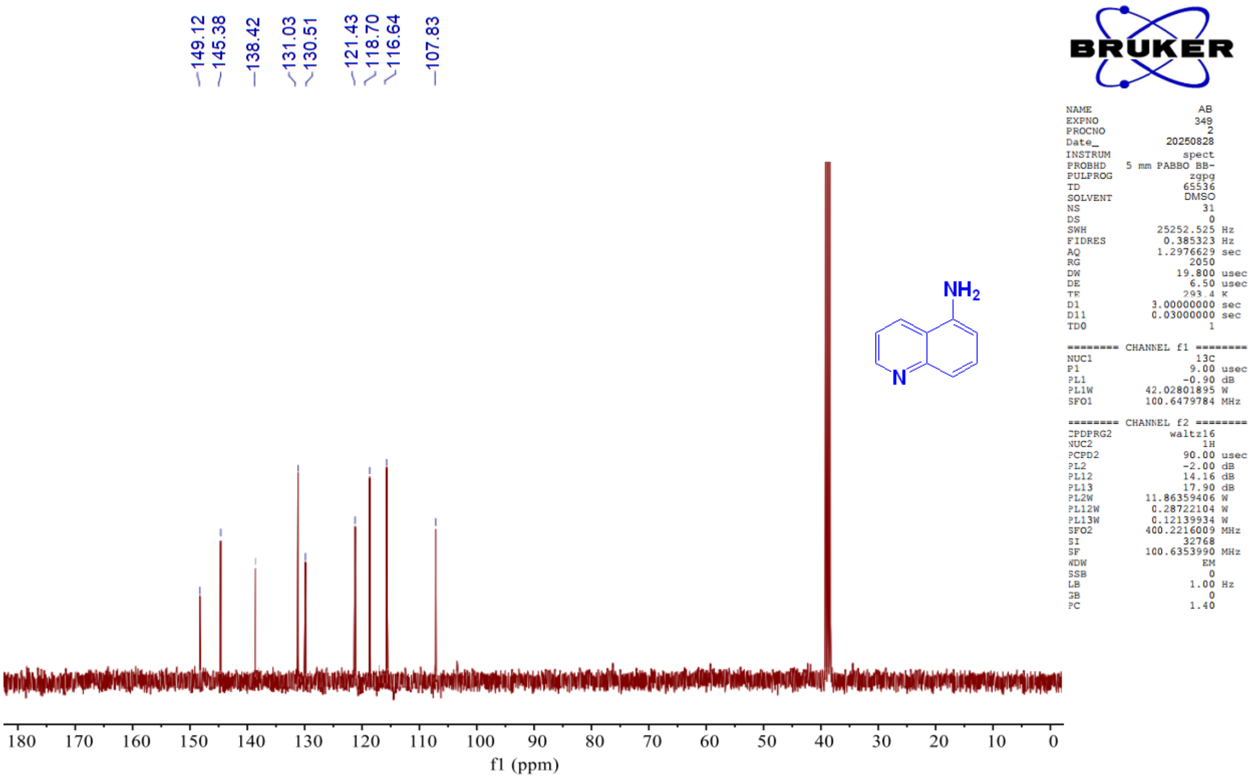
149.12
145.38
138.42
131.03
130.51
121.43
118.70
116.64
107.83
BRUKER
NAME AB
EXPNO 349
PROCNO 2
Date_ 20250828
INSTRUM spect
PROBHD 5 mm PABBO BB-
PULPROG zgpg
TD 65536
SOLVENT DMSO
NS 31
DS 0
SWH 25252.525 Hz
FIDRES 0.385323 Hz
AQ 1.2976629 sec
RG 2050
DW 19.800 usec
DE 6.50 usec
TE 293.4 K
D1 3.00000000 sec
D11 0.03000000 sec
TD0 1
======== CHANNEL f1 ========
NUC1 13C
P1 9.00 usec
PL1 -0.90 dB
PL1W 42.02801895 W
SFO1 100.6479784 MHz
======== CHANNEL f2 ========
CPDPRG2 waltz16
NUC2 1H
PCPD2 90.00 usec
PL2 -2.00 dB
PL12 14.16 dB
PL13 17.90 dB
PL2W 11.86359406 W
PL12W 0.28722104 W
PL13W 0.12139934 W
SFO2 400.2216009 MHz
SI 32768
SF 100.6353990 MHz
WDW EM
SSB 0
LB 1.00 Hz
GB 0
PC 1.40
NH_2
N
180 170 160 150 140 130 120 110 100 90 80 70 60 50 40 30 20 10 0
f1 (ppm)

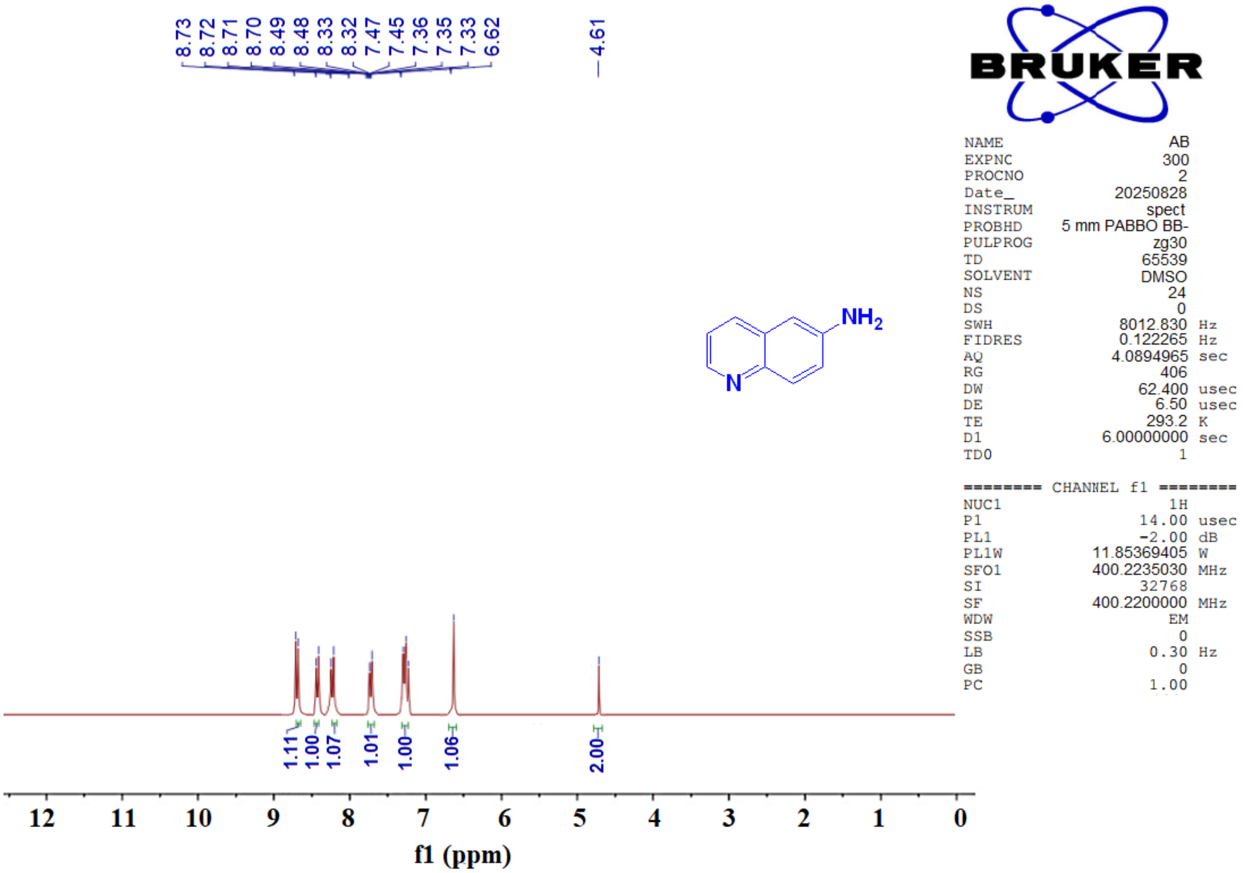

8.73
8.72
8.71
8.70
8.49
8.48
8.33
8.32
7.47
7.45
7.36
7.35
7.33
6.62
4.61
NH2
N
NAME AB
EXPNC 300
PROCNO 2
Date_ 20250828
INSTRUM spect
PROBHD 5 mm PABBO BB-
PULPROG zg30
TD 65539
SOLVENT DMSO
NS 24
DS 0
SWH 8012.830 Hz
FIDRES 0.122265 Hz
AQ 4.0894965 sec
RG 406
DW 62.400 usec
DE 6.50 usec
TE 293.2 K
D1 6.00000000 sec
TD0 1
======== CHANNEL f1 ========
NUC1 1H
P1 14.00 usec
PL1 -2.00 dB
PL1W 11.85369405 W
SFO1 400.2235030 MHz
SI 32768
SF 400.2200000 MHz
WDW EM
SSB 0
LB 0.30 Hz
GB 0
PC 1.00
1.11
1.00
1.07
1.01
1.00
1.06
2.00
12 11 10 9 8 7 6 5 4 3 2 1 0
f1 (ppm)

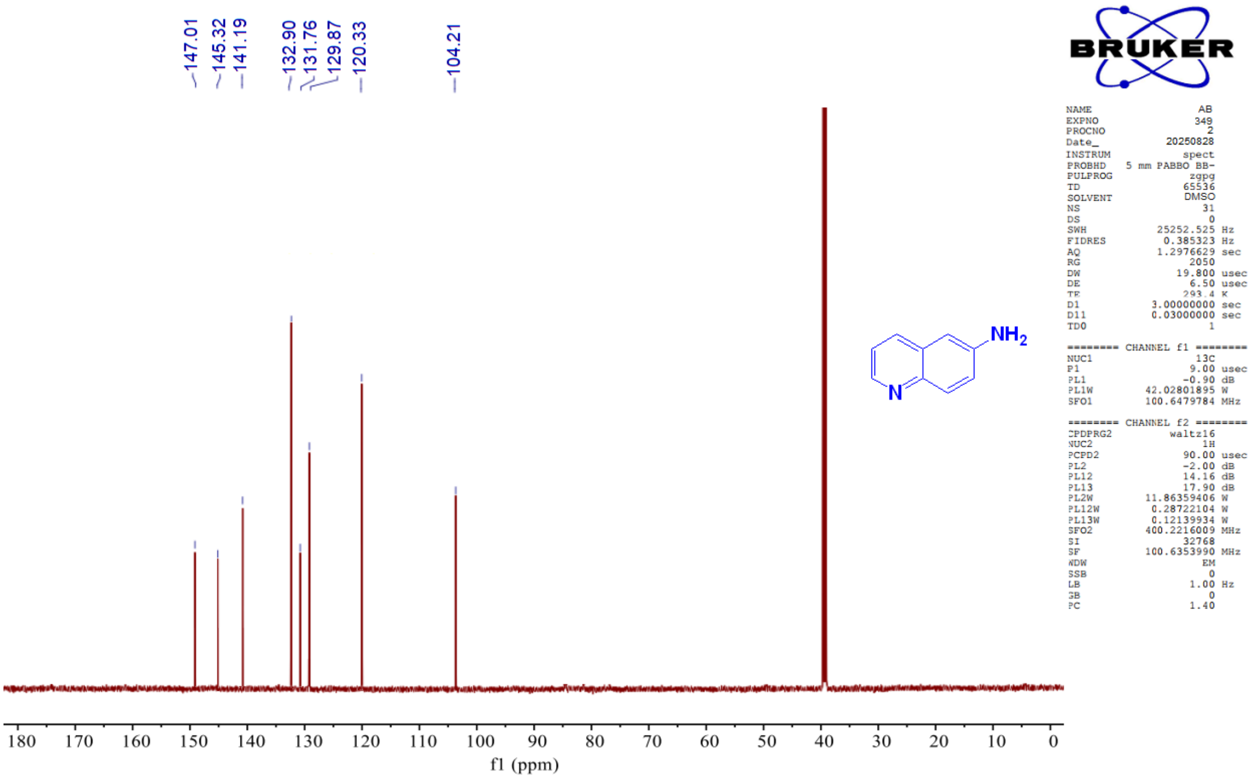

147.01
145.32
141.19
132.90
131.76
129.87
120.33
104.21
NH2
N
NAME AB
EXPNO 349
PROCNO 2
Date_ 20250828
INSTRUM spect
PROBHD 5 mm PABBO BB-
PULPROG zgpg
TD 65536
SOLVENT DMSO
NS 31
DS 0
SWH 25252.525 Hz
FIDRES 0.385323 Hz
AQ 1.2976629 sec
RG 2050
DW 19.800 usec
DE 6.50 usec
TE 293.4 K
D1 3.00000000 sec
D11 0.03000000 sec
TD0 1
======= CHANNEL f1 =======
NUC1 13C
P1 9.00 usec
PL1 -0.90 dB
PL1W 42.02801895 W
SFO1 100.6479784 MHz
======= CHANNEL f2 =======
CPDPRG2 waltz16
NUC2 1H
PCPD2 90.00 usec
PL2 -2.00 dB
PL12 14.16 dB
PL13 17.90 dB
PL2W 11.86359406 W
PL12W 0.28722104 W
PL13W 0.12139934 W
SFO2 400.2216009 MHz
SI 32768
SF 100.6353990 MHz
WDW EM
SSB 0
LB 1.00 Hz
GB 0
PC 1.40
180 170 160 150 140 130 120 110 100 90 80 70 60 50 40 30 20 10 0
f1 (ppm)

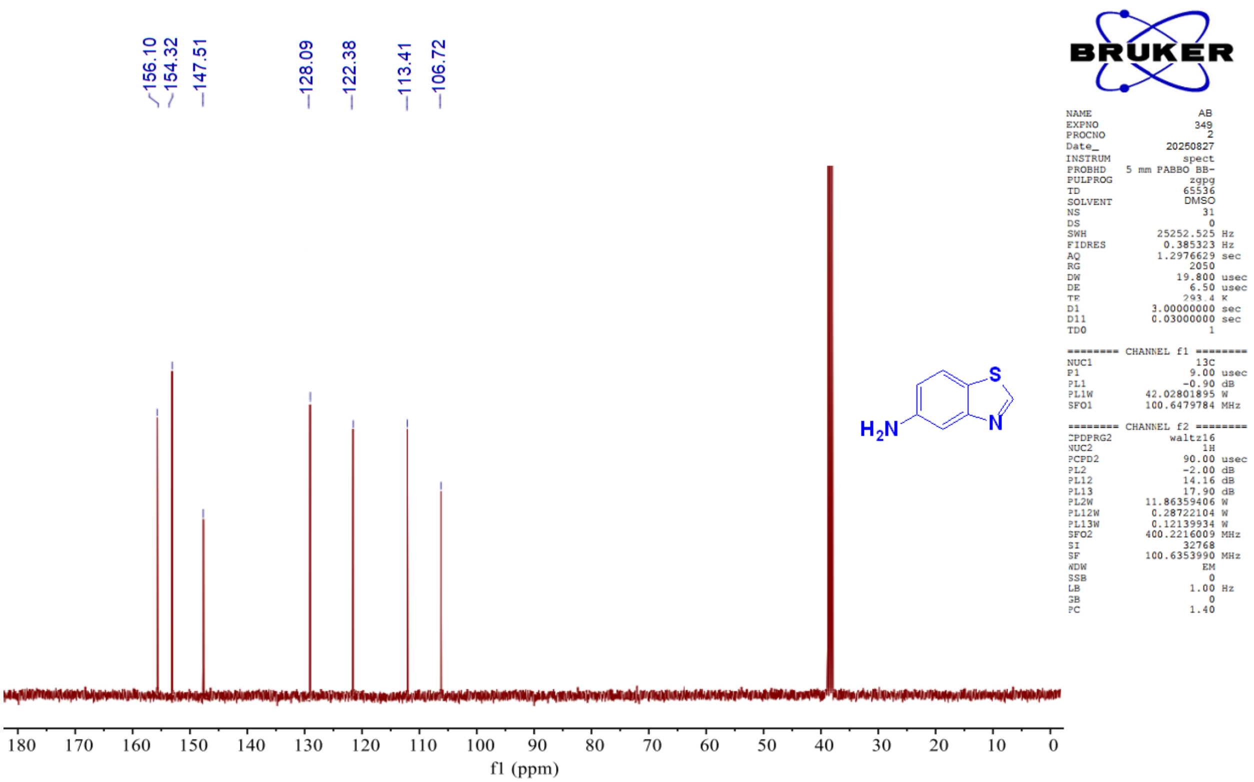
156.10
154.32
147.51
128.09
122.38
113.41
106.72
H2N
S
N
180 170 160 150 140 130 120 110 100 90 80 70 60 50 40 30 20 10 0
f1 (ppm)
BRUKER
NAME AB
EXPNO 349
PROCNO 2
Date_ 20250827
INSTRUM spect
PROBHD 5 mm PABBO BB-
PULPROG zgpg
TD 65536
SOLVENT DMSO
NS 31
DS 0
SWH 25252.525 Hz
FIDRES 0.385323 Hz
AQ 1.2976629 sec
RG 2050
DW 19.800 usec
DE 6.50 usec
D1 3.00000000 sec
D11 0.03000000 sec
TD0 1
======== CHANNEL f1 ========
NUC1 13C
P1 9.00 usec
PL1 -0.90 dB
PL1W 42.02801895 W
SFO1 100.6479784 MHz
======== CHANNEL f2 ========
CPDPRG2 waltz16
NUC2 1H
PCPD2 90.00 usec
PL2 -2.00 dB
PL12 14.16 dB
PL13 17.90 dB
PL2W 11.86359406 W
PL12W 0.28722104 W
PL13W 0.12139934 W
SFO2 400.2216009 MHz
SI 32768
SF 100.6353990 MHz
WDW EM
SSB 0
LB 1.00 Hz
GB 0
PC 1.40

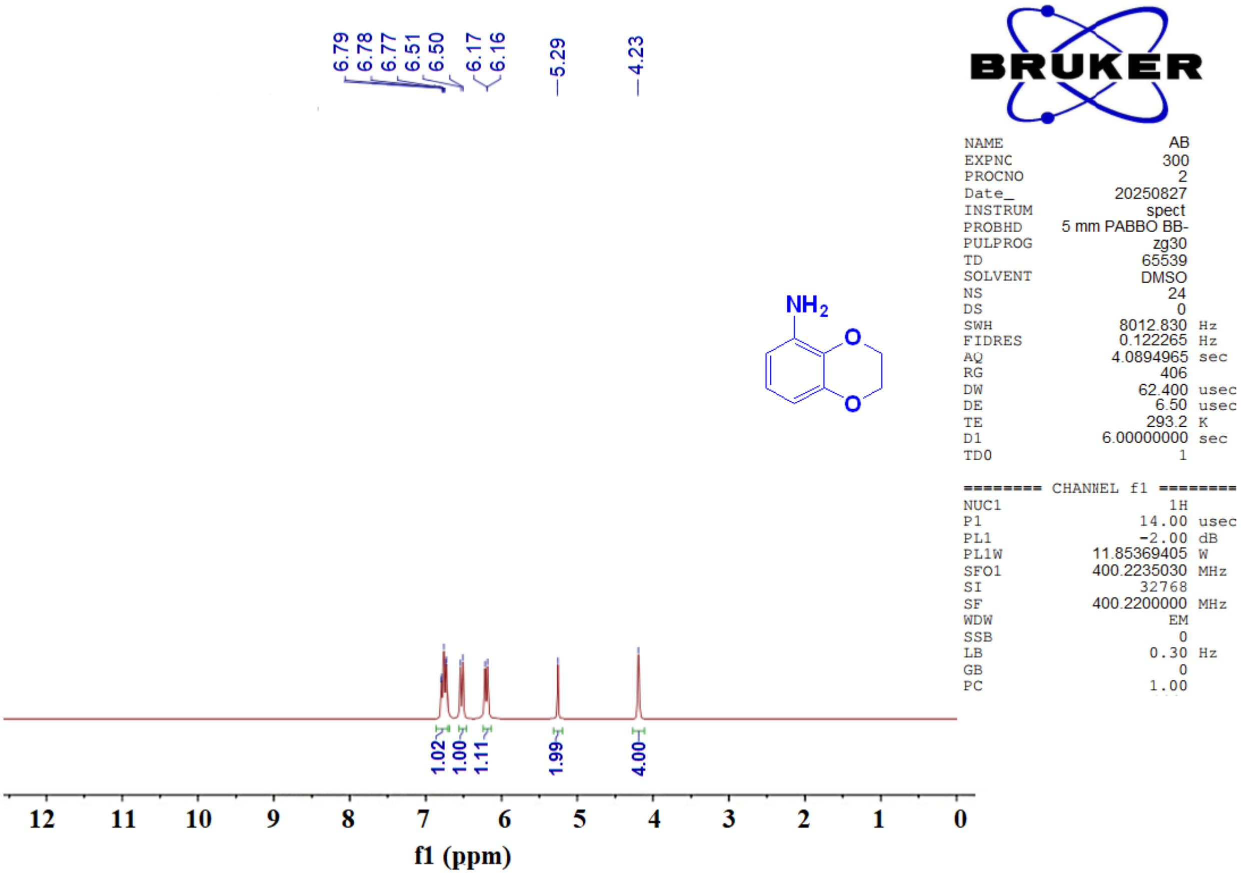
6.79
6.78
6.77
6.51
6.50
6.17
6.16
5.29
4.23
NH2
O
O
1.02
1.00
1.11
1.99
4.00
12 11 10 9 8 7 6 5 4 3 2 1 0
f1 (ppm)
BRUKER
NAME AB
EXPNC 300
PROCNO 2
Date_ 20250827
INSTRUM spect
PROBHD 5 mm PABBO BB-
PULPROG zg30
TD 65539
SOLVENT DMSO
NS 24
DS 0
SWH 8012.830 Hz
FIDRES 0.122265 Hz
AQ 4.0894965 sec
RG 406
DW 62.400 usec
DE 6.50 usec
TE 293.2 K
D1 6.00000000 sec
TD0 1
======== CHANNEL f1 ========
NUC1 1H
P1 14.00 usec
PL1 -2.00 dB
PL1W 11.85369405 W
SFO1 400.2235030 MHz
SI 32768
SF 400.2200000 MHz
WDW EM
SSB 0
LB 0.30 Hz
GB 0
PC 1.00

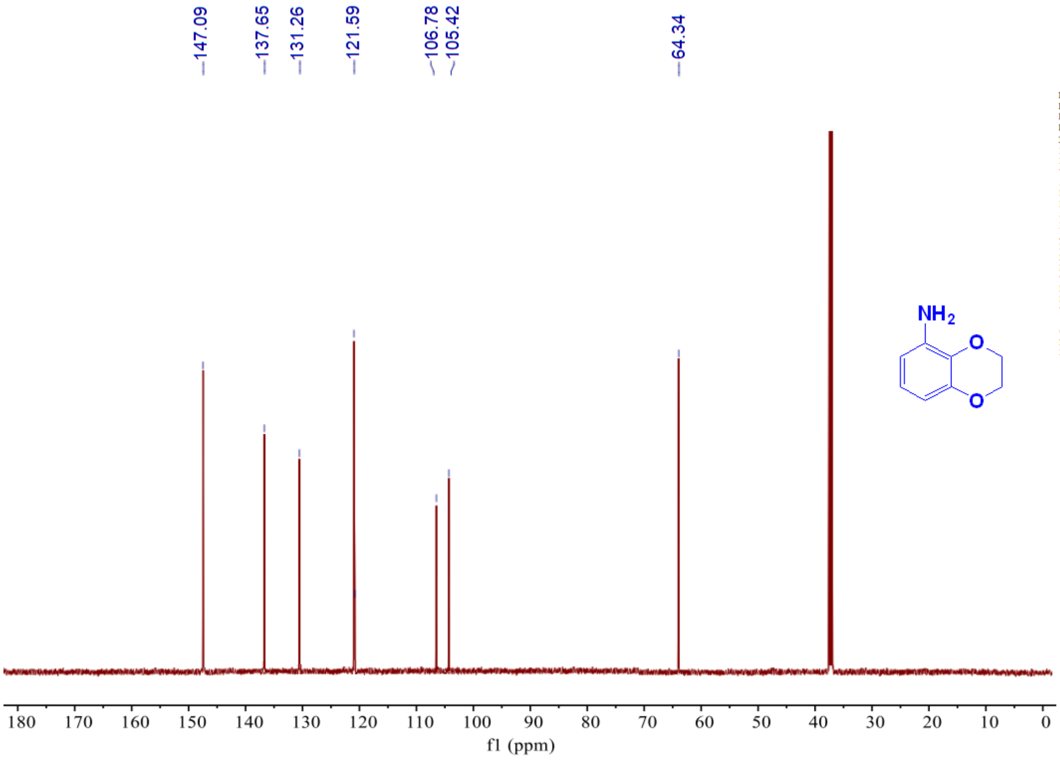


```
NAME                 AB
EXPNO               349
PROCNO                2
Date_          20250827
INSTRUM           spect
PROBHD   5 mm PABBO BB-
PULPROG            zgpg
TD                65536
SOLVENT            DMSO
NS                   31
DS                    0
SWH           25252.525 Hz
FIDRES         0.385323 Hz
AQ            1.2976629 sec
RG                 2050
DW               19.800 usec
DE                 6.50 usec
TE                293.4 K
D1           3.00000000 sec
D11          0.03000000 sec
TD0                   1

======== CHANNEL f1 ========
NUC1                13C
P1                 9.00 usec
PL1               -0.90 dB
PL1W        42.02801895 W
SFO1        100.6479784 MHz

======== CHANNEL f2 ========
CPDPRG2         waltz16
NUC2                 1H
PCPD2             90.00 usec
PL2               -2.00 dB
PL12              14.16 dB
PL13              17.90 dB
PL2W        11.86359406 W
PL12W        0.28722104 W
PL13W        0.12139934 W
SFO2        400.2216009 MHz
SI                32768
SF          100.6353990 MHz
WDW                  EM
SSB                   0
LB                 1.00 Hz
GB                    0
PC                 1.40
```
